# Short-Chain Fatty Acids and Human Health: From Metabolic Pathways to Current Therapeutic Implications

**DOI:** 10.3390/life14050559

**Published:** 2024-04-26

**Authors:** Sonia Facchin, Luisa Bertin, Erica Bonazzi, Greta Lorenzon, Caterina De Barba, Brigida Barberio, Fabiana Zingone, Daria Maniero, Marco Scarpa, Cesare Ruffolo, Imerio Angriman, Edoardo Vincenzo Savarino

**Affiliations:** 1 Department of Surgery, Oncology and Gastroenterology (DISCOG), University Hospital of Padua, 35128 Padua, Italyluisa.bertin.1@studenti.unipd.it (L.B.); brigida.barberio@unipd.it (B.B.);; 2 General Surgery Unit, Department of Surgery, Oncology and Gastroenterology, University of Padova, 35138 Padua, Italycruffolo@hotmail.com (C.R.); imerio.angriman@unipd.it (I.A.)

**Keywords:** intestinal microbiota, short-chain fatty acids (SCFAs), dietary fiber fermentation, gastrointestinal and metabolic health, therapeutic implications

## Abstract

The gastrointestinal tract is home to trillions of diverse microorganisms collectively known as the gut microbiota, which play a pivotal role in breaking down undigested foods, such as dietary fibers. Through the fermentation of these food components, short-chain fatty acids (SCFAs) such as acetate, propionate, and butyrate are produced, offering numerous health benefits to the host. The production and absorption of these SCFAs occur through various mechanisms within the human intestine, contingent upon the types of dietary fibers reaching the gut and the specific microorganisms engaged in fermentation. Medical literature extensively documents the supplementation of SCFAs, particularly butyrate, in the treatment of gastrointestinal, metabolic, cardiovascular, and gut-brain-related disorders. This review seeks to provide an overview of the dynamics involved in the production and absorption of acetate, propionate, and butyrate within the human gut. Additionally, it will focus on the pivotal roles these SCFAs play in promoting gastrointestinal and metabolic health, as well as their current therapeutic implications.

## 1. Introduction

The human gastrointestinal tract harbors a vast population of microbes, numbering in the trillions and spanning hundreds of species, each equipped with a diverse array of hydrolases essential for fermenting indigestible carbohydrates [1]. Microbial fermentation of polysaccharides is most pronounced in the colon, where it achieves a daily production rate of 300 mmol/day, with only 10 mmol/day being excreted [2]. The principal volatile short-chain fatty acids (SCFAs) generated are acetate, propionate, and butyrate, typically in a ratio of 60:25:15 [3]. Butyrate assumes a pivotal role among SCFAs within the intestine, serving as the primary energy source for colonocyte metabolism. It not only enhances the integrity of epithelial tissue but also mitigates mucosal inflammation while promoting electrolyte absorption [4]. Additionally, dissociated butyric acid can readily permeate the cytoplasm, inhibiting DNA replication and disengaging the nutrient transport system from bacteria, thereby exerting a broad-spectrum antibacterial effect [5]. Propionate is believed to confer various benefits upon the gut environment, including the reduction of lipogenesis, cholesterol levels, and carcinogenesis [6]. Furthermore, research indicates that acetate can positively modulate host energy and substrate metabolism within the gut by eliciting the secretion of gut hormones such as glucagon-like peptide-1 and peptide YY [7].

Due to their advantageous properties, SCFAs are frequently utilized as supplements for treating diverse diseases. However, the pharmaceutical formulation of short-chain fatty acid supplements profoundly influences their delivery and absorption. Following an examination of SCFAs production, including acetate, propionate, and butyrate, within the intestinal milieu in the initial section of this review, the subsequent part will delve into the absorption mechanisms of these SCFAs. This section will provide an overview of SCFAs supplements employed in clinical trials, with particular emphasis on their formulations. Additionally, it will explore the significance of SCFAs in gastrointestinal and metabolic health, culminating in an analysis of the existing therapeutic implications. The aim of the review is to provide a comprehensive understanding of the role of SCFAs in human gastrointestinal and metabolic health, with a particular focus on their production, absorption mechanisms, and therapeutic implications.

## 2. Methods

### 2.1. Literature Search Strategy

A comprehensive literature search was conducted to identify relevant articles pertaining to the role of SCFAs in human gastrointestinal and metabolic health. Searches were performed across electronic databases, including PubMed, MEDLINE, and Google Scholar, using appropriate keywords and Boolean operators such as “short-chain fatty acids”, “SCFAs”, “butyrate”, “propionate”, “acetate”, “gastrointestinal health”, “metabolic health”, “supplement”, “colorectal cancer”, “irritable bowel syndrome”, “inflammatory bowel disease”, “disorders of the gut-brain axis”, “disorders of the gut-brain interactions”, and “therapeutic implications”. The search was limited to articles published in English mostly within the last decade. Some articles of fundamental importance can date back more than forty years, demonstrating the long period of interest around the topic. Additionally, references cited in selected articles were manually screened to identify additional relevant studies.

### 2.2. Inclusion and Exclusion Criteria

Articles were included if they presented findings relevant to SCFAs in human gastrointestinal and metabolic health. Both clinical and preclinical studies were considered. Exclusion criteria encompassed non-English publications, reviews, commentaries, and studies not directly aligned with the scope of the review.

### 2.3. Data Extraction and Analysis

Data from selected articles were systematically extracted. Key information included study characteristics, participant demographics (if applicable), SCFAs interventions (if applicable), outcome measures, and main findings related to gastrointestinal and metabolic health. Data synthesis was performed narratively, with studies grouped based on their thematic relevance to the review’s objectives. Key findings were summarized, and emerging themes were identified. Any discrepancies or conflicting results were noted and discussed within the context of the review.

### 2.4. Quality Assessment

Given the narrative nature of the review, formal quality assessment tools were not employed. However, the credibility and reliability of included studies were considered during data synthesis and interpretation.

## 3. Results

### 3.1. Production of SCFAs in the Gastrointestinal Tract

#### 3.1.1. Cross-Feeding and Production of SCFAs in the Human Intestine

Microbial communities are shaped by a spectrum of interactions, encompassing both positive and negative dynamics, ranging from competition to mutualism. Within the mammalian gut, a plethora of microbial inhabitants coexist, and the intricate interplay among these microbes gives rise to synergistic responses [8]. Numerous ecological processes are orchestrated by diffusible metabolites, which serve multifaceted roles as nutrient reservoirs, inhibitory agents, or signaling messengers [8]. Among these processes, cross-feeding emerges as a pivotal mechanism, facilitating the exchange of metabolites for energy and nutrients among diverse microbial species or strains [9]. Furthermore, various forms of cross-feeding occur within the gut microbiome, including parasitism, commensalism, and mutualism. Parasitism ensues when one microbe benefits from a substrate produced by another organism while concurrently altering the environment to the detriment of the producer. On the other hand, mutualism involving cross-feeding occurs when two or more species exchange resources or metabolic byproducts with each other, resulting in mutual benefit. Finally, commensalism cross-feeding involves a relationship between two organisms of different species in which one benefits, and the other is neither helped nor harmed. For many species, the exchange of fermentative intermediates plays a vital role in their gut ecosystem. Key fermentative intermediates include SCFAs and carboxylic acids with a brief aliphatic tail comprising six carbons, notably acetate (C2), propionate (C3), and butyrate (C4). These metabolites are generated by certain bacterial species under anaerobic conditions through the fermentation of dietary fibers, predominantly oligofructose, arabinoxylan, inulin, and pectin [10]. Additionally, environmental factors such as the relatively low pH (5.5) likely contribute to shaping the community structure and microbial activities in the colon. This ecological consideration becomes significant in facilitating the competitive advantage of butyrate-producing bacteria over carbohydrate-utilizing bacteria, such as *Bacteroides* spp., which thrive at a pH closer to 6.5.

#### 3.1.2. Production of Acetate by the Intestinal Microbiota

Acetate stands out as a primary fermentation byproduct for the majority of gut anaerobes, consistently achieving the highest concentration among SCFAs in the gut lumen [11]. Microbial-derived acetate production arises from the fermentation of indigestible foods, particularly those rich in acetogenic fibers such as galactooligosaccharides and inulin [12]. The microbial fermentation of acetogenic fibers leads to acetate production through two metabolic pathways: acetogenesis and carbon fixation. Acetogenesis involves the production of acetate, facilitated by homoacetogenic bacteria or acetogens capable of synthesizing acetate from H_2_ and CO_2_. Meanwhile, the carbon fixation pathway produces acetate from CO_2_ as a precursor, also known as the Wood-Ljungdahl pathway [13]. This pattern is notably accompanied by an increase in the *Firmicutes/Bacteroidetes* ratio and cross-feeding mechanisms, exemplified by the upregulation of pyruvate fermentation pathways to acetate and lactate by *Lactobacillus reuteri* and other unclassified bacteria [14]. Various studies corroborate these findings, highlighting an augmented abundance of crucial acetate producers, such as *Akkermansia muciniphila*, during human fasting and caloric restriction interventions [15,16]. This intermediary holds particular significance as it can undergo further metabolism by acetate-consumers, such as *Faecalibacterium prausnitzii* and *Roseburia intestinalis/Eubacterium rectale*, to produce butyrate [17]. Notably, acetate has been identified as a growth requirement for these bacteria [17,18], thereby establishing its status as an essential intermediary within the intestine.

#### 3.1.3. Production of Propionate by the Intestinal Microbiota

Propionate, an SCFA, primarily derives from two essential pathways facilitated by the fermentation of various carbohydrates by gut bacteria. The succinate pathway involves the fermentation of hexose and pentose sugars, yielding propionate, while the propanediol pathway produces propionate from the fermentation of fructose and rhamnose. The former pathway is predominantly associated with *Bacteroidetes* and the *Negativicutes* class of *Firmicutes* [18], serving as the primary route for propionate formation from dietary carbohydrate fermentation, primarily propelled by the abundance of *Bacteroidetes*. Succinate serves as a precursor to propionate, with its conversion necessitating vitamin B12. Propionate formation from rhamnose and fructose has been observed in gut bacteria belonging to the Lachnospiraceae family, such as *Roseburia inulinivorans* and *Blautia* spp. [18].

Besides carbohydrates, peptides and amino acids can also serve as precursors for propionate formation, albeit amino acid-fermenting bacteria are estimated to comprise less than 1% of the large intestinal microbiota. Notably, *Bacteroidetes* are primarily responsible for propionate formation via proteolysis of peptides and amino acids [19]. In vitro incubations of fecal slurries with individual amino acids indicate that propionate predominantly derives from aspartate, alanine, threonine, and methionine [20].

Furthermore, cross-feeding among different commensal gut bacteria plays a crucial role in propionate production. Bacteria such as *Bacteroides* spp., *Escherichia coli*, and *Anaerostipes rhamnosivorans* degrade deoxy sugars to produce the pathway intermediate 1,2 propanediol, with *Eubacterium hallii* and *Lactobacillus reuteri* further metabolizing this intermediate to produce propionate [21].

While propionate is less extensively researched compared to other microbial metabolites like butyrate, studies have shown its distinct health-promoting properties. These include cholesterol-lowering and antilipogenic effects, stimulation of satiety, and protection against colorectal cancer (CRC) [22]. Its beneficial effects in the context of gastrointestinal diseases, particularly inflammatory bowel diseases (IBD) and irritable bowel syndrome (IBS), will be further elaborated in the subsequent section of this review.

#### 3.1.4. Production of Butyrate by the Intestinal Microbiota

Butyrate can be produced from butyryl-CoA via two distinct enzymatic routes. The enzymes responsible for this conversion are butyrate kinase and butyryl-CoA:acetate CoA transferase (BCoAT). These enzymes facilitate the transformation of butyryl-CoA into butyrate, albeit employing slightly different mechanisms. Nevertheless, within the human colonic ecosystem, BCoAT stands out as the primary enzyme accountable for this conversion.

In various studies, researchers have observed the production of butyrate in organisms such as *Eubacterium* spp., *Roseburia* spp., *Anaerostipes* spp., and *Faecalibacterium prausnitzii* [3]. Another pathway for butyrate synthesis involves the enzymes butanoyl-CoA:phosphate butanoyltransferase and butyrate kinase. For instance, certain species of Coprococcus and numerous Clostridium species within the Firmicutes family employ butyrate kinase for butyrate production [23]. Within the Firmicutes phylum, *Ruminococcaceae* and *Lachnospiraceae* are the most prominent families of butyrate producers. *Faecalibacterium prausnitzii*, a member of the Ruminococcaceae family, stands out as one of the most abundant species in the healthy human microbiota [19]. As previously mentioned, *Faecalibacterium prausnitzii* synthesizes butyrate via BCoAT, utilizing acetate as a substrate, thereby promoting its growth on carbohydrate energy sources [17]. Its anti-inflammatory properties in the intestine have sparked increasing interest in recent years, making it a potential therapeutic agent for patients suffering from IBD, who often exhibit depleted levels of *F. prausnitzii* [24].

Butyrate-producing Lachnospiraceae exhibit significant diversity in phylogeny, gene organization, and physiology [25]. *Eubacterium rectale* and *Roseburia* species, closely related members of this family, constitute a substantial portion of butyrate producing Firmicutes through the BCoAT pathway. Interestingly, certain strains of *Roseburia* primarily produce butyrate under mildly acidic pH conditions, consuming acetate in the process. However, other strains also produce formate and lactate alongside butyrate [25]. Additionally, select members of Lachnospiraceae, such as *Anaerostipes hadrus* and *Eubacterium hallii*, possess the ability to utilize lactate for butyrate production [26].

Furthermore, butyrate can be generated through the fermentation of peptides and amino acids. For instance, *Intestinimonas butyriciproducens AF211* ferments lysine to produce butyrate [27]. Moreover, various pathways exist for glutamate degradation to butyrate in butyrate-producing bacteria. These pathways involve intermediates entering the main butyrate synthesis pathway via pyruvate (e.g., *Fusobacterium* spp., *Clostridium limosum*) or crotonyl-CoA (found in various Firmicutes, including *Acidaminococcus symbiosum*, *Clostridium sporosphaeroides*, *Clostridium symbiosum*, etc.). While the fermentation pathways of other amino acids are less extensively characterized [28], evidence suggests that histidine can be converted to glutamate, which is subsequently fermented to butyrate by the intestinal microbiota [29,30]. A summary of the production of the three different SCFAs and the metabolic pathways and bacteria involved is provided in Table 1.

#### 3.1.5. Cross-Feeding Lays the Basis of Butyrate Production by Intestinal Microbiota

As previously discussed, the production of SCFAs and other intermediates is reliant on dietary fibers, and to a lesser extent, on peptides and amino acids metabolized by intestinal bacteria. Dietary fibers belong to the category of prebiotics, which are non-digestible food ingredients stimulating the growth and/or activity of bacteria in the intestine, thereby benefiting the consumer [31]. Industrial examples of prebiotics include inulin-type fructans, galactooligosaccharides (GOS), and fructooligosaccharides (FOS) [32].

Inulin-type fructans, for instance, occur naturally in various vegetables such as onion, garlic, leek, banana, and chicory root. They consist of short and long polymers of fructose with varying degrees of polymerization. These compounds remain undigested and unabsorbed in the human gastrointestinal tract, making them available for fermentation by bacteria, primarily Bifidobacteria, in the colon [33]. The growth of Bifidobacteria further stimulates the growth of butyrate-producing bacteria, known as the butyrogenic effect, through cross-feeding [34].

During the intricate process of colon fermentation, inulin-type fructans are primarily converted into SCFAs and other organic acids (e.g., lactate and succinate) as well as gases (hydrogen and carbon dioxide) [35]. Cross-feeding, initiated from beta-fructans, involves two main types: one entails the fermentation of short oligosaccharides or monosaccharides released by *Bifidobacterium* from the prebiotic substrate, while the other begins with the fermentation of acetate and lactate [36].

For instance, *Eubacterium hallii* DSM 17630 efficiently converts lactate and acetate produced by *Bifidobacterium adolescentis* DSM 20083 into butyrate when grown in co-culture with oligofructose present. Similarly, in a co-culture of *Anaerostipes caccae* DSM 14662 and *Bifidobacterium longum* BB536, the former ferments acetate and fructose produced by B. longum during substrate breakdown [37].

Although clostridiales species constitute a minor fraction of the human colon microbiota (5–10%), butyrate formation by strictly anaerobic bacteria, including the *Clostridium* genus, has long been recognized. Specifically, over 90% of colonic butyrate-producing bacteria are represented by *Faecalibacterium prausnitzii* (*Clostridium leptum* cluster) and *Eubacterium/Roseburia* spp. (*Clostridium coccoides* cluster). The rate of butyrate formation by acetate-consumers (e.g., *Faecalibacterium prausnitzii* and *Roseburia intestinalis*) can vary depending on the species of butyrate-producing bacterium and the type of fermentable carbohydrate [8].

In addition to inulin-type fructans, the butyrogenic effect is also evident in resistant starch fermentation [38]. In vitro studies have shown efficient butyrate production in a co-culture of *B. longum* JCM 1217 and *Eubacterium limosum* JCM 6421 on germinated barley. Here, *E. limosum* utilizes lactate for butyrate production via cross-feeding, as lactate is previously produced by B. longum during starch degradation [39].

**Figure 1:**
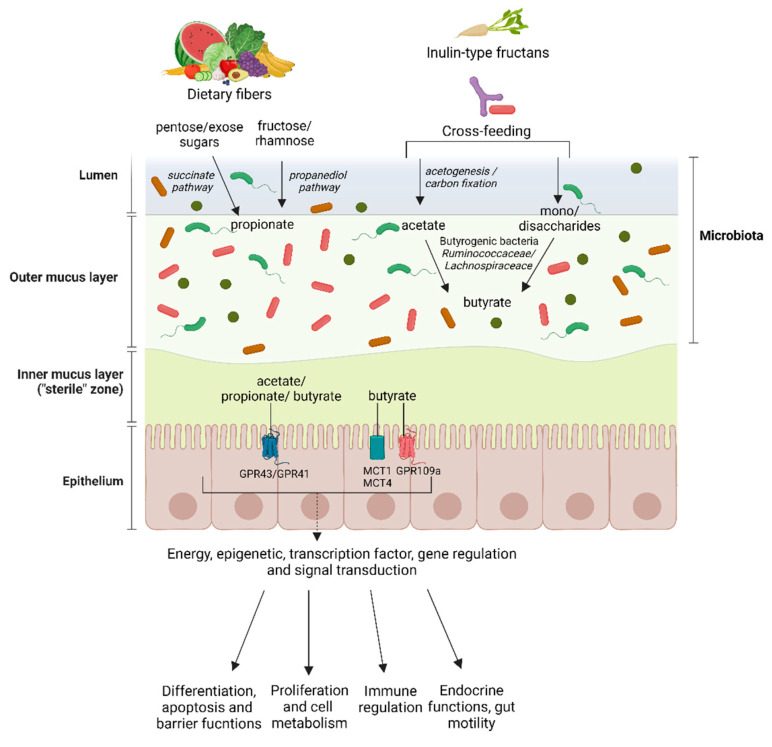
Production, absorption, transport and potential effects of the SCFAs acetate, propionate and butyrate in the human gut. Dietary fibers lead to a higher production of propionate and acetate, while butyrate is primarily obtained through cross-feeding and transformation of other SCFAs. Butyrate, acetate and propionate share some common transporters and are predominantly absorbed through a facilitated process. GPR43 refers to G-protein-coupled-receptors 43, GPR41 to G-protein-coupled-receptors 41, GPR109a refers to G-protein-coupled-receptors 109a, MCT1 to monocarboxylate transporters 1, MCT4 to monocarboxylate transporters 4.

Another study recently highlighted efficient cross-feeding between *Roseburia intestinalis* (a butyrate producer) and *Ruminococcus hydrogenotrophicus* (an acetate producer) during xylan growth. Initially, xylan degradation is facilitated by *Roseburia intestinalis*, which produces carbon dioxide and hydrogen, serving as substrates for *Ruminococcus hydrogenotrophicus* growth alongside acetate production. This SCFA then acts as an essential co-substrate for butyrate production [40]. Figure 1 provides an overview of SCFAs production in the gut by the microbiota.

As mentioned earlier, researchers have recently focused on studying butyrate due to its beneficial properties in the intestinal environment. Butyrate has been reported to play crucial roles in intestinal cell development and gene expression [4,41], and it is generally believed to have a protective effect against CRC and colitis. The specific beneficial roles of butyrate in gastrointestinal and metabolic diseases will be explored further in the third part of this review.

### 3.2. Absorption of SCFAs in the Intestine and SCFAs Supplements

#### 3.2.1. Absorption of Butyrate

For many years, it was believed that butyrate absorption primarily occurred through passive diffusion in its liposoluble form [41]. However, contemporary evidence strongly suggests that SCFAs, including butyrate, are predominantly absorbed via a facilitated process involving a series of transport proteins. The characterization of several transmembrane proteins has led to the identification of two well-defined absorption pathways, both involving monocarboxylate transporters: MCT1 and MCT4 [42,43], two hydrogen-coupled transporters, and SMCT1, a sodium-coupled monocarboxylate transporter [44].

Early studies by Thibault et al., assessing butyrate absorption in diseased colon tissue from patients with IBD, familial adenomatous polyposis (FAP), and CRC, highlighted a drastic reduction in MCT1 mRNA in diseased tissues, correlating with the degree of inflammation. Functionally, this reduction was demonstrated by a decrease in butyrate absorption and metabolism [45]. Notably, in cancerous tissue, MCT1 expression exhibits peculiarities: while it decreases during the transition from normal to malignancy, being downregulated in the early stages of carcinogenesis [46], a subsequent upregulation of MCT1 has been observed in advanced metastatic CRC tumors. In these tumors, MCT1 and MCT4 transporters play a crucial role in lactate transport and, consequently, intracellular pH regulation. Inhibiting the MCT1 receptor reduces intracellular pH, leading to tumor cell death. Thus, MCT1 and MCT4 emerge as potential therapeutic targets in cancer treatment [47,48,49]. Butyrate has previously been approved for clinical use in CRC treatment [50], as it is a substrate for MCT1 and MCT4, well metabolized, and has shown no reported side effects until now [51]. In contrast to the MCT1 receptor, knowledge regarding the regulation of SMCT1 at the intestinal level remains limited. SMCT1 is downregulated during intestinal inflammation, and its expression is often silenced in aberrant crypt foci, colon adenomas, colon tumors, and colon cancer cell lines, suggesting that SMCT1 silencing is an early event in colon tumorigenesis. It has been proposed that SMCT1 functions as a tumor suppressor, and its ability to mediate butyrate entry into colonocytes underlies its potential tumor-suppressive function [52].

Additionally, among the regulatory and interaction systems involving butyrate, the efflux transporters, capable of removing butyrate from cells, are noteworthy. Among these, Breast Cancer Resistance Protein (BCRP) is believed to limit drug absorption, bioavailability, and toxicity. Butyrate is a substrate for BCRP [53], and the inhibition of BCRP has significantly potentiated the inhibitory effect of butyrate on cell proliferation [54]. Following absorption, butyrate signals through three membrane G-protein-coupled receptors (GPCRs): GPR41, GPR43, and GPR109A, present on the surface of colon cells, adipocytes, and immune cells. These receptors modulate cytokine levels and various signaling pathways when activated, promoting an anti-inflammatory response [55].

#### 3.2.2. Butyrate Supplements

The literature commonly discusses studies that use two different formulations of butyrate: calcium butyrate (CaBu) and sodium butyrate (NaBu). Both sodium butyrate and calcium butyrate are derivatives of butyric acid but exhibit differences in the metal ion with which they are associated. Calcium and sodium are the primary cations found in the extracellular space, with calcium demonstrating lower water solubility compared to sodium [56]. The selection of a particular butyrate formulation, along with its associated metal ion, could hold significance in the treatment of patients with specific medical conditions or deficiencies [57].

The formulation of CaBu combined with vitamin D presents a particularly intriguing prospect, especially in the realm of cancer prevention [58]. Depending on the inflammatory context, NaBu formulations may contribute to protective immunity relative to the associated ion [57]. Research has shown that the storage of sodium in tissues enhances defense against invasive pathogens [59]. However, immune activation induced by sodium salt may also have a negative impact on wound healing [60]. It is important to note that the concentration of salts combined with butyrate generally ranges in the order of a few milligrams, depending on the formulation under investigation.

In addition to the formulation, the type of pharmaceutical form used for product delivery should also be carefully evaluated based on the site of action and the desired effect.

Generally, butyrate used in clinical studies has shown beneficial effects on the intestinal level [61]. However, some in vitro studies and some studies conducted in animal models [62] have shown that butyrate enemas administered for three consecutive days induced concentration-dependent colon hypersensitivity (from 3–8 up to 1000 mmol/L) and mechanical hyperalgesia, but no macroscopic and histological modification of the colon mucosa. This condition mimics the clinical presentation observed in patients with IBS and serves as a model of chronic non-inflammatory colon hypersensitivity. However, in human subjects, administration of butyrate in the distal colon leads to a decrease in pain and discomfort, a stark contrast to findings in rat studies. Some researchers attribute this disparity to differential modulation of butyrate-coupled receptors in rats and humans, as well as variations in butyrate concentration between exogenous administration and endogenous production in the colon [63].

It is reasonable to speculate that the pharmaceutical formulation may influence the concentration of butyrate in the colon, thereby exerting a pharmacological effect on the underlying pathology [64]. Oral formulations employing gastro-resistant capsules, microencapsulation, or enemas (refer to Table 2) may produce divergent effects across different bodily regions, owing to variations in the release kinetics of butyrate [65]. Unlike gastro-resistant capsules, lipid microencapsulation not only masks the unpleasant odor associated with rancid butter, a characteristic of butyrate compounds, but also protects them from gastric acid hydrolysis, ensuring their delivery to the small intestine and colon, where they can exert their therapeutic effects.

Emerging cream formulations solely based on butyrate [93], devoid of corticosteroids, are currently available on the market and hold promise in managing local inflammations, mitigating the side effects associated with corticosteroid use [94]. However, as of now, there is a paucity of clinical studies assessing their efficacy.

#### 3.2.3. Absorption of Propionate

Propionate has been associated with reductions in lipogenesis and serum cholesterol levels [95], exerting beneficial effects on weight control and eating behavior [96]. Additionally, studies have demonstrated that, akin to butyrate, propionate exerts an antiproliferative effect on colon tumor cells [97]. The production of propionate by intestinal bacteria involves the transformation of prebiotic compounds such as L-rhamnose, D-tagatose, inulin, resistant starch, polydextrose, and arabinoxylans [6]. However, comparative assessments of propionate’s modulatory effects on such compounds are challenging due to the heterogeneity of experimental designs across studies. It is important to note that establishing a direct connection between the production of SCFAs and their concentration in the intestinal lumen is only feasible in an in vitro context without intestinal absorption.

The mechanisms of propionate production entail specific fermenting bacteria utilizing distinct metabolic strategies, as previously mentioned. The propionate thus produced is readily transported systemically, traversing the liver [98,99]. Generally, propionate and acetate can activate GPR41 and GPR43 cell surface receptors but can also be efficiently absorbed at the cellular level, circumventing SCFA receptors on the cell surface. Studies have indicated that propionate enhances the differentiation of T cells into effector cells such as T-helper 1 cells (Th1) and T-helper 17 cells (Th17), favoring regulatory T cells that produce anti-inflammatory IL-10 [100]. This regulatory process is crucial for maintaining intestinal homeostasis and preventing chronic inflammation such as that encountered in IBD.

#### 3.2.4. Propionate Supplements

Unlike butyrate, formulations of propionate for supplementation have been the subject of limited study in clinical trials, particularly in the realms of obesity, diabetes, and cardiovascular disease (refer to Table 3). A recent study investigated the supplementation of propionic acid, administered twice daily via 500 mg capsules over a 14-day treatment period in patients with multiple sclerosis (MS). Results demonstrated a significant 30% increase in Treg cells compared to baseline, along with a reduction in Th17 cells [101]. These findings were associated with a reduction in relapses and stabilization of disability, indicating promising therapeutic potential.

In a crossover randomized controlled trial (RCT), overweight adult subjects were administered an inulin-propionate ester formulation for 24 weeks. The study confirmed that increased propionate levels in the colon effectively prevented weight gain in enrolled subjects [102]. Currently, two clinical trials are underway to evaluate the effect of sodium propionate in subjects with various pathologies, albeit none specifically in the field of gastroenterology (refer to Table 4).

Considering the mounting clinical evidence supporting the immunomodulatory effects of propionate, there is a pressing need for further well-structured clinical studies, particularly in the context of chronic intestinal inflammations.

#### 3.2.5. Absorption of Acetate

While less extensively studied compared to butyrate, acetate holds notable interest due to its lower toxicity to epithelial cells, its ability to stimulate bacteria that produce butyrate through cross-feeding, and its anti-inflammatory and protective properties [106]. Receptors such as GPR43, pivotal in maintaining calcium homeostasis, are receptive to acetate and propionate [107]. The probiotic activity of *Saccharomyces cerevisiae* var. *boulardii* is thought to be closely linked to its notably high acetate production [108]. Although the mechanism of acetate’s action on intestinal cells is not fully elucidated, its positive impact on body weight regulation is noteworthy. In murine models, acetate administration has demonstrated effects on energy intake and expenditure, influencing body weight control [109]. However, human studies investigating long-term oral acetate supplementation or endovenous/gastric infusion in the colon with weight loss and energy expenditure as primary outcomes are limited [7], and cross-sectional/cohort analyses have yielded inconsistent results regarding obesity and adiposity [85]. The primary dietary sources of acetate include dairy products, pasta, bread, eggs, smoked fish, and coffee [110]. Other significant sources encompass ethanol, vinegar, and microbial production obtained from the fermentation of indigestible carbohydrates, particularly acetogenic fibers such as inulin and galactooligosaccharides [12].

#### 3.2.6. Acetate Supplements

The predominant formulations utilized in clinical studies are inulin acetate ester and sodium acetate, administered via enema in the proximal colon. Similar to findings observed with propionate, investigations involving acetate in clinical settings typically focus on the effects of oral supplementation of fermented foods on weight management rather than direct implications for gastrointestinal disorders [109]. Table 5 delineates the outcomes of acetate interventions in hyperinsulinemic females.

## 4. Implications of SCFAs in Human Gastrointestinal and Metabolic Health

Several studies have indicated the involvement of SCFAs in human GI and metabolic health. SCFAs are thought to have pleiotropic effects on gastrointestinal and metabolic health. The identified signaling mechanisms of SCFAs may function through two main mechanisms. The first is via interactions with GPCRs, as previously described, expressed in various organs, including the intestine, kidney, and heart [115,116,117]. These receptors are expressed in various cell types within the gastrointestinal tract, including enterocytes, enteroendocrine cells, immune cells, and neuronal cells, mediating a range of physiological responses [117]. The second acts as (HDACs) inhibitor [118,119], promoting gene expression and regulating cell metabolism, differentiation, and proliferation by inhibiting specific gene transcription [120,121,122].

### 4.1. Gastrointestinal Diseases

SCFAs play a critical role in maintaining gut health and have been implicated in various gastrointestinal diseases, including IBD, CRC, and disorders of the gut-brain axis. The supposed mechanisms of SCFAs are summarized in Table 6.

#### 4.1.1. Inflammatory Bowel Disease

The interaction between SCFAs and IBD is multifaceted, involving the interplay of gut microbiota, immune responses, and the integrity of the gut epithelial barrier [123,124]. Butyrate, a primary energy source for colonocytes, exerts anti-inflammatory effects by inhibiting the activation of the nuclear factor kappa B and reducing proinflammatory gene expression [125]. A decline in SCFAs-producing bacteria characterizes IBD patients, notably butyrate producers like *Faecalibacterium prausnitzii* and *Roseburia hominis* [126,127,128]. This results in reduced colonic SCFAs levels linked to compromised gut barrier function in IBD [129].

SCFAs protect against IBD-associated intestinal inflammation through various mechanisms [130]. They enhance the intestinal epithelial barrier by promoting mucus production and tightening tight junctions between epithelial cells [130]. Additionally, SCFAs modulate immune responses by influencing the differentiation and function of Tregs, suppressing excessive immune reactions [131]. Several pathways are involved in SCFAs-mediated immune regulation, including GPCRs, HDACs, and the regulation of innate immune sensors like Toll-like receptors (TLRs) and Nod-like receptor family pyrin domain containing 3 (NLRP3) inflammasome. SCFAs inhibit the progression of IBD by regulating innate immune sensors, TLRs, and NLRP3 inflammasomes. SCFAs protect the intestinal barrier; acetate, propionate, and butyrate stimulate the intestinal NLRP3 inflammasome, increasing IL-18 secretion and enhancing intestinal barrier integrity [132]. Moreover, SCFAs engage with GPR43 and GPR109A receptors essential for regulating intestinal immunity, stimulating the production of Treg. This has been demonstrated in preclinical studies, where controlling colonic Treg levels and function in a GPR43-dependent manner has been shown to mitigate inflammation, as seen in SCFAs-mediated protection against colitis in GPR43-deficient (Gpr43(−/−)) mice [133,134]. Furthermore, SCFAs promote the differentiation of Tregs by inhibiting HDACs activity, and Tregs secrete protective cytokines, such as IL10, to suppress inflammation [135]. SCFAs not only inhibit TLR signaling, but butyrate acts as an HDACs inhibitor to suppress TLR4 expression and the TLR2-mediated release of inflammatory factors [136,137,138]. Finally, SCFAs participate in tissue repair processes within the gut, promoting the proliferation and differentiation of epithelial cells, thus facilitating the healing of damaged tissues caused by inflammation in IBD [139].

A recent study investigated the utility of fecal SCFAs concentrations as surrogate markers for gut microbiota diversity in patients with IBD and primary sclerosing cholangitis (PSC) [140], resulting in decreased fecal isobutyrate levels compared to healthy controls. Fecal acetate and butyrate positively correlated with fecal calprotectin and serum C-reactive protein in ulcerative colitis (UC) patients. Furthermore, UC patients with higher fecal calprotectin levels exhibited elevated fecal acetate, butyrate, and propionate levels. These findings suggest potential associations between SCFAs levels and disease activity in UC patients.

**Figure 2:**
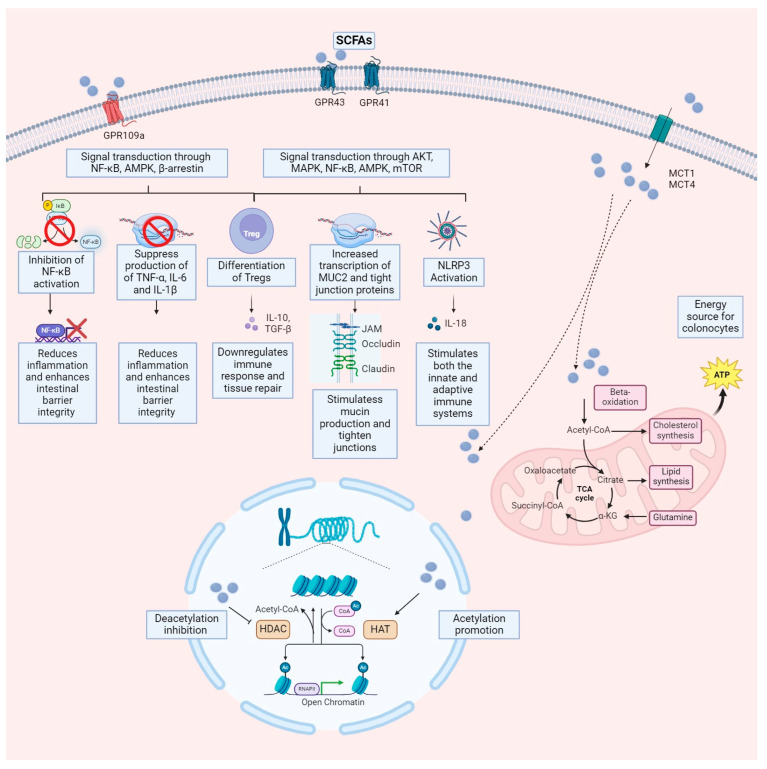
Mechanisms by which SCFAs exert their effects on target cells. SCFAs enter cells via MCT and transporters located on the cell membrane. Once inside the cell nucleus, they inhibit HDAC and activate HAT, facilitating histone acetylation. This process gradually relaxes compacted chromosomes, ultimately resulting in increased gene expression. Additionally, upon entering colonocytes, SCFAs may undergo beta-oxidation and enter the mitochondria, where the citric acid cycle (also known as the Krebs cycle) generates energy for the cell. Another mechanism involves SCFAs binding to GPCR, such as GPR43, GPR41, or GPR109A, on the cell membrane of both colonocytes and immune cells. This interaction inhibits downstream signaling pathways, including NF-κB, Akt, MAPK, and mTOR, while activating the 5’ adenosine monophosphate-activated protein kinase (AMPK) pathway. Consequently, this regulates gene transcription and translation, leading to inflammation mitigation, oxidative stress reduction, and autophagy enhancement. AKT refers to the activation of a serine/threonine kinase; NF-κB to nuclear factor-κB; AMPK to adenosine 5′-monophosphate (AMP)-activated protein kinase; MAPK to mitogen-activated protein kinase; NLRP3 to nucleotide-binding oligomerization domain (NOD), leucine-rich repeat (LRR)-containing proteins (NLR); mTOR to mammalian target of rapamycin. The figure was created using BioRender.com (accessed on 25 March 2024).

Although SCFAs concentrations are decreased in IBD patients, SCFAs supplementation through diet or probiotics shows promise as an adjunct therapy, with minimal adverse effects reported [126,139,141,142,143]. However, the exact mechanisms underlying the therapeutic effects of SCFAs in IBD require further elucidation, highlighting the complexity of their relationship with the disease. Figure 2 illustrates the mechanism of action of SCFAs.

#### 4.1.2. Colorectal Cancer

CRC ranks among the top three causes of cancer-related mortality worldwide, with increasing recognition of the microbiota’s contribution to its pathogenesis [144]. Various factors contribute to CRC, including a high-fat diet, stress, antibiotics, synthetic food additives, a sedentary lifestyle, and environmental factors [145]. A high-fat diet, especially prevalent in Western diets featuring high red and processed meat consumption, high fructose corn syrup, and unhealthy cooking methods, significantly contributes to CRC [146]. Current research has explored the protective role of dietary fibers in reducing the risk of CRC [147,148].

A systematic review and meta-analysis by Alvandi et al., explored the role of fecal SCFAs in CRC incidence and risk stratification [149]. The study, encompassing seventeen case-control and six cross-sectional studies, revealed that individuals with lower concentrations of acetic, propionic, and butyric acid are at a higher risk of CRC. Although these findings suggest a potential association between decreased fecal SCFAs concentrations and CRC susceptibility, emphasizing the importance of gut microbiota and bacterial metabolites in CRC prevention, their exact role in CRC prevention remains poorly understood. SCFAs, notably butyrate and propionate, are thought to influence CRC by regulating gene expression, expressing immunomodulatory effects, promoting immune cell differentiation, and mitigating inflammation. Moreover, compelling evidence underscores the role of SCFAs, including butyrate and propionate, in directly influencing intestinal epithelial cell transformation and inhibiting CRC by regulating tumor suppressor gene expression, promoting apoptosis, and modulating CRC cell proliferation and metabolism [150,151,152]. Butyrate is an energy metabolite and supports normal colon cell proliferation. In colorectal cancer (CRC) cells, butyrate alters cellular metabolism by boosting the activity of Pyruvate kinase muscle isozyme 2 (PKM2), suppressing the Warburg effect, and augmenting energy metabolism. Consequently, this impedes the proliferation of cancerous colonocytes, which depend on glucose as a result of the Warburg effect [120,153]. SCFAs function as inhibitors of HDACs, promoting apoptosis in cancer cells [151,154,155,156,157,158]. Additionally, SCFAs play a pivotal anti-inflammatory role in regulating local and systemic immune cells, contributing to their antitumor efficacy [159]. SCFAs mitigate inflammation by inhibiting nuclear factor kappa-light-chain-enhancer of activated B cells (NF-κB) activation, decreasing pro-inflammatory cytokine expression such as tumor necrosis factor-alpha (TNF-α), promoting anti-inflammatory cytokines such as IL-10, and transforming growth factor-beta, and facilitating the differentiation of naïve T cells into Tregs, thereby dampening immune responses [160]. They promote antimicrobial compound production, neutrophil and macrophage inhibition, Treg activation, and dendritic cell induction of tolerogenic properties [159]. In a recent in vitro experiment by Mowat et al., CRC cells treated with SCFAs induced much greater activation of CD8+ T cells than untreated CRC cells [160]. Surprisingly, the butyrate-producing bacterium *Fusobacterium nucleatum* does not consistently inhibit colon cancer; instead, it may promote cancer progression via mechanisms such as TLR4/myeloid differentiation primary response 88 (MYD88)/NF-κB signaling [161]. Furthermore, despite the anticipated decrease in DNA damage within cancer cells, numerous reports suggest that SCFAs might exacerbate DNA damage accumulation in CRC cells by disrupting DNA repair mechanisms [158,162,163,164,165]. Hence, the antitumorigenic effects of SCFAs likely involve intricate mechanisms extending beyond the tumor cells themselves. Such effects are particularly significant in CRC cells with underlying DNA repair defects, such as the microsatellite instability-high (MSI-h) CRC subset known for its heightened immunogenicity. Given inflammation’s potent role in tumor progression, these effects likely contribute to SCFAs’ antitumor efficacy. However, as tumor-targeted T-cell responses are crucial for antitumor immunity and treatment efficacy, SCFAs like butyrate may suppress such responses, potentially fueling tumor progression and compromising treatment outcomes [166,167,168,169].

Tian et al., investigated the potential protective role of SCFAs in the development of colitis-associated CRC using a mouse model induced by azoxymethane (AOM) and dextran sodium sulfate (DSS) [170]. The researchers administered a mix of SCFAs in the drinking water throughout the study. They found that the SCFAs mix significantly reduced tumor incidence and size in the mice with colitis-associated colorectal cancer. Additionally, the SCFAs mix improved colon inflammation and disease activity index score and suppressed the expression of proinflammatory cytokines such as IL-6, TNF-α, and IL-17. These findings suggest that SCFAs mix administration could prevent tumor development and attenuate colonic inflammation, indicating its potential as an agent for the prevention and treatment of colitis-associated colorectal cancer. Further investigations are warranted to determine if supplementing with dietary butyrate or consuming foods rich in butyrate-producing bacteria, such as omega-3 polyunsaturated fatty acids, can effectively hinder colorectal cancer and lower its occurrence.

#### 4.1.3. Disorders of the Gut-Brain Axis

The gut-brain axis facilitates bidirectional communication between the gastrointestinal and nervous systems through a complex signaling pathway network [171,172,173]. This intricate system encompasses connections such as the enteric nervous system, vagus nerve, immune system, endocrine signals, microbiota, and metabolites. Disruption of communication along the gut-brain axis is increasingly recognized as a significant contributor to neuroinflammation, which is considered a common feature of several neurodegenerative diseases, including Alzheimer’s and Parkinson’s diseases, characterized by chronic and debilitating conditions marked by the progressive degeneration of neurons [174,175,176,177,178,179,180]. Recent research suggests that neurodegenerative diseases may originate in the intestinal epithelium before affecting the brain via the gut-brain axis [181,182,183,184,185,186]. Numerous investigations have reported the buildup of protein aggregates, which are hallmark pathologies of neurodegenerative disorders like Alzheimer’s and Parkinson’s, in enteric neurons or the gastrointestinal epithelium long before they are observed in the central nervous system [179,187,188,189,190]. Functional studies highlight major microbiota components’ roles in the gut-brain axis [191,192,193,194]. An important aspect is the observed close correlation between alteration in the microbiota, mucosal immunity, and intestinal vascular impairment, potentially leading to the gradual release of systemic inflammatory mediators and bacterial components such as lipopolysaccharides (LPS), thereby initiating or exacerbating the development of neurological disorders [195,196,197]. Evidence suggests that microbial and systemic inflammatory molecules could contribute to cerebral vascular impairment, microglial activation, neuronal dysfunction, and pre- and post-synaptic activity imbalances. The microbiome of patients with Parkinson’s and/or Alzheimer’s disease exhibits a reduction in SCFAs-producing bacteria [195,198]. Recent research has highlighted their importance for learning and memory, with cuts in SCFAs associated with inflammation in Multiple Sclerosis patients and compromised neuronal function in various neurodegenerative diseases [199,200]. Furthermore, SCFAs appear to have neuroprotective roles, affecting the brain indirectly or directly by acting as ligands for GPCRs or as epigenetic modulators of HDAC to control transcriptional changes that affect neuronal functions [201,202,203,204,205]. The diminished concentration of SCFAs is suggested to be a critical factor in disrupting gut-brain balance, but the role of SCFAs in this context is under active investigation. These SCFAs can cross the blood-brain barrier, likely through the monocarboxylate transport system, influence brain function, and regulate blood flow, with dietary butyrate demonstrating an anti-inflammatory effect in the brain by influencing blood–brain barrier permeability [206,207]. SCFAs have also been implicated in maintaining gut and immune homeostasis in mammalian systems, highlighting their neuro-immunoendocrine regulatory role in the brain [206,208]. In Parkinson’s disease, the decline in butyrate levels is thought to lead to intestinal barrier integrity impairment, release of LPS and other pro-inflammatory molecules into the bloodstream, and triggering of microglial activation [122,209]. Furthermore, reduced SCFAs and microbiota alterations result in decreased circulating GLP-1 levels. The lowered SCFAs-mediated secretion of GLP-1 may activate pro-inflammatory pathways and depressive symptoms in PD patients [210,211]. Additionally, butyrate can induce epigenetic modifications in the genome of neurodegenerative disorder patients. Methylation analysis on blood samples from Parkinson’s disease patients and controls revealed a correlation between alterations in butyrate-producing bacterial taxa and epigenetic changes in genes containing butyrate-associated methylation sites. Notably, these modified sites coincide with genes implicated in psychiatric and gastrointestinal disorders [212].

In a study by Kong et al., 16S ribosomal RNA gene sequencing and gas chromatography-mass spectrometry analyses in a Drosophila model of Alzheimer’s disease revealed a decrease in *Lactobacillus* and *Acetobacter* species correlating with a dramatic reduction in acetate [213]. Similarly, in Drosophila models of Parkinson’s disease, administration of sodium butyrate reduced degeneration of dopaminergic neurons and improved locomotor defects in a pan-neuronal transgenic fly model expressing mutant-human-α-Synuclein [207]. The SCFAs composition derived from microbes also clinically correlates with neural activity and brain structure, as evidenced by functional and structural magnetic resonance imaging [214]. Recently, Muller et al., examined the fecal SCFAs profile of patients with a major depressive disorder/generalized anxiety disorder, comparing it with nuclear magnetic resonance spectroscopy and self-reported depressive and gut symptoms. The severity of depressive symptoms positively correlated with acetate levels and negatively correlated with butyrate levels [215]. In preclinical studies focusing on Alzheimer’s disease, prebiotic and probiotic supplementation appear advantageous, although limited data is available specifically on SCFAs. Bonfili et al., demonstrated the positive impacts of SLAB51 treatment, a mixture of lactic acid bacteria and bifidobacteria on eight-week-old transgenic Alzheimer’s disease model mice over four months [216,217,218]. SLAB51 administration enhanced performance in the novel object recognition test, reduced brain damage, decreased Aβ plaques, elevated SCFAs, and lowered plasma cytokine levels [218]. Additionally, prebiotics have shown efficacy in Alzheimer’s disease amyloid models. Liu et al., treated 5XFAD transgenic Alzheimer’s disease model mice with prebiotic mannan oligosaccharide for eight weeks starting from birth. The 5XFAD transgenic mouse was developed in 2006 and overexpresses human APP with three FAD mutations (the Swedish (K670N, M671L), Florida (I716V), and London (V7171) mutations) and human PSEN1 with two FAD mutations (M146L and L286V) [219]. They observed improvements in cognitive deficits, reduction in amyloid β (Aβ) plaques, decreased oxidative stress, diminished microglial activation, and alterations in the gut microbiome. Interestingly, gut microbiome-induced changes in the brain appeared to be mediated by SCFAs, as supplementation with SCFAs produced similar effects [220]. Finally, a case report demonstrated that fecal microbiota transplantation (FMT) improved cognitive function, microbiota diversity, and SCFAs production in an Alzheimer’s patient [221].

Several studies have investigated the administration of probiotics in both murine models and human subjects with Parkinson’s disease, exploring their impact on gastrointestinal and neurological symptoms [222,223,224,225,226,227,228]. A pilot study regarding FMT use in Parkinson’s patients has recently been published, with promising data [229]. However, only a few studies have evaluated the role of SCFAs. Specifically, *Bifidobacterium* has been demonstrated to be effective in modulating the host microbiota in a murine model induced by 1-methyl-4-phenyl-1,2,3,6-tetrahydropyridine (MPTP) [230]. In mice overexpressing α-synuclein, a prebiotic diet altered the activation of microglia and motor deficits by changing the composition of the gut microbiome and levels of SCFAs [231]. Combining polymannuronic acid with *Lacticaseibacillus rhamnosus* GG demonstrated more potent neuroprotective effects against Parkinson’s disease than either treatment alone, suggesting the therapeutic promise of synbiotics in Parkinson’s disease [232]. Oral administration of *Bifidobacterium breve* CCFM1067 to MPTP-induced Parkinson’s disease mice led to a reduction in intestinal microbial alterations, marked by a decline in pathogenic bacteria (*Escherichia-Shigella*) and an increase in *Bifidobacterium* and *Akkermansia*. This intervention also restored SCFAs production (butyrate and acetate), which may account for the observed local and cerebral anti-inflammatory effects. Recently, *Bifidobacterium animalis subsp. lactis* Probio-M8 (Probio-M8) was examined to evaluate its additional beneficial effects and mechanisms when used as an adjunct treatment alongside conventional therapy (benserazide and dopamine agonists) in patients with Parkinson’s. This investigation was conducted over three months in a randomized, double-blind, placebo-controlled trial [233]. Clinical outcomes were assessed by analyzing changes in various clinical indices, gut microbiome composition, and serum metabolome profiles before, during, and after the intervention. The findings revealed that co-administration of Probio-M8 resulted in additional benefits, including improved sleep quality, reduced anxiety, and alleviated gastrointestinal symptoms. Metagenomic analysis demonstrated significant modifications in the participants’ gut microbiome and serum metabolites following the intervention. The serum concentration of acetic acid was notably higher in the probiotic group.

IBS is a disorder of gut-brain interaction (DGBI) characterized by abdominal pain and changes in stool consistency or frequency. According to the Rome IV criteria, IBS can be divided into four subtypes based on the primary clinical features: IBS with diarrhea (IBS-D), IBS with constipation (IBS-C), IBS with mixed stool patterns (IBS-M), and unclassified IBS [234,235,236]. SCFAs play a pivotal role in IBS, with reported findings indicating that patients with IBS exhibited significantly elevated levels of acetate, propionate, and total SCFAs in fecal samples, with the severity of symptoms correlating positively [237]. Alterations in SCFAs levels are subtype-specific, with reduced levels in IBS-C and increased levels in IBS-D compared to controls [238,239]. Treem et al., sought to investigate whether patients with IBS-D exhibit a distinct pattern and pace of carbohydrate and fiber fermentation in SCFAs in in vitro studies of fecal homogenates compared to controls. The fecal SCFAs profile of IBS-D patients revealed diminished concentrations of total SCFAs, acetate, and propionate alongside elevated levels and proportion of n-butyrate [240]. Fredericks et al., in 2021, examined gut microbiota, concentrations of SCFAs, and mRNA expression of monocarboxylate transporters in individuals with IBS-C, IBS-D, and healthy controls. They observed changes in fecal SCFAs ratios in both IBS groups, with a decrease in all three measured SCFAs in IBS-C and a reduction specifically in acetic acid in IBS-D [241]. Similarly, Undseth et al., aimed to compare colonic fermentation between individuals with IBS and healthy counterparts by examining serum SCFAs concentrations before and 90 min after ingesting lactulose, an unabsorbable yet fermentable carbohydrate. They found that reduced serum SCFAs levels post-lactulose ingestion may indicate compromised colonic fermentation in IBS patients [242]. The dysregulated SCFAs levels in feces are linked to shifts in intestinal bacterial composition in IBS patients, characterized by higher amounts of acetate and propionate-producing bacteria like *Veillonella* and *Lactobacillus* and lower amounts of butyrate-producing bacteria like *Roseburia-Eubacterium* rectale group [237,243,244]. Zhou et al., recently set out to investigate how linaclotide affects the gut microbiota and pinpointed essential bacterial genera that could influence linaclotide’s effectiveness. Interestingly, they discovered a direct link between higher levels of *Blautia* and SCFAs concentrations and the amelioration of clinical symptoms in patients with IBS-C [245].

SCFAs, particularly propionate and butyrate, show promise as non-invasive biomarkers for diagnosing IBS, with diagnostic properties consistent across all IBS subgroups. Farup et al., 2016 examined fecal SCFAs as a potential diagnostic indicator for IBS in a study involving 25 IBS subjects and 25 controls. They assessed total SCFAs levels and individual SCFA amounts to identify the most effective diagnostic approach. Their findings revealed that the discrepancy between propionic and butyric acid levels demonstrated superior diagnostic performance using a threshold of 0.015 mmol/l to indicate IBS, independent of the IBS subgroup [246].

Several potential mechanisms exist through which SCFAs could influence the pathophysiology of IBS, many of which have been previously examined in the IBD section of this review. As already described, SCFAs interact with specific receptors, such as GPR41, GPR43, and GPR109A, expressed in various gastrointestinal cell types, modulating physiological responses. They play a multifaceted role in immunity and inflammation, influencing inflammatory mediator production, immune cell differentiation, and intestinal barrier integrity [247,248,249,250,251]. Additionally, SCFAs influence the differentiation of immune cells, including T cells and Tregs, and suppress intestinal inflammation [131,252]. They also contribute to the integrity of the intestinal barrier by promoting mucin secretion and enhancing tight junction assembly [253,254,255,256,257,258].

Furthermore, SCFAs impact gut motility through various mechanisms, including modulation of neural activity, neurotransmitter release, and regulation of calcium signaling and smooth muscle contractility [259,260,261,262,263,264,265,266,267,268,269,270]. The effects of SCFAs on colonic motility are nuanced and context-dependent, varying based on SCFAs concentration and colonic segment [259,260,261,262,263,264,265,266,267,268,269,270]. Waseem et al., in their recent prospective observational study, investigated the associations between fecal SCFAs, colonic transit time, fecal bile acids, and dietary intake in individuals with IBS and healthy controls [271]. They found that fecal SCFAs were inversely correlated with overall and segmental colonic transit time, with similar patterns observed in both IBS and healthy control groups. Additionally, the acetate-to-butyrate ratio was associated with slower transit times. Logistic regression analyses demonstrated that acetate could accurately predict delayed colonic transit time and bile acid diarrhea (BAD). These findings suggest that fecal SCFAs and dietary factors may play a role in the IBS pathophysiology and serve as diagnostic markers for bowel transit disorders [271].

### 4.2. Metabolic Diseases

Metabolic disorders, including obesity, type 2 diabetes (T2D), and metabolic dysfunction-associated steatotic liver disease (MASLD), present significant health challenges globally [272,273,274]. Pivotal to the pathophysiology of these conditions is the intricate interplay between the gut microbiota and SCFAs, which profoundly influence host metabolism. An imbalance in gut microbial communities is a critical contributor to the development of common metabolic disorders in humans [275]. Nevertheless, the emerging evidence underscores the promising therapeutic potential of targeting the gut microbiota and its metabolites for managing various metabolic conditions, extending beyond the well-established associations with obesity, T2D, and MASLD. Mechanisms of SCFAs in metabolic disorders are summarized in Table 7.

#### 4.2.1. Obesity

Obesity poses a significant risk for various chronic conditions, including T2D, insulin resistance, MASLD, and cardiovascular disease, among others [276]. Interestingly, obese individuals have been associated with altered fecal SCFAs concentrations, particularly propionate. A study involving Mexican children revealed that those with excess weight and obesity exhibited lower concentrations of fecal propionate and butyrate compared to their normal-weight counterparts [277]. A recently published study examined African-origin groups from different regions and discovered variations in gut microbiota composition and predicted functions linked to population obesity and geography [278]. The study found that fecal SCFAs concentrations are inversely correlated with microbial diversity and obesity. However, the prediction of obesity from microbiota varied by country: *Prevotella*-rich microbiota dominates in traditionally non-western groups, while Bacteroides-rich microbiota is found in high-income countries. Conversely, other studies have associated obese individuals with higher fecal SCFAs concentrations than lean individuals [279,280]. A study in the Netherlands found that overweight and obese individuals had elevated fecal SCFAs concentrations compared to lean counterparts, suggesting enhanced microbial energy extraction [279]. Indeed, a previous survey of 441 adults published by Cuesta-Zuluaga et al., in 2018 revealed a correlation between higher fecal SCFAs levels and obesity [281]. The excessive production of SCFAs may contribute to weight gain due to increased energy storage despite its typically beneficial effects on well-being [279,282,283,284,285].

However, these findings are debatable due to possible fluctuations in SCFAs concentrations and broader microbiota alterations within the intestinal microbial community [286]. Numerous studies have investigated the role of SCFAs in adiposity, examining human subjects and conducting in vitro and in vivo animal studies. In vitro studies have demonstrated that acetate and propionate treatment can induce expressions of vital metabolic regulators, promoting lipolysis metabolism [287,288].

Animal studies have shown that SCFAs supplementation can counteract weight and adiposity gain, with treatments like sodium butyrate inducing weight loss by enhancing energy expenditure and fat oxidation [289,290]. In mice on a high-fat diet, butyrate supplementation increases the expression of peroxisome proliferator-activated receptor-γ (PPARγ) coactivator-1 alpha (PGC-1α), activates 5’ adenosine monophosphate-activated protein kinase (AMPK) and p38, and improves insulin sensitivity, thus inducing weight loss by enhancing energy expenditure and fat oxidation [291]. This finding was observed when the functioning of adipose and hepatic PPARγ pathways were intact. Dietary supplementation with SCFAs has been found to upregulate GPR43 and GPR41 expressions in adipose tissue, enhance triglyceride hydrolysis, promote free fatty acid oxidation in adipose tissue leading to brown fat production, and reduce body weight in high-fat diet (HFD)-fed mouse models [292]. *Ganoderma lucidum*, a medicinal mushroom with a long history of use in Asian countries, has been shown to increase SCFAs production and GPR43 expression in C57BL/6 J mice, enhance ileal tight junction proteins and antibacterial peptides expression, mitigate endotoxemia, and attenuate HFD-induced upregulation of TLR4/Myd88/NF-κB signaling in adipose tissue [293,294].

Overall, while growing evidence supports the role of SCFAs in obesity treatment, comprehensive mechanistic studies are needed to elucidate their precise mechanisms of action and optimize their therapeutic potential.

#### 4.2.2. Type 2 Diabetes

Research involving individuals from various ethnic backgrounds has revealed that those with T2D exhibit diminished levels of SCFAs-producing bacteria. This is implicated in insulin resistance and the progression of T2D and can contribute to gut inflammation [295].

Regarding microbial metabolites, SCFAs exhibit diverse effects across various sites regulating glucose metabolism. In vitro and in vivo studies have shown that SCFAs act as potent secretagogues for glucagon-like peptide 1 (GLP-1) and peptide YY (PYY), thereby enhancing feelings of satiety via the gut-brain axis. Consecutively, they may indirectly decrease appetite and subsequent food intake, thus mitigating the risk of weight gain, a known predisposing factor for T2D [203]. Research has revealed that acetate can reduce hormone-sensitive lipase phosphorylation in human multipotent adipose tissue-derived stem adipocytes in a Gi-coupled manner [296]. Acetate and butyrate activate GPR43 and GPR41 on rat intestinal cells, stimulating insulin, GLP-1, and peptide YY secretion, modulating blood lipid metabolism and lowering peripheral blood glucose levels, slowing intestinal transit, decreasing gastric emptying, food intake, and intestinal motility [297]. Acetate and butyrate activate GPR43 and GPR41 receptors on intestinal cells, promoting the secretion of insulin, GLP-1, and peptide YY, which helps modulate blood lipid metabolism and lower peripheral blood glucose levels [298,299]. In the liver, SCFAs have been observed to inhibit glycolysis and gluconeogenesis while enhancing glycogen synthesis and fatty acid oxidation [203,300,301,302]. Additionally, SCFAs have been shown to improve glucose uptake in skeletal muscle and adipose tissue by upregulating the expression of insulin-responsive glucose transporter type 4 (GLUT4) through AMPK activation. Furthermore, in skeletal muscle, SCFAs reduce glycolysis, leading to the accumulation of glucose-6-phosphate and increased glycogen synthesis [291,300,301,302,303,304,305]. In preclinical models, ingesting soluble dietary fibers prompts the production of SCFAs, particularly propionate, and butyrate, which activate intestinal gluconeogenesis (IGN), a process crucial for glucose and energy homeostasis [306]. SCFAs play a role in promoting IGN production and mitigate metabolic diseases in mice [307]. Butyrate triggers IGN gene expression via a cAMP-dependent mechanism. At the same time, propionate, as an IGN substrate, enhances gene expression through activation of the gut-brain neural circuit [306], thereby exerting beneficial effects on glucose regulation, energy balance, and body weight control. In rabbits, acetate could curb lipid accumulation, promoting lipolysis and fatty acid oxidation and inhibiting synthesis [308].

Regarding the microbiota populations, T2D patients exhibit a higher abundance of *Proteobacteria* and a modified *Firmicutes/Bacteroidetes* ratio compared to healthy individuals, alongside reduced SCFAs-producing *Bacteroides* [309,310,311]. Acetate and butyrate improved intestinal barrier function and increased the number of *Bacteroidetes* spp. in nonobese diabetic (NOD) model mice, which helped to inhibit T1D [312].

As a result of the role of SCFAs in human glucose metabolism, intervention studies involving the supplementation of propionate and butyrate have been conducted. A recent meta-analysis has shown that probiotic intervention can significantly improve the homeostatic model assessment of insulin resistance (HOMA-IR) and considerably decrease glycated hemoglobin HbA1c levels and fasting blood glucose levels in T2DM patients compared to placebo [313,314]. However, the evidence remains inconclusive due to the limited number of studies conducted in small cohorts. Nevertheless, these studies suggest that inulin-propionate supplementation (10 g/day) increases GLP-1 and PYY levels while reducing food intake, and therefore contributing to body weight regulation [315]. Additionally, sodium butyrate supplementation (4 g/day) enhances insulin sensitivity solely in lean individuals and not in those with metabolic syndrome [316]. Despite these promising findings, the optimal doses and exposure durations for SCFAs treatment in T2D remain undefined, and further research is needed to elucidate their time- and dose-dependent effects. Additionally, studies have focused on translating fecal microbiota from lean donors to recipients with metabolic syndrome to enhance insulin sensitivity [317,318].

Moreover, adopting a low-calorie, low-protein, low-carbohydrate HFD as a fast-mimicking diet has shown promise in promoting cell regeneration, reducing protein kinase A and mammalian target of rapamycin activity, inducing the expression of SRY (sex determining region Y)-box 2 (Sox2) and neurogenin-3 (Ngn3), and restoring insulin production, secretion, and glucose homeostasis in both T2D mouse models and type 1 diabetes patients [319].

#### 4.2.3. Metabolic Dysfunction–Associated Steatotic Liver Disease

The transition from non-alcoholic fatty liver disease (NAFLD) to metabolic-associated fatty liver disease (MAFLD) and MASLD marks a significant shift in the understanding and classification of metabolic liver diseases, aiming to better reflect their pathophysiology and reduce social stigma [274]. This evolution in terminology and diagnostic criteria, supported by international experts and widely accepted in clinical practice guidelines, emphasizes the link between metabolic dysfunction and liver health, paving the way for improved disease identification and management strategies. The connection between MASLD and its advancement to steatohepatitis and cirrhosis has previously been associated with the gut microbiome via multiple pathways. This correlation could stem from gut microbiota alterations and the systemic impact of metabolites derived from it, such as SCFAs [320].

Notably, the gut microbiota of patients with the formerly known NAFLD exhibits a significantly reduced abundance of SCFAs-producing bacteria such as *Bacteroides*, *Lactobacillus curvatus*, and *Lactobacillus plantarum* [321,322,323,324]. As described in this review, previous studies have suggested that individuals with obesity and MASLD tend to have higher levels of fecal SCFAs [279,281,325]. However, it is unclear whether there is a relationship between circulating SCFAs levels and MASLD and other metabolic disorders [283,326,327,328]. While some studies have found no significant differences between control groups and MASLD patients, others have reported lower SCFAs levels in MASLD cirrhosis or higher levels in patients with hepatocellular carcinoma and cirrhosis linked to MASLD [326,328,329,330]. These conflicting conclusions may result from differences in study design, such as variations in the selection criteria for control and MASLD patients or discrepancies in the severity of underlying MASLD conditions.

The mechanisms linking SCFAs and MASLD may involve alterations in glucose homeostasis, lipid metabolism, and inflammatory and immune responses [325,331]. The gut-liver axis plays a crucial role in this process, as emphasized by the reciprocal relationship between gut microbiota, gut-derived metabolites, and liver function [332].

Although the precise role of these SCFAs in MASLD remains unclear, insights may be gleaned from research on other metabolic disorders as previously described in this review. Previous studies have associated acetate with greater gut microbiota diversity, reduced visceral fat, and less severe MASLD cases [333,334]. Consistent with these findings, our study observed lower acetate levels in MASLD patients than in healthy controls. Propionate, when present in adequate concentrations, is also linked to positive health outcomes and the regulation of gut hormones influencing appetite and fullness [334]. However, conflicting evidence exists, as emphasized by a study on early MASLD patients where higher levels of SCFAs-producing bacteria and fecal acetate and propionate were associated with an elevated TH17/Treg ratio, suggesting a potential contribution to low-grade inflammation [325].

In a recent study, Thing et al., investigated the association between plasma SCFAs and MASLD. The results showed higher plasma concentrations of propionate, formate, valerate, and α-methylbutyrate but lower plasma acetate concentrations in MASLD patients compared to healthy controls. Moreover, among MASLD patients, significant fibrosis was positively associated with several SCFAs [335].

Animal studies have shown that supplementation with SCFAs such as sodium acetate and sodium butyrate can protect against hepatic steatosis induced by nicotine and metabolic factors [336,337]. In MASLD patients, downregulation of the GLP-1 receptor in the liver is observed, with butyrate supplementation in MASLD mice enhancing GLP-1 receptor expression by inhibiting HDAC-2, consequently promoting energy metabolism and inhibiting lipid accumulation [338]. Butyrate also improves insulin sensitivity, activates AMPK to induce the expression of fatty acid oxidation genes in hepatocytes, and reduces fat deposition in MASLD mice [339]. The MASLD mouse model increases the abundance of beneficial bacteria in the intestine, such as *Christensenellaceae*, *Blautia*, and *Lactobacillus*, establishing a positive feedback loop by augmenting butyric acid production [340,341]. Additionally, butyrate attenuates MASLD-induced intestinal mucosal injury by upregulating zonula occludens-1 (ZO-1) expression in the intestinal tract of mice, thereby preventing enterotoxin migration to the liver and suppressing liver inflammation [342].

Overall, these findings underscored the therapeutic potential of SCFAs in preventing and managing MASLD by targeting multiple pathways involved in its pathogenesis. Emerging evidence underscores the pathogenic role of microbe-derived metabolites, including trimethylamine, secondary bile acids, SCFAs, and ethanol, in MASLD pathogenesis [332].

### 4.3. Therapeutic Implications

#### 4.3.1. Fecal Microbiota Transplantation

FMT is a therapeutic approach involving the transfer of a fecal suspension from a healthy donor to the patient’s gastrointestinal tract to restore average microbial composition and function [343,344]. It is recommended by guidelines and consensus from international societies for the treatment of recurrent *Clostridioides difficile* infection (rCDI) [345,346,347,348,349]. Encouraging results indicate that FMT might also potentially treat additional conditions linked to disruptions in gut microbiota composition, including IBD and disturbances of the gut-brain axis, like anorexia [343,345,350,351,352,353,354,355,356]. The efficacy of FMT largely depends on the donor’s microbiota, with “super donors” possessing favorable bacterial characteristics crucial for successful outcomes [357]. Advancements in frozen stool processing have facilitated the establishment of FMT libraries for clinical applications [353,358]. However, the specific bacterial composition of FMTs and the underlying treatment mechanisms remain unclear, necessitating further research to better understand this promising therapeutic approach [359].

Metabolite levels linked to gut microbiota, including SCFAs and bile acids, show improvement following FMT. Paramsothy et al., found that patients with UC achieving remission after FMT exhibited enrichment of *Eubacterium hallii* and *Roseburia inulinivorans*, along with elevated levels of SCFAs biosynthesis and secondary bile acids, compared to non-responders [360]. FMT administration is thought to elevate SCFAs levels in the colon and regulate the NF-κB pathway to reduce inflammation [361,362]. In a study conducted by Osaki et al., in 2021, the effectiveness of FMT was evaluated along with its impact on fecal microbiota and SCFAs levels in patients with IBD and rCDI. The analysis of fecal microbiota showed changes in bacterial composition after FMT, with modifications in specific bacterial taxa associated with clinical response. In UC patients, fecal SCFAs levels remained unchanged post-FMT, regardless of treatment response. However, responders showed a significant increase in fecal butyric acid levels in CD patients at eight weeks post-FMT compared to donors, while rCDI patients had lower pre-FMT butyric acid levels than donors. Furthermore, fecal propionic acid levels significantly increased at eight weeks post-FMT in rCDI patients, while acetic acid and butyric acid levels showed a non-significant increase [363]. Conversely, Seekatz et al., observed increased butyrate, acetate, and propionate levels and recovery of secondary bile acids like deoxycholate and lithocholic acid in rCDI patients post-FMT [364].

A 2021 RCT conducted by El-Salhy and colleagues investigated the impact of FMT on fecal SCFAs levels in patients with IBS. The study included 142 participants from a previous study. The results showed that individuals who received FMT had increased levels of butyric acid, especially in the 30-g and 60-g FMT groups. In addition, the 60-g FMT group had higher levels of total SCFAs and several other SCFAs types. Significantly, higher butyric acid levels were associated with symptom improvement in FMT responders [365].

#### 4.3.2. Dietary Intervention

Dietary composition exerts a significant influence on gut microbes [366,367]. Various diets can alter microbial composition, increase the ratio of harmful bacteria to beneficial metabolites, and contribute to the development of chronic metabolic diseases such as obesity and T2D [368,369]. The potential role of dietary interventions in diseases from cognitive impairment to IBD has inspired new studies on the connection between diet and microbiota [370,371,372]. Adopting healthy eating habits with a diet rich in fresh fruits, vegetables, and whole grains can reduce the risk of cardiovascular and metabolic diseases and cancer. On the other hand, consuming refined and processed foods such as sugary treats, fried foods, processed meats, and refined grains may increase their likelihood [369,373].

Dietary fiber is an essential component of food, and soluble fiber is resistant to gastrointestinal digestive enzymes and is utilized by the anaerobic intestinal microbiota to produce SCFAs [374]. In a recent systematic review examining the impact of dietary fibers on SCFAs production and gut microbiota composition in healthy adults, a total of forty-four human intervention studies on confirmed and candidate prebiotics were included. Among them, inulin was the most extensively studied dietary fiber. While specific studies indicated notable rises in total SCFAs after dietary fiber intervention, others observed no significant alterations, indicating that the influence of nutritional fibers on SCFAs levels may be affected by variables such as dosage, fiber type, and baseline gut microbiota composition [375,376]. To analyze the potential mechanisms of the role of the ketogenic diet in epilepsy, a recent study by Gudan et al., examined the impact of this on the synthesis of intestinal SCFAs in healthy adults [377]. The analysis highlighted that cruciferous and leaf vegetables, berries, and nuts consumption on a ketogenic diet have been linked to a positive impact on the profile of SCFAs. The LIBRE trial (Lifestyle Intervention Study in Women with Hereditary Breast and Ovarian Cancer) investigated the effect of the Mediterranean diet in 260 women and found that adherence to the Mediterranean diet led to increased fecal SCFAs levels, particularly propionate and butyrate [378].

Dietary fibers play a crucial role in modulating intestinal SCFAs levels, preserving mucosal homeostasis, enhancing intestinal epithelial integrity, fostering the growth of Tregs, and suppressing the expression of inflammatory cytokines to prevent or alleviate disease [379]. Supplementation with wheat bran, rich in arabinoxylan oligosaccharides, elevated butyrate, acetate, and propionate levels, along with total SCFAs concentrations in a human trial [380]. However, the increased fecal bulking and reduced transit time associated with higher dietary fiber intake could decrease colonic SCFAs absorption, potentially explaining the observed rise in fecal SCFAs concentrations in studies with increased fiber content.

According to two studies, consumption of barley-kernel-based bread rich in β-glucan fibers for three days can result in an increase in the levels of *Prevotella* and a decrease in the levels of *Bacteroides* and intensified fermentation activity, SCFAs serum levels, and gut hormone secretion (GLP-1, PYY, and GLP-2) in healthy adults, enhancing insulin sensitivity [381,382]. These results were observed among healthy participants, and they suggest that certain foods can have a significant impact on the gut microbiome. This shift was linked to a decrease in postprandial glucose response, corresponding to an increase in total serum SCFAs concentration. Another study indicated that a supplement containing three grams per day of high molecular weight β-glucan altered the gut microbiota composition, increasing *Bacteroidetes* and decreasing *Firmicutes*, with correlations observed between changes in these bacteria and cardiovascular disease risk factors [383]. These findings suggest that high molecular weight β-glucan fibers can induce microbiota shifts, potentially explaining their metabolic benefits.

In 2020, Farup and Valeur conducted a study to investigate the impact of weight-loss interventions on fecal SCFAs levels in people with obesity. They studied ninety subjects with morbid obesity and measured their fecal SCFAs levels before and after a six-month conservative weight-loss intervention followed by bariatric surgery. The study found a reduction in total fecal SCFAs levels post-surgery, accompanied by a decrease in the main straight-chain SCFAs such as acetic-, propionic-, and butyric-acids, and an increase in branched-chain SCFAs like isobutyric-, isovaleric-, and isocaproic-acids. This indicated a shift towards a proteolytic fermentation pattern. Interestingly, SCFAs levels were associated with diet but not metabolic markers or fecal microbiota composition. This suggests that dietary interventions can potentially mitigate these effects [384].

#### 4.3.3. Prebiotic and Probiotic Applications

In recent years, there has been a surge of interest in prebiotics and probiotics [385], with their mechanisms of action being intricate and diverse, often specific to particular strains and compounds [386]. Probiotics can alter the gastrointestinal microenvironment, outcompete pathogenic bacteria for nutrients, and hinder pathogenic growth by producing antimicrobial compounds unique to each strain [387,388]. Probiotics’ safety and potential roles in diseases where gut microbiota is considered part of the pathophysiology have fueled research in this area [389,390]. SCFAs have the potential to regulate cognitive abilities and influence mental function via the gut-brain axis [391].

In 2015, Sawin et al., investigated the prebiotic properties of glycomacropeptide (GMP), a glycophosphopeptide. Using mouse models, researchers found that GMP reduced the abundance of *Desulfovibrio* bacteria, increased levels of cecal SCFAs, and exhibited anti-inflammatory effects compared to casein and amino acid diets [392].

Holmes et al., conducted a six-week, three-period prebiotic intervention study on forty-one healthy adults to analyze personalized responses to different prebiotics, inulin, galactooligosaccharides, and dextrin. They found that the proportional increase in butyrogenic response to prebiotics was inversely correlated with regular dietary fiber intake [393]. The study suggested that individuals’ gut microbiota may have a limited capacity to produce SCFAs from fiber, and their responsiveness to prebiotic treatment could be predicted based on diet and baseline SCFAs levels in the stool.

A systematic review and meta-analysis focusing on dietary fiber interventions in individuals with type 2 diabetes revealed improvements in the relative abundance of *Bifidobacterium* and total SCFAs. The interventions enhanced glycated hemoglobin levels [394]. This review included an intervention study involving 16 g per day of inulin-type fructans for six weeks, notably increasing *Bifidobacteria* concentrations [395]. Although the prebiotic treatment boosted fecal SCFAs concentrations, including total SCFAs, acetate, and propionate, it had no discernible impact on butyrate or overall bacterial diversity. Moreover, it did not positively influence glucose levels, insulin, gut hormones, appetite, or energy intake [396,397].

Inulin-type fructans possess a prebiotic effect, elevating *Bifidobacterium*, *Lactobacillus*, and *Faecalibacterium prausnitzii* abundances. The benefits reported include improved intestinal barrier function, insulin sensitivity, lipid profile, mineral absorption, and satiety [397]. However, the effects on blood glucose, cholesterol, and triglyceride concentrations appear favorable primarily in individuals with prediabetes and diabetes [398].

In a randomized, double-blind, placebo-controlled study assessing the impact of the probiotic intervention on fecal SCFAs, a multi-strain probiotic formula was administered to 56 postmenopausal obese women [399]. The study revealed a positive effect on their cardiometabolic health, with the higher probiotic dose group showing elevated levels of fecal SCFAs [399]. Another recent study investigated the impact of a low-carbohydrate diet compared to a habitual diet on fecal SCFAs levels and serum inflammatory markers in obese women undergoing an energy-restricted diet [400]. After adjusting for baseline parameters, the two diet groups observed significant differences in fecal levels of butyric, propionic, and acetic acid.

## 5. Conclusions

In conclusion, the absorption of short-chain fatty acids, notably butyrate, is pivotal for maintaining gastrointestinal health and addressing associated diseases. While passive diffusion was once thought to be the primary absorption mechanism for butyrate, recent findings reveal a more intricate process involving specific transport proteins such as monocarboxylate transporters (MCT1 and MCT4) and the sodium-coupled transporter (SMCT1). Dysregulation of these transporters has been implicated in various gastrointestinal disorders, including inflammatory bowel disease (IBD) and colorectal cancer (CRC). Furthermore, butyrate supplementation, whether in the form of calcium butyrate or sodium butyrate, shows promise in therapeutic interventions, particularly in cancer management. We have also observed that the local concentration of butyrate can be crucial in achieving a valid therapeutic effect. Indeed, recent clinical studies proposing reduced oral concentrations of butyrate have overall shown a better outcome compared to earlier studies utilizing enemas.

Additionally, propionate and acetate, two other prominent SCFAs, exert notable effects on gastrointestinal health, with propionate demonstrating potential in weight management and inflammation regulation. However, the existing literature highlights a significant disparity in clinical studies conducted on butyrate compared to propionate and acetate, with a lag in the availability of supplements based on propionate and acetate. This underscores the need for further exploration in this domain.

Finally, we highlighted how the role of SCFAs can be crucial in modulating the intestinal microbiota. Supplementation with SCFAs, but especially a diet rich in fiber and resistant starch, can facilitate the modulation and maintenance of a rich and diverse microbiota and a healthy intestinal barrier. An additional emerging idea is that any therapeutic, pharmacological, integrative, or nutritional intervention must consider the role played by the intestinal microbiota. For this reason, recognizing the importance of focusing on the microbiome leads to a “before” and “after” in health research and innovation, with the perspective nowadays being to develop personalized medicine for patients.

While preclinical and initial clinical studies are promising, more extensive clinical investigations are warranted to fully unravel the therapeutic potential of SCFAs in various gastrointestinal and metabolic conditions. Nevertheless, the excellent safety profile of SCFAs supplements augurs well for their future utilization in clinical settings.

## Figures and Tables

**Table 1:** Production of the three different SCFAs from different pathways and by other intestinal bacteria.

SCFAs	Metabolic Pathway	Bacteria Involved in the Production
Acetate	Acetogenesis	*Acetobacterium*, *Acetoanaerobium*, *Acetogenium*, *Butyrbacterium*, *Clostridium*, *Eubacterium*, *Pelobacter*
	Carbon fixation	*Bacteroides succinogenes*, *Clostridium butyricum*, *Syntrophomonas* sp.
Propionate	Succinate	Firmicutes *(Negativicutes*) and *Bacteroidetes*
	Propanediol	Lachnospiraceae (*Roseburia inulinivorans*, *Balutia* sp.)
Butyrate	Butyryl-CoA: acetate-CoA transferase	*Eubacterium*, *Roseburia*, *Anaerostipes*, *Faecalibacterium prausnitzii*
	Butyrate kinase	*Coprococcus* and *Clostridium* specific spp.

**Table 2:** Effects of butyrate interventions in IBD and non-IBD conditions. Abbreviations: s = significative improvement, nr = Information not reported, std = Standard therapy, ns = Not significative improvement, ps = Partial significative effect, DC = Diversion Colitis, DB = Double Blind, SB = Single Blind, UC = Ulcerative Colitis, CD = Crohn’s disease, A-S = Mesalamine + Sulfasalazine, CRP = Chronic Radiation Proctitis, ARP = Acute Radiation Proctitis, DM = Diabetes mellitus, DT1 = Type 1 diabetes, DT2 = Type 2 diabetes, Ob ped = Obese pediatrics, COPD = Chronic obstructive pulmonary disease, TD = Travelers’ Diarrhea, RCT = Randomized Clinical Trial, TB-RCT = Triple blind-RCT, QB-RCT = quadruple blind–RCT, not impr. = not improvement.

Ref.	Delivery	Year	Groups (*n*)	Design	Duration	Dosage Butyrate	Drugs (ad)	Improvement
[66]	enema	1991	DC (13)	DB	2 w	40 mmol/L	nr	ns
[67]	enema	1992	UC (10)	SB crossover	2 w	100 mmol/L	A-S	s
[68]	enema	1994	UC (10)	open label	6 w	80 mmol/L	A-S	60%
[69]	enema	1995	UC (40)	DB-RCT	6 w	200 mL/d mix	A-S	s
[70]	enema	1996	UC (38)	RCT	6 w	80 mmol/d	A/S	not impr.
[71]	enema	1996	UC (47)	DB-RCT	6 w	80 mmol/d	nr	not impr.
[72]	enema	1999	CRP (17)	DB-RCT	5 w	80 mmol/d	nr	s
[73]	enema	2000	UC (30)	RCT	6 w	4 gr/d	A	ns
[74]	enema	2000	APR (20)	RCT crossover	3 w	80 mmol/L	nr	s
[75]	enema	2002	UC (11)	RCT	8 w	100 mM	A/S/steroid	s
[76]	enema	2003	UC (51)	DB-RCT	6 w	80 mmol/L	M/steroid	S
[77]	oral	2005	CD (13)	open label	8 w	4 gr/d	A/S	69%
[78]	oral	2008	UC (216)	open label	24 w	921 mg/d	A + S	82.4%
[79]	enema	2009	IBS (11)	DB-RCT	1 w	50/100 mmol/L/d	nr	s
[80]	enema	2010	UC (35)	DB-RCT crossover	20 d	100 mmol/d	nr	s
[81]	oral	2013	IBS (66)	RCT	12 w	300 mg/d	std	s
[82]	oral	2014	DC (63)	RCT	12 month	300 mg/d	nr	s
[83]	oral	2014	TD (42)	RCT	3 d + trip	1500 mg/d	various	s
[84]	enema	2014	APR (166)	RCT	3 w	1–2–4 gr/d	nr	ns
[85]	enema	2016	Mix (20)	DB-RCT	4 w	600 mmol/L	nr	s
[86]	oral	2017	DM (40)	DB-RCT	45 d	600 mg/d	+inulin	s(+ inulin)
[87]	oral	2020	UC (39)	Prospective	12 months	1 g/d	std	s
[64]	oral	2020	IBD (49)	DB-RCT	8 w	600 mg/d	std	ps
[88]	oral	2020	DT1 (30)	DB-RCT	4 w	4 g/d	nr	ns
[89]	oral	2022	Ob ped (54)	QB-RCT	13 months	20 mg/kg	std	s
[90]	oral	2022	IBD ped (80)	RCT	12 w	150 mg/d	std	ns
[91]	oral	2022	DT2 (42)	TB-RCT	6 w	600 mg/d	nr	s
[92]	oral	2024	COPD (121)	RCT	12 w	300 mg/d	nr	s

**Table 3:** Effects of propionate interventions. Abbreviations: s = significative improvement, ACVD = Atherosclerotic cardiovascular disease, HovF = Healthy overweight females, MS = multiple sclerosis, IPE = inulin-propionate ester, DB = Double Blind, NaP = sodium-propionate, RCT = Randomized Clinical Trial.

Ref.	Delivery	Year	Groups (*n*)	Design	Duration	Dosage Propionate	Formula	Improvement
[102]	oral	2015	Obese (60)	DB-RCT	24 w	10 g/d	IPE	s
[103]	oral	2019	Obese (12)	DB-RCT cross over	42 d	20 g/d	IPE	s
[104]	oral	2019	HovF (20)	RCT	4 w	10 g/d	IPE	s
[101]	oral	2020	MS (36)/Healthy (68)	proof-of-concept	2 w	1 g/d	NaP	s
[105]	oral	2022	ACVD (62)	DB-RCT	8 w	1 g/d	propionic acid	s

**Table 4:** Currently recruiting and upcoming clinical trials examining the effects of propionate.

Name of Trial	Type	Identifier/Status	Condition	Intervention	Location
Combination of Medium Cut-off Dialyzer Membrane and Diet Modification to Alleviate Residual Uremic Syndrome of Dialysis Patients	RCT	*NCT04247867*/*recruiting*	Uremic syndrome	Psyllium-inulin/sodium propionate	University medical Centre Ljubljana, Ljubljana, Slovenia
The Effect of Combining Medium Cut-Off Dialysis Membrane and Diet Modification on Reducing Inflammation Response	RCT	*NCT04260412*/*recruiting*	Uremic syndrome	Psyllium-inulin/sodium propionate	University medical Centre Ljubljana, Ljubljana, Slovenia

**Table 5:** Effects of acetate interventions. Abbreviations: HinsF = Hyperinsulinemic females.

Ref.	Delivery	Year	Groups (*n*)	Design	Duration	Dosage Propionate	Formula	Improvement
[111]	Rectally and intravenous	2010	HinsF (6)	open label	4 times	60 mmol/L rectal + 20 mmon/Lintravenous	NaAcetate	s
[112]	Intravenous	2012	Overweight normoglycemic and hyperglycemic subjects (9)	open label	90	140 mmol/L	NaAcetate	no
[113]	Proximal and distal colonic	2016	Obese (6)	DB-RCT crossover	3 d	100–180 mmol/L	Acetate	s
[114]	Colonic infusions	2017	Obese (12)	DB-RCT crossover	4 d	200 mmol/L mix	(acetate, propionate, and butyrate)	s

**Table 6:** Mechanisms of SCFAs in gastrointestinal diseases. Abbreviations: SCFAs = Short-Chain Fatty Acids, NF-κB = Nuclear Factor kappa B, CRC = Colorectal Cancer, Tregs = Regulatory T cells, GPR43 = G-Protein-Coupled Receptor 43, GPR109A = G-Protein-Coupled Receptor 109.

Disease	Supposed Mechanisms of SCFAs Protection/Risk
Inflammatory Bowel Disease	1. Anti-inflammatory effects: Butyrate, a primary energy source for colonocytes, inhibits NF-κB activation, reducing proinflammatory gene expression.
	2. Maintenance of gut barrier integrity: SCFAs promote mucus production and tighten epithelial cell junctions, enhancing the intestinal epithelial barrier.
	3. Modulation of immune responses: SCFAs influence the differentiation and function of regulatory T cells (Tregs), suppressing excessive immune reactions. They engage with receptors like GPR43 and GPR109A to stimulate Treg production.
	4. Tissue repair and healing: SCFAs promote the proliferation and differentiation of epithelial cells, facilitating tissue repair processes within the gut damaged by inflammation in IBD.
Colon Cancer	1. Protective effects against CRC development: SCFAs exert protective effects against colorectal cancer by regulating gene expression, promoting apoptosis, and inhibiting CRC cell proliferation and metabolism.
	2. Anti-inflammatory actions: SCFAs mitigate inflammation in CRC by inhibiting NF-κB activation, decreasing pro-inflammatory cytokine expression, and promoting anti-inflammatory cytokines and regulatory T-cell differentiation.
	3. Potential DNA damage modulation: While SCFAs are anticipated to decrease DNA damage in CRC cells, reports suggest they may exacerbate DNA damage accumulation in some instances, possibly due to disruptions in DNA repair mechanisms. Further evidence is needed.
Disorders of the Gut-Brain Axis	1. Neuroprotective effects: SCFAs exert neuroprotective effects by influencing brain function, regulating blood flow, and modulating neuroinflammation via interactions with specific receptors and epigenetic modulation.
	2. Role in neurodegenerative diseases: Reduced SCFAs levels are implicated in neurodegenerative diseases like Alzheimer’s and Parkinson’s. They contribute to intestinal barrier impairment, the release of pro-inflammatory molecules, and microglial activation, ultimately impacting disease progression.
	3. Gut barrier function and motility: SCFAs promote mucus secretion and strengthen intestinal tight junctions, improving barrier integrity. SCFAs can influence nerve activity, neurotransmitters, and muscle contractions.

**Table 7:** Mechanisms of SCFAs in metabolic diseases. Abbreviations: SCFAs = Short-Chain Fatty Acids, GLP-1 = Glucagon-like Peptide-1, PYY = Peptide YY, BAT = Brown Adipose Tissue, GPR41 and GPR43 = G-Protein-Coupled Receptors 41 and 43, IGN = Intestinal Gluconeogenesis, TNF-α = Tumor Necrosis Factor-alpha, IL-6 = Interleukin-6, NF-κB = Nuclear Factor-kappa B, FGF = Fibroblast Growth Factor, FXR = Farnesoid X Receptor.

Disease	Proposed Mechanisms of SCFAs Protection/Risk
Obesity	1. Appetite Regulation: SCFAs can stimulate the release of Peptide YY (PYY) and glucagon-like peptide-1 (GLP-1) from gut endocrine cells. These hormones act centrally in the hypothalamus to signal satiety and decrease appetite.
	2. Fat Storage and Metabolism: Increased SCFAs-mediated adipocyte activity might favor fat storage in subcutaneous adipose tissue. SCFAs might enhance brown adipose tissue (BAT) activity, promoting thermogenesis and potentially increasing energy expenditure.
	3. Metabolic effects: SCFAs activate GPR41 and GPR43 receptors on fat and immune cells, potentially influencing insulin sensitivity, fat metabolism, inflammation, and, thus, weight regulation.
Type 2 Diabetes	1. Effects on glucose metabolism: SCFAs act as secretagogues for hormones such as GLP-1 and PYY, which enhance satiety and decrease appetite. GLP-1 enhances insulin secretion from the pancreas and reduces glucagon secretion, lowering blood sugar levels. In the liver, SCFAs inhibit glycolysis and gluconeogenesis, promoting glycogen synthesis and fatty acid oxidation. In skeletal muscle and adipose tissue, they improve glucose uptake and glycogen synthesis.
	2. Role in intestinal gluconeogenesis (IGN): SCFAs promote IGN production, which is crucial for glucose and energy homeostasis.
	3. Gut Health: SCFAs promote a healthy gut environment, which may be linked to a lower risk of developing diabetes.
Metabolic Dysfunction–Associated Steatotic Liver Disease	1. Improved Insulin Sensitivity: SCFAs can activate GPR43 on adipocytes and hepatocytes. GPR43 activation can stimulate insulin signaling pathways, leading to increased glucose uptake by these cells and potentially improving overall insulin sensitivity. SCFAs might also suppress gluconeogenesis in the liver.
	2. Anti-inflammatory Effects: SCFAs can modulate the activity of immune cells like macrophages in the liver. They might suppress pro-inflammatory cytokine production (e.g., TNF-α, IL-6) and promote the activity of regulatory T cells, creating an anti-inflammatory environment. SCFAs inhibit the NF-κB signaling pathway, a key player in inflammatory responses.
	3. Gut-Liver Axis: SCFAs might also influence Fibroblast Growth Factor (FGF) signaling pathways in the gut-liver axis, potentially impacting bile acid metabolism and hepatocyte function. SCFAs might stimulate the enterohepatic circulation of bile acids. SCFAs-mediated bile acid signaling can activate FXR, a nuclear receptor in the liver, potentially influencing hepatic lipid metabolism and reducing steatosis.

## Abbreviations

AMPK	5’ Adenosine Monophosphate-Activated Protein Kinase
AOM	Azoxymethane
APP	Amyloid Precursor Protein, Precursor Protein 695
Aβ	Amyloid Β
BAD	Bile Acid Diarrhea
BAT	Brown Adipose Tissue
BCoAT	Butyryl-Coa:Acetate Coatransferase
BCRP	Breast Cancer Resistance Protein
C2	Acetate
C3	Propionate
C4	Butyrate
CaBu	Calcium Butyrate
CD	Crohn’s Disease
CRC	Colorectal Cancer
DGBI	Disorder Of Gut-Brain Interaction
DSS	Dextran Sodium Sulfate
*F. prausnitzii*	*Faecalibacterium prausnitzii*
FAD	Familial Alzheimer’s Disease
FAP	Familial Adenomatous Polyposis
FGF	Fibroblast Growth Factor
FMT	Fecal Microbiota Transplantation
FOS	Fructooligosaccharides
FXR	Farnesoid X Receptor
GLP-1/2	Glucagon-Like Peptide-1/2
GLUT4	Glucose Transporter Type 4
GOS	Galactooligosaccharides
GPCRs = GPR41-GPR43-109a	G-Protein-Coupled-Receptors 41-43-109a
Gpr43(−/−)	GPR43-Deficient
HbA1c	Glycated Hemoglobin
HDACs	Histone Deacetylases
HFD	High-Fat Diet
HOMA-IR	Homeostatic Model Assessment Of Insulin Resistance
IBD	Inflammatory Bowel Diseases
IBS, IBS-C, IBS-D, IBS-M	Irritable Bowel Syndrome, Constipation, Diarrhea, Mixed Stool
IGN	Intestinal Gluconeogenesis
IL-6,10,17,18	(Cytokine) Interleukine-6-10-17-18
LPS	Lipopolysaccharides
MAFLD	Metabolic-Associated Fatty Liver Disease
MASLD	Metabolic-Dysfunction Associated Steatotic Liver Disease
MCT1-4	Monocarboxylate Transporters 1-4
MPTP	1-Methyl-4-Phenyl-1,2,3,6-Tetrahydropyridine
MS	Multiple Sclerosis
MSI-h	Microsatellite Instability-High
MYD88/NF-κB	TLR4/Myeloid Differentiation Primary Response 88
NaBu	Sodium Butyrate
NAFLD	Non-Alcoholic Fatty Liver Disease
NF-κB	Nuclear Factor Kappa-Light-Chain-Enhancer of Activated B Cells
Ngn3	Neurogenin-3
NLRP3	Nod-Like Receptor Family Pyrin Domain Containing 3
NOD	Nonobese Diabetic
PD	Parkinson’s Disease
PGC-1α	Peroxisome Coactivator-1 Alpha
PKM2	Pyruvate Kinase Muscle Isozyme 2
PPARγ	Peroxisome Proliferator-Activated Receptor-γ
PSC	Primary Sclerosing Cholangitis
PSEN1	Presenilin-1
PYY	Peptide YY
rCDI	Recurrent *Clostridioides Difficile* Infection
RCT	Randomized Controlled Trial
SCFAs	Short-Chain Fatty Acids
SLAB51	A Mixture Of Lactic Acid Bacteria And Bifidobacteria
SMCT1	Sodium-Coupled Monocarboxylate Transporter 1
SRY	Sex Determining Region Y
SRY Sox2	(Sex Determining Region Y)-Box 2
T1/2D	Type 1/2 Diabetes
T2DM	Type 2 Diabetes Mellitus
Th1- Th17	T-Helper 1 Cells- T-Helper 17 Cells
TLRs- TLR4- TLR2	Toll-Like Receptors-4-2
TNF-α	Tumor Necrosis Factor-Alpha
Treg	Regulatory T Cells
UC	Ulcerative Colitis
ZO-1	Zonula Occludens-1
5XFAD	Transgenic Mice Overexpress Mutant Human Amyloid Beta

## References

[1] Musso G., Gambino R., Cassader M. (2010). Gut Microbiota as a Regulator of Energy Homeostasis and Ectopic Fat Deposition: Mechanisms and Implications for Metabolic Disorders. Curr. Opin. Lipidol..

[2] Høverstad T. (1986). Studies of Short-Chain Fatty Acid Absorption in Man. Scand. J. Gastroenterol..

[3] Macfarlane S., Macfarlane G.T. (2003). Regulation of Short-Chain Fatty Acid Production. Proc. Nutr. Soc..

[4] Siddiqui M.T., Cresci G.A.M. (2021). The Immunomodulatory Functions of Butyrate. J. Inflamm. Res..

[5] Guilloteau P., Martin L., Eeckhaut V., Ducatelle R., Zabielski R., Van Immerseel F. (2010). From the Gut to the Peripheral Tissues: The Multiple Effects of Butyrate. Nutr. Res. Rev..

[6] Hosseini E., Grootaert C., Verstraete W., Van De Wiele T. (2011). Propionate as a Health-Promoting Microbial Metabolite in the Human Gut. Nutr. Rev..

[7] Hernández M.A.G., Canfora E.E., Jocken J.W.E., Blaak E.E. (2019). The Short-Chain Fatty Acid Acetate in Body Weight Control and Insulin Sensitivity. Nutrients.

[8] Culp E.J., Goodman A.L. (2023). Cross-Feeding in the Gut Microbiome: Ecology and Mechanisms. Cell Host Microbe.

[9] Germerodt S., Bohl K., Lück A., Pande S., Schröter A., Kaleta C., Schuster S., Kost C. (2016). Pervasive Selection for Cooperative Cross-Feeding in Bacterial Communities. PLoS Comput. Biol..

[10] Rasouli-Saravani A., Jahankhani K., Moradi S., Gorgani M., Shafaghat Z., Mirsanei Z., Mehmandar A., Mirzaei R. (2023). Role of Microbiota Short-Chain Fatty Acids in the Pathogenesis of Autoimmune Diseases. Biomed. Pharmacother..

[11] Hosmer J., McEwan A.G., Kappler U. (2023). Bacterial Acetate Metabolism and Its Influence on Human Epithelia. Emerg. Top. Life Sci..

[12] Wong J.M.W., De Souza R., Kendall C.W.C., Emam A., Jenkins D.J.A. (2006). Colonic Health: Fermentation and Short Chain Fatty Acids. J. Clin. Gastroenterol..

[13] Schug Z.T., Voorde J.V., Gottlieb E. (2016). The Metabolic Fate of Acetate in Cancer. Nat. Rev. Cancer.

[14] Li G., Xie C., Lu S., Nichols R.G., Tian Y., Li L., Patel D., Ma Y., Brocker C.N., Yan T. (2017). Intermittent Fasting Promotes White Adipose Browning and Decreases Obesity by Shaping the Gut Microbiota. Cell Metab..

[15] Remely M., Hippe B., Geretschlaeger I., Stegmayer S., Hoefinger I., Haslberger A. (2015). Increased Gut Microbiota Diversity and Abundance of Faecalibacterium Prausnitzii and Akkermansia after Fasting: A Pilot Study. Wien. Klin. Wochenschr..

[16] Dao M.C., Everard A., Aron-Wisnewsky J., Sokolovska N., Prifti E., Verger E.O., Kayser B.D., Levenez F., Chilloux J., Hoyles L. (2016). *Akkermansia muciniphila* and Improved Metabolic Health during a Dietary Intervention in Obesity: Relationship with Gut Microbiome Richness and Ecology. Gut.

[17] Duncan S.H. (2002). Growth Requirements and Fermentation Products of Fusobacterium Prausnitzii, and a Proposal to Reclassify It as Faecalibacterium Prausnitzii Gen. Nov., Comb. Nov. Int. J. Syst. Evol. Microbiol..

[18] Reichardt N., Duncan S.H., Young P., Belenguer A., McWilliam Leitch C., Scott K.P., Flint H.J., Louis P. (2014). Phylogenetic Distribution of Three Pathways for Propionate Production within the Human Gut Microbiota. ISME J..

[19] Louis P., Flint H.J. (2017). Formation of Propionate and Butyrate by the Human Colonic Microbiota. Environ. Microbiol..

[20] Smith E.A., Macfarlane G.T. (1998). Enumeration of Amino Acid Fermenting Bacteria in the Human Large Intestine: Effects of pH and Starch on Peptide Metabolism and Dissimilation of Amino Acids. FEMS Microbiol. Ecol..

[21] Gänzle M.G. (2015). Lactic Metabolism Revisited: Metabolism of Lactic Acid Bacteria in Food Fermentations and Food Spoilage. Curr. Opin. Food Sci..

[22] Jan G., Belzacq A.-S., Haouzi D., Rouault A., Kroemer G., Brenner C. (2002). Propionibacteria Induce Apoptosis of Colorectal Carcinoma Cells via Short-Chain Fatty Acids Acting on Mitochondria. Cell Death Differ..

[23] Louis P., Duncan S.H., McCrae S.I., Millar J., Jackson M.S., Flint H.J. (2004). Restricted Distribution of the Butyrate Kinase Pathway among Butyrate-Producing Bacteria from the Human Colon. J. Bacteriol..

[24] Quévrain E., Maubert M.A., Michon C., Chain F., Marquant R., Tailhades J., Miquel S., Carlier L., Bermúdez-Humarán L.G., Pigneur B. (2016). Identification of an Anti-Inflammatory Protein from *Faecalibacterium prausnitzii*, a Commensal Bacterium Deficient in Crohn’s Disease. Gut.

[25] Louis P., Flint H.J. (2009). Diversity, Metabolism and Microbial Ecology of Butyrate-Producing Bacteria from the Human Large Intestine. FEMS Microbiol. Lett..

[26] Oliphant K., Parreira V.R., Cochrane K., Allen-Vercoe E. (2019). Drivers of Human Gut Microbial Community Assembly: Coadaptation, Determinism and Stochasticity. ISME J..

[27] Bui T.P.N., Ritari J., Boeren S., De Waard P., Plugge C.M., De Vos W.M. (2015). Production of Butyrate from Lysine and the Amadori Product Fructoselysine by a Human Gut Commensal. Nat. Commun..

[28] Buckel W. (2001). Unusual Enzymes Involved in Five Pathways of Glutamate Fermentation. Appl. Microbiol. Biotechnol..

[29] Potrykus J., White R.L., Bearne S.L. (2008). Proteomic Investigation of Amino Acid Catabolism in the Indigenous Gut Anaerobe *Fusobacterium varium*. Proteomics.

[30] Kanehisa M., Sato Y., Kawashima M., Furumichi M., Tanabe M. (2016). KEGG as a Reference Resource for Gene and Protein Annotation. Nucleic Acids Res..

[31] Holscher H.D. (2017). Dietary Fiber and Prebiotics and the Gastrointestinal Microbiota. Gut Microbes.

[32] Gibson G.R., Probert H.M., Loo J.V., Rastall R.A., Roberfroid M.B. (2004). Dietary Modulation of the Human Colonic Microbiota: Updating the Concept of Prebiotics. Nutr. Res. Rev..

[33] De Vuyst L., Leroy F. (2011). Cross-Feeding between Bifidobacteria and Butyrate-Producing Colon Bacteria Explains Bifdobacterial Competitiveness, Butyrate Production, and Gas Production. Int. J. Food Microbiol..

[34] Belenguer A., Duncan S.H., Holtrop G., Anderson S.E., Lobley G.E., Flint H.J. (2007). Impact of pH on Lactate Formation and Utilization by Human Fecal Microbial Communities. Appl. Environ. Microbiol..

[35] Molis C., Flourié B., Ouarne F., Gailing M., Lartigue S., Guibert A., Bornet F., Galmiche J. (1996). Digestion, Excretion, and Energy Value of Fructooligosaccharides in Healthy Humans. Am. J. Clin. Nutr..

[36] Belenguer A., Duncan S.H., Calder A.G., Holtrop G., Louis P., Lobley G.E., Flint H.J. (2006). Two Routes of Metabolic Cross-Feeding between Bifidobacterium Adolescentis and Butyrate-Producing Anaerobes from the Human Gut. Appl. Environ. Microbiol..

[37] Falony G., Vlachou A., Verbrugghe K., Vuyst L.D. (2006). Cross-Feeding between Bifidobacterium Longum BB536 and Acetate-Converting, Butyrate-Producing Colon Bacteria during Growth on Oligofructose. Appl. Environ. Microbiol..

[38] Louis P., Scott K.P., Duncan S.H., Flint H.J. (2007). Understanding the Effects of Diet on Bacterial Metabolism in the Large Intestine. J. Appl. Microbiol..

[39] Kanauchi O., Fujiyama Y., Mitsuyama K., Araki Y., Ishii T., Nakamura T., Hitomi Y., Agata K., Saiki T., Andoh A. (1999). Increased Growth of Bifidobacterium and Eubacterium by Germinated Barley Foodstuff, Accompanied by Enhanced Butyrate Production in Healthy Volunteers. Int. J. Mol. Med..

[40] Chassard C., Bernalier-Donadille A. (2006). H_2_ and Acetate Transfers during Xylan Fermentation between a Butyrate-Producing Xylanolytic Species and Hydrogenotrophic Microorganisms from the Human Gut. FEMS Microbiol. Lett..

[41] Velázquez O.C., Lederer H.M., Rombeau J.L. (1997). Butyrate and the Colonocyte. Production, Absorption, Metabolism, and Therapeutic Implications. Adv. Exp. Med. Biol..

[42] Garcia C.K., Goldstein J.L., Pathak R.K., Anderson R.G., Brown M.S. (1994). Molecular Characterization of a Membrane Transporter for Lactate, Pyruvate, and Other Monocarboxylates: Implications for the Cori Cycle. Cell.

[43] Sivaprakasam S., Bhutia Y.D., Yang S., Ganapathy V. (2018). Short-Chain Fatty Acid Transporters: Role in Colonic Homeostasis. Compr. Physiol..

[44] Coady M.J., Chang M.-H., Charron F.M., Plata C., Wallendorff B., Sah J.F., Markowitz S.D., Romero M.F., Lapointe J.-Y. (2004). The Human Tumour Suppressor Gene SLC5A8 Expresses a Na^+^-Monocarboxylate Cotransporter. J. Physiol..

[45] Thibault R., Blachier F., Darcy-Vrillon B., de Coppet P., Bourreille A., Segain J.-P. (2010). Butyrate Utilization by the Colonic Mucosa in Inflammatory Bowel Diseases: A Transport Deficiency. Inflamm. Bowel Dis..

[46] Lambert D.W., Wood I.S., Ellis A., Shirazi-Beechey S.P. (2002). Molecular Changes in the Expression of Human Colonic Nutrient Transporters during the Transition from Normality to Malignancy. Br. J. Cancer.

[47] Chiche J., Ricci J.-E., Pouysségur J. (2013). Tumor Hypoxia and Metabolism—Towards Novel Anticancer Approaches. Ann. Endocrinol..

[48] Jones N.P., Schulze A. (2012). Targeting Cancer Metabolism—Aiming at a Tumour’s Sweet-Spot. Drug Discov. Today.

[49] Marchiq I., Pouysségur J. (2016). Hypoxia, Cancer Metabolism and the Therapeutic Benefit of Targeting Lactate/H(+) Symporters. J. Mol. Med..

[50] Bolden J.E., Peart M.J., Johnstone R.W. (2006). Anticancer Activities of Histone Deacetylase Inhibitors. Nat. Rev. Drug Discov..

[51] Fishbein W.N. (1986). Lactate Transporter Defect: A New Disease of Muscle. Science.

[52] Gupta N., Martin P.M., Prasad P.D., Ganapathy V. (2006). SLC5A8 (SMCT1)-Mediated Transport of Butyrate Forms the Basis for the Tumor Suppressive Function of the Transporter. Life Sci..

[53] Dietrich C.G., Vehr A.-K., Martin I.V., Gassler N., Rath T., Roeb E., Schmitt J., Trautwein C., Geier A. (2011). Downregulation of Breast Cancer Resistance Protein in Colon Adenomas Reduces Cellular Xenobiotic Resistance and Leads to Accumulation of a Food-Derived Carcinogen. Int. J. Cancer.

[54] Gonçalves P., Gregório I., Martel F. (2011). The Short-Chain Fatty Acid Butyrate Is a Substrate of Breast Cancer Resistance Protein. Am. J. Physiol. Cell Physiol..

[55] Yang G., Chen S., Deng B., Tan C., Deng J., Zhu G., Yin Y., Ren W. (2018). Implication of G Protein-Coupled Receptor 43 in Intestinal Inflammation: A Mini-Review. Front. Immunol..

[56] Jomova K., Makova M., Alomar S.Y., Alwasel S.H., Nepovimova E., Kuca K., Rhodes C.J., Valko M. (2022). Essential Metals in Health and Disease. Chem.-Biol. Interact..

[57] Wilck N., Balogh A., Markó L., Bartolomaeus H., Müller D.N. (2019). The Role of Sodium in Modulating Immune Cell Function. Nat. Rev. Nephrol..

[58] Gaschott T., Stein J., Reichrath J., Tilgen W., Friedrich M. (2003). Short-Chain Fatty Acids and Colon Cancer Cells: The Vitamin D Receptor—Butyrate Connection. Vitamin D Analogs in Cancer Prevention and Therapy.

[59] Jantsch J., Schatz V., Friedrich D., Schröder A., Kopp C., Siegert I., Maronna A., Wendelborn D., Linz P., Binger K.J. (2015). Cutaneous Na^+^ Storage Strengthens the Antimicrobial Barrier Function of the Skin and Boosts Macrophage-Driven Host Defense. Cell Metab..

[60] Byles V., Covarrubias A.J., Ben-Sahra I., Lamming D.W., Sabatini D.M., Manning B.D., Horng T. (2013). The TSC-mTOR Pathway Regulates Macrophage Polarization. Nat. Commun..

[61] Hodgkinson K., El Abbar F., Dobranowski P., Manoogian J., Butcher J., Figeys D., Mack D., Stintzi A. (2023). Butyrate’s Role in Human Health and the Current Progress towards Its Clinical Application to Treat Gastrointestinal Disease. Clin. Nutr..

[62] Rhodes J.M. (2021). Nutrition and Gut Health: The Impact of Specific Dietary Components—It’s Not Just Five-a-Day. Proc. Nutr. Soc..

[63] Kannampalli P., Shaker R., Sengupta J.N. (2011). Colonic Butyrate- Algesic or Analgesic?: Colonic Butyrate Function. Neurogastroenterol. Motil..

[64] Facchin S., Vitulo N., Calgaro M., Buda A., Romualdi C., Pohl D., Perini B., Lorenzon G., Marinelli C., D’Incà R. (2020). Microbiota Changes Induced by Microencapsulated Sodium Butyrate in Patients with Inflammatory Bowel Disease. Neurogastroenterol. Motil..

[65] Anshory M., Effendi R.M.R.A., Kalim H., Dwiyana R.F., Suwarsa O., Nijsten T.E.C., Nouwen J.L., Thio H.B. (2023). Butyrate Properties in Immune-Related Diseases: Friend or Foe?. Fermentation.

[66] Guillemot F., Colombel J.F., Neut C., Verplanck N., Lecomte M., Romond C., Paris J.C., Cortot A. (1991). Treatment of Diversion Colitis by Short-Chain Fatty Acids: Prospective and Double-Blind Study. Dis. Colon Rectum.

[67] Scheppach W., Sommer H., Kirchner T., Paganelli G.M., Bartram P., Christl S., Richter F., Dusel G., Kasper H. (1992). Effect of Butyrate Enemas on the Colonic Mucosa in Distal Ulcerative Colitis. Gastroenterology.

[68] Steinhart A.H., Brzezinski A., Baker J.P. (1994). Treatment of Refractory Ulcerative Proctosigmoiditis with Butyrate Enemas. Am. J. Gastroenterol..

[69] Vernia P., Marcheggiano A., Caprilli R., Frieri G., Corrao G., Valpiani D., DI Paolo M.C., Paoluzi P., Torsoli A. (1995). Short-chain Fatty Acid Topical Treatment in Distal Ulcerative Colitis. Aliment. Pharmacol. Ther..

[70] Steinhart A.H., Hiruki T., Brzezinski A., Baker J.P. (1996). Treatment of Left-Sided Ulcerative Colitis with Butyrate Enemas: A Controlled Trial. Aliment. Pharmacol. Ther..

[71] Scheppach W., Christl S.U., Bartram H.P., Richter F., Kasper H. (1997). Effects of Short-Chain Fatty Acids on the Inflamed Colonic Mucosa. Scand. J. Gastroenterol. Suppl..

[72] Pinto A., Fidalgo P., Cravo M., Midões J., Chaves P., Rosa J., Birto M.D.A., Leitão C.N. (1999). Short Chain Fatty Acids Are Effective in Short-Term Treatment of Chronic Radiation Proctitis: Randomized, Double-Blind, Controlled Trial. Dis. Colon Rectum.

[73] Vernia P., Monteleone G., Grandinetti G., Villotti G., Di Giulio E., Frieri G., Marcheggiano A., Pallone F., Caprilli R., Torsoli A. (2000). Combined Oral Sodium Butyrate and Mesalazine Treatment Compared to Oral Mesalazine Alone in Ulcerative Colitis: Randomized, Double-Blind, Placebo-Controlled Pilot Study. Dig. Dis. Sci..

[74] Vernia P., Fracasso P., Casale V., Villotti G., Marcheggiano A., Stigliano V., Pinnaro P., Bagnardi V., Caprilli R. (2000). Topical Butyrate for Acute Radiation Proctitis: Randomised, Crossover Trial. Lancet.

[75] Lührs H., Gerke T., Müller J.G., Melcher R., Schauber J., Boxberge F., Scheppach W., Menzel T. (2002). Butyrate Inhibits NF-kappaB Activation in Lamina Propria Macrophages of Patients with Ulcerative Colitis. Scand. J. Gastroenterol..

[76] Vernia P., Annese V., Bresci G., D’albasio G., D’incà R., Giaccari S., Ingrosso M., Mansi C., Riegler G., Valpiani D. (2003). Topical Butyrate Improves Efficacy of 5-ASA in Refractory Distal Ulcerative Colitis: Results of a Multicentre Trial. Eur. J. Clin. Investig..

[77] Di Sabatino A., Morera R., Ciccocioppo R., Cazzola P., Gotti S., Tinozzi F.P., Tinozzi S., Corazza G.R. (2005). Oral Butyrate for Mildly to Moderately Active Crohn’s Disease. Aliment. Pharmacol. Ther..

[78] Assisi R.F., GISDI Study Group Combined Butyric Acid/Mesalazine Treatment in Ulcerative Colitis with Mild-Moderate Activity (2008). Results of a Multicentre Pilot Study. Minerva Gastroenterol. E Dietol..

[79] Vanhoutvin S.A.L.W., Troost F.J., Kilkens T.O.C., Lindsey P.J., Hamer H.M., Jonkers D.M.A.E., Venema K., Brummer R.J.M. (2009). The Effects of Butyrate Enemas on Visceral Perception in Healthy Volunteers. Neurogastroenterol. Motil..

[80] Hamer H.M., Jonkers D.M.A.E., Vanhoutvin S.A.L.W., Troost F.J., Rijkers G., de Bruïne A., Bast A., Venema K., Brummer R.J.M. (2010). Effect of Butyrate Enemas on Inflammation and Antioxidant Status in the Colonic Mucosa of Patients with Ulcerative Colitis in Remission. Clin. Nutr..

[81] Banasiewicz T., Krokowicz L., Stojcev Z., Kaczmarek B.F., Kaczmarek E., Maik J., Marciniak R., Krokowicz P., Walkowiak J., Drews M. (2013). Microencapsulated Sodium Butyrate Reduces the Frequency of Abdominal Pain in Patients with Irritable Bowel Syndrome. Color. Dis. Off. J. Assoc. Coloproctology Great Br. Irel..

[82] Krokowicz L., Stojcev Z., Kaczmarek B.F., Kociemba W., Kaczmarek E., Walkowiak J., Krokowicz P., Drews M., Banasiewicz T. (2014). Microencapsulated Sodium Butyrate Administered to Patients with Diverticulosis Decreases Incidence of Diverticulitis—A Prospective Randomized Study. Int. J. Color. Dis..

[83] Krokowicz L., Kaczmarek B.F., Krokowicz P., Stojcev Z., Mackiewicz J., Walkowiak J., Drews M., Banasiewicz T. (2014). Sodium Butyrate and Short Chain Fatty Acids in Prevention of Travellers’ Diarrhoea: A Randomized Prospective Study. Travel. Med. Infect. Dis..

[84] Maggio A., Magli A., Rancati T., Fiorino C., Valvo F., Fellin G., Ricardi U., Munoz F., Cosentino D., Cazzaniga L.F. (2014). Daily Sodium Butyrate Enema for the Prevention of Radiation Proctitis in Prostate Cancer Patients Undergoing Radical Radiation Therapy: Results of a Multicenter Randomized Placebo-Controlled Dose-Finding Phase 2 Study. Int. J. Radiat. Oncol. Biol. Phys..

[85] Luceri C., Femia A.P., Fazi M., Di Martino C., Zolfanelli F., Dolara P., Tonelli F. (2016). Effect of Butyrate Enemas on Gene Expression Profiles and Endoscopic/Histopathological Scores of Diverted Colorectal Mucosa: A Randomized Trial. Dig. Liver Dis..

[86] Roshanravan N., Mahdavi R., Alizadeh E., Jafarabadi M., Hedayati M., Ghavami A., Alipour S., Alamdari N., Barati M., Ostadrahimi A. (2017). Effect of Butyrate and Inulin Supplementation on Glycemic Status, Lipid Profile and Glucagon-Like Peptide 1 Level in Patients with Type 2 Diabetes: A Randomized Double-Blind, Placebo-Controlled Trial. Horm. Metab. Res..

[87] Vernero M., De Blasio F., Ribaldone D.G., Bugianesi E., Pellicano R., Saracco G.M., Astegiano M., Caviglia G.P. (2020). The Usefulness of Microencapsulated Sodium Butyrate Add-On Therapy in Maintaining Remission in Patients with Ulcerative Colitis: A Prospective Observational Study. J. Clin. Med..

[88] De Groot P.F., Nikolic T., Imangaliyev S., Bekkering S., Duinkerken G., Keij F.M., Herrema H., Winkelmeijer M., Kroon J., Levin E. (2020). Oral Butyrate Does Not Affect Innate Immunity and Islet Autoimmunity in Individuals with Longstanding Type 1 Diabetes: A Randomised Controlled Trial. Diabetologia.

[89] Coppola S., Nocerino R., Paparo L., Bedogni G., Calignano A., Di Scala C., De Giovanni Di Santa Severina A.F., De Filippis F., Ercolini D., Berni Canani R. (2022). Therapeutic Effects of Butyrate on Pediatric Obesity: A Randomized Clinical Trial. JAMA Netw. Open.

[90] Pietrzak A., Banasiuk M., Szczepanik M., Borys-Iwanicka A., Pytrus T., Walkowiak J., Banaszkiewicz A. (2022). Sodium Butyrate Effectiveness in Children and Adolescents with Newly Diagnosed Inflammatory Bowel Diseases—Randomized Placebo-Controlled Multicenter Trial. Nutrients.

[91] Khosravi Z., Hadi A., Tutunchi H., Asghari-Jafarabadi M., Naeinie F., Roshanravan N., Ostadrahimi A., Fadel A. (2022). The Effects of Butyrate Supplementation on Glycemic Control, Lipid Profile, Blood Pressure, Nitric Oxide Level and Glutathione Peroxidase Activity in Type 2 Diabetic Patients: A Randomized Triple -Blind, Placebo-Controlled Trial. Clin. Nutr. ESPEN.

[92] Qaisar R., Karim A., Muhammad T., Ahmad F. (2024). Butyrate Supplementation Reduces Sarcopenia by Repairing Neuromuscular Junction in Patients with Chronic Obstructive Pulmonary Disease. Respir. Med..

[93] Schwarz A., Bruhs A., Schwarz T. (2017). The Short-Chain Fatty Acid Sodium Butyrate Functions as a Regulator of the Skin Immune System. J. Investig. Dermatol..

[94] Stacey S.K., McEleney M. (2021). Topical Corticosteroids: Choice and Application. Am. Fam. Physician.

[95] Delzenne N.M., Williams C.M. (2002). Prebiotics and Lipid Metabolism. Curr. Opin. Lipidol..

[96] Oba M., Allen M.S. (2003). Intraruminal Infusion of Propionate Alters Feeding Behavior and Decreases Energy Intake of Lactating Dairy Cows. J. Nutr..

[97] Li C.J., Elsasser T.H. (2005). Butyrate-Induced Apoptosis and Cell Cycle Arrest in Bovine Kidney Epithelial Cells: Involvement of Caspase and Proteasome Pathways1. J. Anim. Sci..

[98] Kamp F., Hamilton J.A. (2006). How Fatty Acids of Different Chain Length Enter and Leave Cells by Free Diffusion. Prostaglandins Leukot. Essent. Fat. Acids.

[99] Cummings J.H., Pomare E.W., Branch H.W.J., Naylor C.P.E., MacFarlane G.T. (1987). Short Chain Fatty Acids in Human Large Intestine, Portal, Hepatic and Venous Blood. Gut.

[100] Park J., Kim M., Kang S.G., Jannasch A.H., Cooper B., Patterson J., Kim C.H. (2015). Short-Chain Fatty Acids Induce Both Effector and Regulatory T Cells by Suppression of Histone Deacetylases and Regulation of the mTOR–S6K Pathway. Mucosal Immunol..

[101] Duscha A., Gisevius B., Hirschberg S., Yissachar N., Stangl G.I., Eilers E., Bader V., Haase S., Kaisler J., David C. (2020). Propionic Acid Shapes the Multiple Sclerosis Disease Course by an Immunomodulatory Mechanism. Cell.

[102] Chambers E.S., Viardot A., Psichas A., Morrison D.J., Murphy K.G., Zac-Varghese S.E.K., MacDougall K., Preston T., Tedford C., Finlayson G.S. (2015). Effects of Targeted Delivery of Propionate to the Human Colon on Appetite Regulation, Body Weight Maintenance and Adiposity in Overweight Adults. Gut.

[103] Chambers E.S., Byrne C.S., Morrison D.J., Murphy K.G., Preston T., Tedford C., Garcia-Perez I., Fountana S., Serrano-Contreras J.I., Holmes E. (2019). Dietary Supplementation with Inulin-Propionate Ester or Inulin Improves Insulin Sensitivity in Adults with Overweight and Obesity with Distinct Effects on the Gut Microbiota, Plasma Metabolome and Systemic Inflammatory Responses: A Randomised Cross-over Trial. Gut.

[104] Malkova D., Polyviou T., Rizou E., Gerasimidis K., Chambers E.S., Preston T., Tedford M.C., Frost G., Morrison D.J. (2020). Moderate Intensity Exercise Training Combined with Inulin-Propionate Ester Supplementation Increases Whole Body Resting Fat Oxidation in Overweight Women. Metabolism.

[105] Haghikia A., Zimmermann F., Schumann P., Jasina A., Roessler J., Schmidt D., Heinze P., Kaisler J., Nageswaran V., Aigner A. (2021). Propionate Attenuates Atherosclerosis by Immune-Dependent Regulation of Intestinal Cholesterol Metabolism. Eur. Heart J..

[106] Deleu S., Machiels K., Raes J., Verbeke K., Vermeire S. (2021). Short Chain Fatty Acids and Its Producing Organisms: An Overlooked Therapy for IBD?. EBioMedicine.

[107] Gill P.A., van Zelm M.C., Muir J.G., Gibson P.R. (2018). Review Article: Short Chain Fatty Acids as Potential Therapeutic Agents in Human Gastrointestinal and Inflammatory Disorders. Aliment. Pharmacol. Ther..

[108] Offei B., Vandecruys P., De Graeve S., Foulquié-Moreno M.R., Thevelein J.M. (2019). Unique Genetic Basis of the Distinct Antibiotic Potency of High Acetic Acid Production in the Probiotic Yeast *Saccharomyces cerevisiae* Var. boulardii. Genome Res..

[109] Kondo T., Kishi M., Fushimi T., Kaga T. (2009). Acetic Acid Upregulates the Expression of Genes for Fatty Acid Oxidation Enzymes in Liver to Suppress Body Fat Accumulation. J. Agric. Food Chem..

[110] World Health Organization (2000). Evaluation of Certain Food Additives: Fifty-Third Report of the Joint FAO/WHO Expert Committee on Food Additives.

[111] Freeland K.R., Wolever T.M.S. (2010). Acute Effects of Intravenous and Rectal Acetate on Glucagon-like Peptide-1, Peptide YY, Ghrelin, Adiponectin and Tumour Necrosis Factor-α. Br. J. Nutr..

[112] Fernandes J., Vogt J., Wolever T.M. (2012). Intravenous Acetate Elicits a Greater Free Fatty Acid Rebound in Normal than Hyperinsulinaemic Humans. Eur. J. Clin. Nutr..

[113] Van Der Beek C.M., Canfora E.E., Lenaerts K., Troost F.J., Olde Damink S.W.M., Holst J.J., Masclee A.A.M., Dejong C.H.C., Blaak E.E. (2016). Distal, Not Proximal, Colonic Acetate Infusions Promote Fat Oxidation and Improve Metabolic Markers in Overweight/Obese Men. Clin. Sci..

[114] Canfora E.E., van der Beek C.M., Jocken J.W.E., Goossens G.H., Holst J.J., Olde Damink S.W.M., Lenaerts K., Dejong C.H.C., Blaak E.E. (2017). Colonic Infusions of Short-Chain Fatty Acid Mixtures Promote Energy Metabolism in Overweight/Obese Men: A Randomized Crossover Trial. Sci. Rep..

[115] Martin-Gallausiaux C., Marinelli L., Blottière H.M., Larraufie P., Lapaque N. (2021). SCFA: Mechanisms and Functional Importance in the Gut. Proc. Nutr. Soc..

[116] Sun M., Wu W., Liu Z., Cong Y. (2017). Microbiota Metabolite Short Chain Fatty Acids, GPCR, and Inflammatory Bowel Diseases. J. Gastroenterol..

[117] Blad C.C., Tang C., Offermanns S. (2012). G Protein-Coupled Receptors for Energy Metabolites as New Therapeutic Targets. Nat. Rev. Drug Discov..

[118] Candido E.P., Reeves R., Davie J.R. (1978). Sodium Butyrate Inhibits Histone Deacetylation in Cultured Cells. Cell.

[119] Sealy L., Chalkley R. (1978). The Effect of Sodium Butyrate on Histone Modification. Cell.

[120] Donohoe D.R., Collins L.B., Wali A., Bigler R., Sun W., Bultman S.J. (2012). The Warburg Effect Dictates the Mechanism of Butyrate-Mediated Histone Acetylation and Cell Proliferation. Mol. Cell.

[121] Visekruna A., Luu M. (2021). The Role of Short-Chain Fatty Acids and Bile Acids in Intestinal and Liver Function, Inflammation, and Carcinogenesis. Front. Cell Dev. Biol..

[122] Bourassa M.W., Alim I., Bultman S.J., Ratan R.R. (2016). Butyrate, Neuroepigenetics and the Gut Microbiome: Can a High Fiber Diet Improve Brain Health?. Neurosci. Lett..

[123] Akhtar M., Chen Y., Ma Z., Zhang X., Shi D., Khan J.A., Liu H. (2022). Gut Microbiota-Derived Short Chain Fatty Acids Are Potential Mediators in Gut Inflammation. Anim. Nutr..

[124] Barberio B., Facchin S., Patuzzi I., Ford A.C., Massimi D., Valle G., Sattin E., Simionati B., Bertazzo E., Zingone F. (2022). A Specific Microbiota Signature Is Associated to Various Degrees of Ulcerative Colitis as Assessed by a Machine Learning Approach. Gut Microbes.

[125] Segain J., de la Bletiere D.R., Bourreille A., Leray V., Gervois N., Rosales C., Ferrier L., Bonnet C., Blottiere H., Galmiche J. (2000). Butyrate Inhibits Inflammatory Responses through NFκB Inhibition: Implications for Crohn’s Disease. Gut.

[126] Machiels K., Joossens M., Sabino J., De Preter V., Arijs I., Eeckhaut V., Ballet V., Claes K., Van Immerseel F., Verbeke K. (2014). A Decrease of the Butyrate-Producing Species Roseburia Hominis and Faecalibacterium Prausnitzii Defines Dysbiosis in Patients with Ulcerative Colitis. Gut.

[127] Quaglio A.E.V., Grillo T.G., De Oliveira E.C.S., Di Stasi L.C., Sassaki L.Y. (2022). Gut Microbiota, Inflammatory Bowel Disease and Colorectal Cancer. World J. Gastroenterol..

[128] Gonçalves P., Araújo J.R., Di Santo J.P. (2018). A Cross-Talk between Microbiota-Derived Short-Chain Fatty Acids and the Host Mucosal Immune System Regulates Intestinal Homeostasis and Inflammatory Bowel Disease. Inflamm. Bowel Dis..

[129] Zhang Y., Si X., Yang L., Wang H., Sun Y., Liu N. (2022). Association between Intestinal Microbiota and Inflammatory Bowel Disease. Anim. Model. Exp. Med..

[130] Ma J., Piao X., Mahfuz S., Long S., Wang J. (2022). The Interaction among Gut Microbes, the Intestinal Barrier and Short Chain Fatty Acids. Anim. Nutr..

[131] Singh N., Gurav A., Sivaprakasam S., Brady E., Padia R., Shi H., Thangaraju M., Prasad P.D., Manicassamy S., Munn D.H. (2014). Activation of Gpr109a, Receptor for Niacin and the Commensal Metabolite Butyrate, Suppresses Colonic Inflammation and Carcinogenesis. Immunity.

[132] Samuel B.S., Shaito A., Motoike T., Rey F.E., Backhed F., Manchester J.K., Hammer R.E., Williams S.C., Crowley J., Yanagisawa M. (2008). Effects of the Gut Microbiota on Host Adiposity Are Modulated by the Short-Chain Fatty-Acid Binding G Protein-Coupled Receptor, Gpr41. Proc. Natl. Acad. Sci. USA.

[133] Smith P.M., Howitt M.R., Panikov N., Michaud M., Gallini C.A., Bohlooly-y M., Glickman J.N., Garrett W.S. (2013). The Microbial Metabolites, Short-Chain Fatty Acids, Regulate Colonic Treg Cell Homeostasis. Science.

[134] Maslowski K.M., Vieira A.T., Ng A., Kranich J., Sierro F., Yu D., Schilter H.C., Rolph M.S., Mackay F., Artis D. (2009). Regulation of Inflammatory Responses by Gut Microbiota and Chemoattractant Receptor GPR43. Nature.

[135] Arpaia N., Campbell C., Fan X., Dikiy S., van der Veeken J., deRoos P., Liu H., Cross J.R., Pfeffer K., Coffer P.J. (2013). Metabolites Produced by Commensal Bacteria Promote Peripheral Regulatory T-Cell Generation. Nature.

[136] Kovarik J.J., Tillinger W., Hofer J., Hölzl M.A., Heinzl H., Saemann M.D., Zlabinger G.J. (2011). Impaired Anti-Inflammatory Efficacy of n-Butyrate in Patients with IBD. Eur. J. Clin. Investig..

[137] Li A.-L., Ni W.-W., Zhang Q.-M., Li Y., Zhang X., Wu H.-Y., Du P., Hou J.-C., Zhang Y. (2020). Effect of Cinnamon Essential Oil on Gut Microbiota in the Mouse Model of Dextran Sodium Sulfate-Induced Colitis. Microbiol. Immunol..

[138] Sefat N.A.K., Mohammadi M.M., Hadjati J., Talebi S., Ajami M., Daneshvar H. (2019). Sodium Butyrate as a Histone Deacetylase Inhibitor Affects Toll-Like Receptor 4 Expression in Colorectal Cancer Cell Lines. Immunol. Investig..

[139] Parada Venegas D., De la Fuente M.K., Landskron G., González M.J., Quera R., Dijkstra G., Harmsen H.J.M., Faber K.N., Hermoso M.A. (2019). Short Chain Fatty Acids (SCFAs)-Mediated Gut Epithelial and Immune Regulation and Its Relevance for Inflammatory Bowel Diseases. Front. Immunol..

[140] Tews H.C., Elger T., Gunawan S., Fererberger T., Sommersberger S., Loibl J., Huss M., Liebisch G., Müller M., Kandulski A. (2023). Fecal Short Chain Fatty Acids and Urinary 3-Indoxyl Sulfate Do Not Discriminate between Patients with Crohn’s Disease and Ulcerative Colitis and Are Not of Diagnostic Utility for Predicting Disease Severity. Lipids Health Dis..

[141] Chen G., Ran X., Li B., Li Y., He D., Huang B., Fu S., Liu J., Wang W. (2018). Sodium Butyrate Inhibits Inflammation and Maintains Epithelium Barrier Integrity in a TNBS-Induced Inflammatory Bowel Disease Mice Model. EBioMedicine.

[142] Li G., Lin J., Zhang C., Gao H., Lu H., Gao X., Zhu R., Li Z., Li M., Liu Z. (2021). Microbiota Metabolite Butyrate Constrains Neutrophil Functions and Ameliorates Mucosal Inflammation in Inflammatory Bowel Disease. Gut Microbes.

[143] Zhou L., Zhang M., Wang Y., Dorfman R.G., Liu H., Yu T., Chen X., Tang D., Xu L., Yin Y. (2018). Faecalibacterium Prausnitzii Produces Butyrate to Maintain Th17/Treg Balance and to Ameliorate Colorectal Colitis by Inhibiting Histone Deacetylase 1. Inflamm. Bowel Dis..

[144] Dekker E., Tanis P.J., Vleugels J.L.A., Kasi P.M., Wallace M.B. (2019). Colorectal Cancer. Lancet.

[145] Hofseth L.J., Hebert J.R., Chanda A., Chen H., Love B.L., Pena M.M., Murphy E.A., Sajish M., Sheth A., Buckhaults P.J. (2020). Early-Onset Colorectal Cancer: Initial Clues and Current Views. Nat. Rev. Gastroenterol. Hepatol..

[146] Yang J., Wei H., Zhou Y., Szeto C.-H., Li C., Lin Y., Coker O.O., Lau H.C.H., Chan A.W.H., Sung J.J.Y. (2022). High-Fat Diet Promotes Colorectal Tumorigenesis through Modulating Gut Microbiota and Metabolites. Gastroenterology.

[147] Oh H., Kim H., Lee D.H., Lee A., Giovannucci E.L., Kang S.-S., Keum N. (2019). Different Dietary Fibre Sources and Risks of Colorectal Cancer and Adenoma: A Dose-Response Meta-Analysis of Prospective Studies. Br. J. Nutr..

[148] Gianfredi V., Salvatori T., Villarini M., Moretti M., Nucci D., Realdon S. (2018). Is Dietary Fibre Truly Protective against Colon Cancer? A Systematic Review and Meta-Analysis. Int. J. Food Sci. Nutr..

[149] Alvandi E., Wong W.K.M., Joglekar M.V., Spring K.J., Hardikar A.A. (2022). Short-Chain Fatty Acid Concentrations in the Incidence and Risk-Stratification of Colorectal Cancer: A Systematic Review and Meta-Analysis. BMC Med..

[150] Sepich-Poore G.D., Zitvogel L., Straussman R., Hasty J., Wargo J.A., Knight R. (2021). The Microbiome and Human Cancer. Science.

[151] van der Beek C.M., Dejong C.H.C., Troost F.J., Masclee A.A.M., Lenaerts K. (2017). Role of Short-Chain Fatty Acids in Colonic Inflammation, Carcinogenesis, and Mucosal Protection and Healing. Nutr. Rev..

[152] Wyatt M., Greathouse K.L. (2021). Targeting Dietary and Microbial Tryptophan-Indole Metabolism as Therapeutic Approaches to Colon Cancer. Nutrients.

[153] Li Q., Cao L., Tian Y., Zhang P., Ding C., Lu W., Jia C., Shao C., Liu W., Wang D. (2018). Butyrate Suppresses the Proliferation of Colorectal Cancer Cells via Targeting Pyruvate Kinase M2 and Metabolic Reprogramming. Mol. Cell Proteom..

[154] O’Keefe S.J.D. (2016). Diet, Microorganisms and Their Metabolites, and Colon Cancer. Nat. Rev. Gastroenterol. Hepatol..

[155] Bachem A., Makhlouf C., Binger K.J., de Souza D.P., Tull D., Hochheiser K., Whitney P.G., Fernandez-Ruiz D., Dähling S., Kastenmüller W. (2019). Microbiota-Derived Short-Chain Fatty Acids Promote the Memory Potential of Antigen-Activated CD8+ T Cells. Immunity.

[156] Hinnebusch B.F., Meng S., Wu J.T., Archer S.Y., Hodin R.A. (2002). The Effects of Short-Chain Fatty Acids on Human Colon Cancer Cell Phenotype Are Associated with Histone Hyperacetylation. J. Nutr..

[157] Burgess D.J. (2012). Metabolism: Warburg behind the Butyrate Paradox?. Nat. Rev. Cancer.

[158] Li L., Sun Y., Liu J., Wu X., Chen L., Ma L., Wu P. (2015). Histone Deacetylase Inhibitor Sodium Butyrate Suppresses DNA Double Strand Break Repair Induced by Etoposide More Effectively in MCF-7 Cells than in HEK293 Cells. BMC Biochem..

[159] Ma Y., Liu X., Wang J. (2022). Small Molecules in the Big Picture of Gut Microbiome-Host Cross-Talk. EBioMedicine.

[160] Mowat C., Dhatt J., Bhatti I., Hamie A., Baker K. (2023). Short Chain Fatty Acids Prime Colorectal Cancer Cells to Activate Antitumor Immunity. Front. Immunol..

[161] Yang Y., Weng W., Peng J., Hong L., Yang L., Toiyama Y., Gao R., Liu M., Yin M., Pan C. (2017). Fusobacterium Nucleatum Increases Proliferation of Colorectal Cancer Cells and Tumor Development in Mice by Activating Toll-Like Receptor 4 Signaling to Nuclear Factor-κB, and Up-Regulating Expression of MicroRNA-21. Gastroenterology.

[162] Stoilov L., Darroudi F., Meschini R., van der Schans G., Mullenders L.H., Natarajan A.T. (2000). Inhibition of Repair of X-Ray-Induced DNA Double-Strand Breaks in Human Lymphocytes Exposed to Sodium Butyrate. Int. J. Radiat. Biol..

[163] Toyooka T., Ibuki Y. (2009). Histone Deacetylase Inhibitor Sodium Butyrate Enhances the Cell Killing Effect of Psoralen plus UVA by Attenuating Nucleotide Excision Repair. Cancer Res..

[164] Koprinarova M., Botev P., Russev G. (2011). Histone Deacetylase Inhibitor Sodium Butyrate Enhances Cellular Radiosensitivity by Inhibiting Both DNA Nonhomologous End Joining and Homologous Recombination. DNA Repair.

[165] Robert T., Vanoli F., Chiolo I., Shubassi G., Bernstein K.A., Rothstein R., Botrugno O.A., Parazzoli D., Oldani A., Minucci S. (2011). HDACs Link the DNA Damage Response, Processing of Double-Strand Breaks and Autophagy. Nature.

[166] Luu M., Riester Z., Baldrich A., Reichardt N., Yuille S., Busetti A., Klein M., Wempe A., Leister H., Raifer H. (2021). Microbial Short-Chain Fatty Acids Modulate CD8+ T Cell Responses and Improve Adoptive Immunotherapy for Cancer. Nat. Commun..

[167] Mowat C., Mosley S.R., Namdar A., Schiller D., Baker K. (2021). Anti-Tumor Immunity in Mismatch Repair-Deficient Colorectal Cancers Requires Type I IFN-Driven CCL5 and CXCL10. J. Exp. Med..

[168] Pedrosa L., Esposito F., Thomson T.M., Maurel J. (2019). The Tumor Microenvironment in Colorectal Cancer Therapy. Cancers.

[169] Park S.L., Gebhardt T., Mackay L.K. (2019). Tissue-Resident Memory T Cells in Cancer Immunosurveillance. Trends Immunol..

[170] Tian Y., Xu Q., Sun L., Ye Y., Ji G. (2018). Short-Chain Fatty Acids Administration Is Protective in Colitis-Associated Colorectal Cancer Development. J. Nutr. Biochem..

[171] Gracie D.J., Hamlin P.J., Ford A.C. (2019). The Influence of the Brain-Gut Axis in Inflammatory Bowel Disease and Possible Implications for Treatment. Lancet Gastroenterol. Hepatol..

[172] Agirman G., Yu K.B., Hsiao E.Y. (2021). Signaling Inflammation across the Gut-Brain Axis. Science.

[173] Margolis K.G., Cryan J.F., Mayer E.A. (2021). The Microbiota-Gut-Brain Axis: From Motility to Mood. Gastroenterology.

[174] Emmi A., Sandre M., Russo F.P., Tombesi G., Garrì F., Campagnolo M., Carecchio M., Biundo R., Spolverato G., Macchi V. (2023). Duodenal Alpha-Synuclein Pathology and Enteric Gliosis in Advanced Parkinson’s Disease. Mov. Disord..

[175] Onyango I.G., Jauregui G.V., Čarná M., Bennett J.P., Stokin G.B. (2021). Neuroinflammation in Alzheimer’s Disease. Biomedicines.

[176] Wang Q., Yang Q., Liu X. (2023). The Microbiota-Gut-Brain Axis and Neurodevelopmental Disorders. Protein Cell.

[177] Wang Z., Wang Z., Lu T., Chen W., Yan W., Yuan K., Shi L., Liu X., Zhou X., Shi J. (2022). The Microbiota-Gut-Brain Axis in Sleep Disorders. Sleep. Med. Rev..

[178] Socała K., Doboszewska U., Szopa A., Serefko A., Włodarczyk M., Zielińska A., Poleszak E., Fichna J., Wlaź P. (2021). The Role of Microbiota-Gut-Brain Axis in Neuropsychiatric and Neurological Disorders. Pharmacol. Res..

[179] Kowalski K., Mulak A. (2019). Brain-Gut-Microbiota Axis in Alzheimer’s Disease. J. Neurogastroenterol. Motil..

[180] Romano S., Savva G.M., Bedarf J.R., Charles I.G., Hildebrand F., Narbad A. (2021). Meta-Analysis of the Parkinson’s Disease Gut Microbiome Suggests Alterations Linked to Intestinal Inflammation. NPJ Park. Dis..

[181] Castelli V., d’Angelo M., Quintiliani M., Benedetti E., Cifone M.G., Cimini A. (2021). The Emerging Role of Probiotics in Neurodegenerative Diseases: New Hope for Parkinson’s Disease?. Neural Regen. Res..

[182] Shannon K.M., Keshavarzian A., Dodiya H.B., Jakate S., Kordower J.H. (2012). Is Alpha-Synuclein in the Colon a Biomarker for Premotor Parkinson’s Disease? Evidence from 3 Cases. Mov. Disord..

[183] Brudek T. (2019). Inflammatory Bowel Diseases and Parkinson’s Disease. J. Park. Dis..

[184] Chen Q.-Q., Haikal C., Li W., Li J.-Y. (2019). Gut Inflammation in Association with Pathogenesis of Parkinson’s Disease. Front. Mol. Neurosci..

[185] Mertsalmi T.H., Pekkonen E., Scheperjans F. (2020). Antibiotic Exposure and Risk of Parkinson’s Disease in Finland: A Nationwide Case-Control Study. Mov. Disord..

[186] Challis C., Hori A., Sampson T.R., Yoo B.B., Challis R.C., Hamilton A.M., Mazmanian S.K., Volpicelli-Daley L.A., Gradinaru V. (2020). Gut-Seeded α-Synuclein Fibrils Promote Gut Dysfunction and Brain Pathology Specifically in Aged Mice. Nat. Neurosci..

[187] Braak H., Del Tredici K., Rüb U., de Vos R.A.I., Jansen Steur E.N.H., Braak E. (2003). Staging of Brain Pathology Related to Sporadic Parkinson’s Disease. Neurobiol. Aging.

[188] Hawkes C.H., Del Tredici K., Braak H. (2010). A Timeline for Parkinson’s Disease. Park. Relat. Disord..

[189] Devos D., Lebouvier T., Lardeux B., Biraud M., Rouaud T., Pouclet H., Coron E., des Varannes S.B., Naveilhan P., Nguyen J.-M. (2013). Colonic Inflammation in Parkinson’s Disease. Neurobiol. Dis..

[190] Chalazonitis A., Rao M. (2018). Enteric Nervous System Manifestations of Neurodegenerative Disease. Brain Res..

[191] Sharon G., Sampson T.R., Geschwind D.H., Mazmanian S.K. (2016). The Central Nervous System and the Gut Microbiome. Cell.

[192] Cryan J.F., Dinan T.G. (2012). Mind-Altering Microorganisms: The Impact of the Gut Microbiota on Brain and Behaviour. Nat. Rev. Neurosci..

[193] Mayer E.A., Knight R., Mazmanian S.K., Cryan J.F., Tillisch K. (2014). Gut Microbes and the Brain: Paradigm Shift in Neuroscience. J. Neurosci..

[194] Knox E.G., Lynch C.M.K., Lee Y.S., O’Driscoll C.M., Clarke G., Cryan J.F., Aburto M.R. (2023). The Gut Microbiota Is Important for the Maintenance of Blood–Cerebrospinal Fluid Barrier Integrity. Eur. J. Neurosci..

[195] Sochocka M., Donskow-Łysoniewska K., Diniz B.S., Kurpas D., Brzozowska E., Leszek J. (2019). The Gut Microbiome Alterations and Inflammation-Driven Pathogenesis of Alzheimer’s Disease-a Critical Review. Mol. Neurobiol..

[196] Lin C.-H., Chen C.-C., Chiang H.-L., Liou J.-M., Chang C.-M., Lu T.-P., Chuang E.Y., Tai Y.-C., Cheng C., Lin H.-Y. (2019). Altered Gut Microbiota and Inflammatory Cytokine Responses in Patients with Parkinson’s Disease. J. Neuroinflamm..

[197] Abdel-Haq R., Schlachetzki J.C.M., Glass C.K., Mazmanian S.K. (2019). Microbiome-Microglia Connections via the Gut-Brain Axis. J. Exp. Med..

[198] Li Z., Liang H., Hu Y., Lu L., Zheng C., Fan Y., Wu B., Zou T., Luo X., Zhang X. (2022). Gut Bacterial Profiles in Parkinson’s Disease: A Systematic Review. CNS Neurosci. Ther..

[199] Trinder M., Daisley B.A., Dube J.S., Reid G. (2017). Drosophila Melanogaster as a High-Throughput Model for Host-Microbiota Interactions. Front. Microbiol..

[200] Ma Q., Xing C., Long W., Wang H.Y., Liu Q., Wang R.-F. (2019). Impact of Microbiota on Central Nervous System and Neurological Diseases: The Gut-Brain Axis. J. Neuroinflamm..

[201] Stilling R.M., Dinan T.G., Cryan J.F. (2014). Microbial Genes, Brain & Behaviour—Epigenetic Regulation of the Gut-Brain Axis. Genes. Brain Behav..

[202] Galland L. (2014). The Gut Microbiome and the Brain. J. Med. Food.

[203] Canfora E.E., Jocken J.W., Blaak E.E. (2015). Short-Chain Fatty Acids in Control of Body Weight and Insulin Sensitivity. Nat. Rev. Endocrinol..

[204] Paul B., Barnes S., Demark-Wahnefried W., Morrow C., Salvador C., Skibola C., Tollefsbol T.O. (2015). Influences of Diet and the Gut Microbiome on Epigenetic Modulation in Cancer and Other Diseases. Clin. Epigenet..

[205] Stilling R.M., van de Wouw M., Clarke G., Stanton C., Dinan T.G., Cryan J.F. (2016). The Neuropharmacology of Butyrate: The Bread and Butter of the Microbiota-Gut-Brain Axis?. Neurochem. Int..

[206] Chen H., Meng L., Shen L. (2022). Multiple Roles of Short-Chain Fatty Acids in Alzheimer Disease. Nutrition.

[207] St Laurent R., O’Brien L.M., Ahmad S.T. (2013). Sodium Butyrate Improves Locomotor Impairment and Early Mortality in a Rotenone-Induced Drosophila Model of Parkinson’s Disease. Neuroscience.

[208] Silva Y.P., Bernardi A., Frozza R.L. (2020). The Role of Short-Chain Fatty Acids from Gut Microbiota in Gut-Brain Communication. Front. Endocrinol..

[209] Unger M.M., Spiegel J., Dillmann K.-U., Grundmann D., Philippeit H., Bürmann J., Faßbender K., Schwiertz A., Schäfer K.-H. (2016). Short Chain Fatty Acids and Gut Microbiota Differ between Patients with Parkinson’s Disease and Age-Matched Controls. Park. Relat. Disord..

[210] Manfready R.A., Forsyth C.B., Voigt R.M., Hall D.A., Goetz C.G., Keshavarzian A. (2022). Gut-Brain Communication in Parkinson’s Disease: Enteroendocrine Regulation by GLP-1. Curr. Neurol. Neurosci. Rep..

[211] Gerhardt S., Mohajeri M.H. (2018). Changes of Colonic Bacterial Composition in Parkinson’s Disease and Other Neurodegenerative Diseases. Nutrients.

[212] Xie A., Ensink E., Li P., Gordevičius J., Marshall L.L., George S., Pospisilik J.A., Aho V.T.E., Houser M.C., Pereira P.A.B. (2022). Bacterial Butyrate in Parkinson’s Disease Is Linked to Epigenetic Changes and Depressive Symptoms. Mov. Disord..

[213] Kong Y., Jiang B., Luo X. (2018). Gut Microbiota Influences Alzheimer’s Disease Pathogenesis by Regulating Acetate in Drosophila Model. Future Microbiol..

[214] Fernandez-Real J.-M., Serino M., Blasco G., Puig J., Daunis-i-Estadella J., Ricart W., Burcelin R., Fernández-Aranda F., Portero-Otin M. (2015). Gut Microbiota Interacts with Brain Microstructure and Function. J. Clin. Endocrinol. Metab..

[215] Müller B., Rasmusson A.J., Just D., Jayarathna S., Moazzami A., Novicic Z.K., Cunningham J.L. (2021). Fecal Short-Chain Fatty Acid Ratios as Related to Gastrointestinal and Depressive Symptoms in Young Adults. Psychosom. Med..

[216] Oddo S., Caccamo A., Shepherd J.D., Murphy M.P., Golde T.E., Kayed R., Metherate R., Mattson M.P., Akbari Y., LaFerla F.M. (2003). Triple-Transgenic Model of Alzheimer’s Disease with Plaques and Tangles: Intracellular Abeta and Synaptic Dysfunction. Neuron.

[217] Abraham D., Feher J., Scuderi G.L., Szabo D., Dobolyi A., Cservenak M., Juhasz J., Ligeti B., Pongor S., Gomez-Cabrera M.C. (2019). Exercise and Probiotics Attenuate the Development of Alzheimer’s Disease in Transgenic Mice: Role of Microbiome. Exp. Gerontol..

[218] Bonfili L., Cecarini V., Cuccioloni M., Angeletti M., Berardi S., Scarpona S., Rossi G., Eleuteri A.M. (2018). SLAB51 Probiotic Formulation Activates SIRT1 Pathway Promoting Antioxidant and Neuroprotective Effects in an AD Mouse Model. Mol. Neurobiol..

[219] Oakley H., Cole S.L., Logan S., Maus E., Shao P., Craft J., Guillozet-Bongaarts A., Ohno M., Disterhoft J., Van Eldik L. (2006). Intraneuronal Beta-Amyloid Aggregates, Neurodegeneration, and Neuron Loss in Transgenic Mice with Five Familial Alzheimer’s Disease Mutations: Potential Factors in Amyloid Plaque Formation. J. Neurosci..

[220] Liu Q., Xi Y., Wang Q., Liu J., Li P., Meng X., Liu K., Chen W., Liu X., Liu Z. (2021). Mannan Oligosaccharide Attenuates Cognitive and Behavioral Disorders in the 5xFAD Alzheimer’s Disease Mouse Model via Regulating the Gut Microbiota-Brain Axis. Brain Behav. Immun..

[221] Park S.-H., Lee J.H., Shin J., Kim J.-S., Cha B., Lee S., Kwon K.S., Shin Y.W., Choi S.H. (2021). Cognitive Function Improvement after Fecal Microbiota Transplantation in Alzheimer’s Dementia Patient: A Case Report. Curr. Med. Res. Opin..

[222] Ibrahim A., Ali R.A.R., Manaf M.R.A., Ahmad N., Tajurruddin F.W., Qin W.Z., Desa S.H.M., Ibrahim N.M. (2020). Multi-Strain Probiotics (Hexbio) Containing MCP BCMC Strains Improved Constipation and Gut Motility in Parkinson’s Disease: A Randomised Controlled Trial. PLoS ONE.

[223] Georgescu D., Ancusa O.E., Georgescu L.A., Ionita I., Reisz D. (2016). Nonmotor Gastrointestinal Disorders in Older Patients with Parkinson’s Disease: Is There Hope?. Clin. Interv. Aging.

[224] Fang X., Zhou X., Miao Y., Han Y., Wei J., Chen T. (2020). Therapeutic Effect of GLP-1 Engineered Strain on Mice Model of Alzheimer’s Disease and Parkinson’s Disease. AMB Express.

[225] Cassani E., Privitera G., Pezzoli G., Pusani C., Madio C., Iorio L., Barichella M. (2011). Use of Probiotics for the Treatment of Constipation in Parkinson’s Disease Patients. Minerva Gastroenterol. Dietol..

[226] Barichella M., Pacchetti C., Bolliri C., Cassani E., Iorio L., Pusani C., Pinelli G., Privitera G., Cesari I., Faierman S.A. (2016). Probiotics and Prebiotic Fiber for Constipation Associated with Parkinson Disease: An RCT. Neurology.

[227] Tamtaji O.R., Taghizadeh M., Kakhaki R.D., Kouchaki E., Bahmani F., Borzabadi S., Oryan S., Mafi A., Asemi Z. (2019). Clinical and Metabolic Response to Probiotic Administration in People with Parkinson’s Disease: A Randomized, Double-Blind, Placebo-Controlled Trial. Clin. Nutr..

[228] Tan A.H., Lim S.-Y., Chong K.K., A Manap M.A.A., Hor J.W., Lim J.L., Low S.C., Chong C.W., Mahadeva S., Lang A.E. (2021). Probiotics for Constipation in Parkinson Disease: A Randomized Placebo-Controlled Study. Neurology.

[229] DuPont H.L., Suescun J., Jiang Z.-D., Brown E.L., Essigmann H.T., Alexander A.S., DuPont A.W., Iqbal T., Utay N.S., Newmark M. (2023). Fecal Microbiota Transplantation in Parkinson’s Disease—A Randomized Repeat-Dose, Placebo-Controlled Clinical Pilot Study. Front. Neurol..

[230] Li T., Chu C., Yu L., Zhai Q., Wang S., Zhao J., Zhang H., Chen W., Tian F. (2022). Neuroprotective Effects of Bifidobacterium Breve CCFM1067 in MPTP-Induced Mouse Models of Parkinson’s Disease. Nutrients.

[231] Abdel-Haq R., Schlachetzki J.C.M., Boktor J.C., Cantu-Jungles T.M., Thron T., Zhang M., Bostick J.W., Khazaei T., Chilakala S., Morais L.H. (2022). A Prebiotic Diet Modulates Microglial States and Motor Deficits in α-Synuclein Overexpressing Mice. Elife.

[232] Liu X., Du Z.R., Wang X., Sun X.R., Zhao Q., Zhao F., Wong W.T., Wong K.H., Dong X.-L. (2022). Polymannuronic Acid Prebiotic plus Lacticaseibacillus Rhamnosus GG Probiotic as a Novel Synbiotic Promoted Their Separate Neuroprotection against Parkinson’s Disease. Food Res. Int..

[233] Sun H., Zhao F., Liu Y., Ma T., Jin H., Quan K., Leng B., Zhao J., Yuan X., Li Z. (2022). Probiotics Synergized with Conventional Regimen in Managing Parkinson’s Disease. NPJ Park. Dis..

[234] Lacy B.E., Patel N.K. (2017). Rome Criteria and a Diagnostic Approach to Irritable Bowel Syndrome. J. Clin. Med..

[235] Barbara G., Cremon C., Bellini M., Corsetti M., Di Nardo G., Falangone F., Fuccio L., Galeazzi F., Iovino P., Sarnelli G. (2023). Italian Guidelines for the Management of Irritable Bowel Syndrome: Joint Consensus from the Italian Societies of: Gastroenterology and Endoscopy (SIGE), Neurogastroenterology and Motility (SINGEM), Hospital Gastroenterologists and Endoscopists (AIGO), Digestive Endoscopy (SIED), General Medicine (SIMG), Gastroenterology, Hepatology and Pediatric Nutrition (SIGENP) and Pediatrics (SIP). Dig. Liver Dis..

[236] Savarino E., Zingone F., Barberio B., Marasco G., Akyuz F., Akpinar H., Barboi O., Bodini G., Bor S., Chiarioni G. (2022). Functional Bowel Disorders with Diarrhoea: Clinical Guidelines of the United European Gastroenterology and European Society for Neurogastroenterology and Motility. United Eur. Gastroenterol. J..

[237] Tana C., Umesaki Y., Imaoka A., Handa T., Kanazawa M., Fukudo S. (2010). Altered Profiles of Intestinal Microbiota and Organic Acids May Be the Origin of Symptoms in Irritable Bowel Syndrome. Neurogastroenterol. Motil..

[238] Sun Q., Jia Q., Song L., Duan L. (2019). Alterations in Fecal Short-Chain Fatty Acids in Patients with Irritable Bowel Syndrome. Medicine.

[239] Gargari G., Taverniti V., Gardana C., Cremon C., Canducci F., Pagano I., Barbaro M.R., Bellacosa L., Castellazzi A.M., Valsecchi C. (2018). Fecal Clostridiales Distribution and Short-Chain Fatty Acids Reflect Bowel Habits in Irritable Bowel Syndrome. Environ. Microbiol..

[240] Treem W.R., Ahsan N., Kastoff G., Hyams J.S. (1996). Fecal Short-Chain Fatty Acids in Patients with Diarrhea-Predominant Irritable Bowel Syndrome: In Vitro Studies of Carbohydrate Fermentation. J. Pediatr. Gastroenterol. Nutr..

[241] Fredericks E., Theunissen R., Roux S. (2020). Short Chain Fatty Acids and Monocarboxylate Transporters in Irritable Bowel Syndrome. Turk. J. Gastroenterol..

[242] Undseth R., Jakobsdottir G., Nyman M., Berstad A., Valeur J. (2015). Low Serum Levels of Short-Chain Fatty Acids after Lactulose Ingestion May Indicate Impaired Colonic Fermentation in Patients with Irritable Bowel Syndrome. Clin. Exp. Gastroenterol..

[243] Chassard C., Dapoigny M., Scott K.P., Crouzet L., Del’homme C., Marquet P., Martin J.C., Pickering G., Ardid D., Eschalier A. (2012). Functional Dysbiosis within the Gut Microbiota of Patients with Constipated-Irritable Bowel Syndrome. Aliment. Pharmacol. Ther..

[244] Pozuelo M., Panda S., Santiago A., Mendez S., Accarino A., Santos J., Guarner F., Azpiroz F., Manichanh C. (2015). Reduction of Butyrate- and Methane-Producing Microorganisms in Patients with Irritable Bowel Syndrome. Sci. Rep..

[245] Zhou J., Wei H., Zhou A., Xiao X., Xie X., Tang B., Lin H., Tang L., Meng R., Yuan X. (2024). The Gut Microbiota Participates in the Effect of Linaclotide in Patients with Irritable Bowel Syndrome with Constipation (IBS-C): A Multicenter, Prospective, Pre-Post Study. J. Transl. Med..

[246] Farup P.G., Rudi K., Hestad K. (2016). Faecal Short-Chain Fatty Acids—A Diagnostic Biomarker for Irritable Bowel Syndrome?. BMC Gastroenterol..

[247] Macia L., Tan J., Vieira A.T., Leach K., Stanley D., Luong S., Maruya M., Ian McKenzie C., Hijikata A., Wong C. (2015). Metabolite-Sensing Receptors GPR43 and GPR109A Facilitate Dietary Fibre-Induced Gut Homeostasis through Regulation of the Inflammasome. Nat. Commun..

[248] Kim M.H., Kang S.G., Park J.H., Yanagisawa M., Kim C.H. (2013). Short-Chain Fatty Acids Activate GPR41 and GPR43 on Intestinal Epithelial Cells to Promote Inflammatory Responses in Mice. Gastroenterology.

[249] Zaki M.H., Boyd K.L., Vogel P., Kastan M.B., Lamkanfi M., Kanneganti T.-D. (2010). The NLRP3 Inflammasome Protects against Loss of Epithelial Integrity and Mortality during Experimental Colitis. Immunity.

[250] Hirota S.A., Ng J., Lueng A., Khajah M., Parhar K., Li Y., Lam V., Potentier M.S., Ng K., Bawa M. (2011). NLRP3 Inflammasome Plays a Key Role in the Regulation of Intestinal Homeostasis. Inflamm. Bowel Dis..

[251] Dunsmore G., Koleva P., Ghobakhloo N., Sutton R., Ambrosio L., Meng X., Hotte N., Nguyen V., Madsen K.L., Dieleman L.A. (2019). Lower Abundance and Impaired Function of CD71+ Erythroid Cells in Inflammatory Bowel Disease Patients during Pregnancy. J. Crohns Colitis.

[252] Sun M., Wu W., Chen L., Yang W., Huang X., Ma C., Chen F., Xiao Y., Zhao Y., Ma C. (2018). Microbiota-Derived Short-Chain Fatty Acids Promote Th1 Cell IL-10 Production to Maintain Intestinal Homeostasis. Nat. Commun..

[253] Burger-van Paassen N., Vincent A., Puiman P.J., van der Sluis M., Bouma J., Boehm G., van Goudoever J.B., van Seuningen I., Renes I.B. (2009). The Regulation of Intestinal Mucin MUC2 Expression by Short-Chain Fatty Acids: Implications for Epithelial Protection. Biochem. J..

[254] Hatayama H., Iwashita J., Kuwajima A., Abe T. (2007). The Short Chain Fatty Acid, Butyrate, Stimulates MUC2 Mucin Production in the Human Colon Cancer Cell Line, LS174T. Biochem. Biophys. Res. Commun..

[255] Augenlicht L., Shi L., Mariadason J., Laboisse C., Velcich A. (2003). Repression of MUC2 Gene Expression by Butyrate, a Physiological Regulator of Intestinal Cell Maturation. Oncogene.

[256] Willemsen L.E.M., Koetsier M.A., van Deventer S.J.H., van Tol E.A.F. (2003). Short Chain Fatty Acids Stimulate Epithelial Mucin 2 Expression through Differential Effects on Prostaglandin E(1) and E(2) Production by Intestinal Myofibroblasts. Gut.

[257] Finnie I.A., Dwarakanath A.D., Taylor B.A., Rhodes J.M. (1995). Colonic Mucin Synthesis Is Increased by Sodium Butyrate. Gut.

[258] Miao W., Wu X., Wang K., Wang W., Wang Y., Li Z., Liu J., Li L., Peng L. (2016). Sodium Butyrate Promotes Reassembly of Tight Junctions in Caco-2 Monolayers Involving Inhibition of MLCK/MLC2 Pathway and Phosphorylation of PKCβ2. Int. J. Mol. Sci..

[259] Jin B., Ha S.E., Wei L., Singh R., Zogg H., Clemmensen B., Heredia D.J., Gould T.W., Sanders K.M., Ro S. (2021). Colonic Motility Is Improved by the Activation of 5-HT2B Receptors on Interstitial Cells of Cajal in Diabetic Mice. Gastroenterology.

[260] Tharayil V.S., Wouters M.M., Stanich J.E., Roeder J.L., Lei S., Beyder A., Gomez-Pinilla P.J., Gershon M.D., Maroteaux L., Gibbons S.J. (2010). Lack of Serotonin 5-HT2B Receptor Alters Proliferation and Network Volume of Interstitial Cells of Cajal In Vivo. Neurogastroenterol. Motil..

[261] Wouters M.M., Gibbons S.J., Roeder J.L., Distad M., Ou Y., Strege P.R., Szurszewski J.H., Farrugia G. (2007). Exogenous Serotonin Regulates Proliferation of Interstitial Cells of Cajal in Mouse Jejunum through 5-HT2B Receptors. Gastroenterology.

[262] Ono S., Karaki S., Kuwahara A. (2004). Short-Chain Fatty Acids Decrease the Frequency of Spontaneous Contractions of Longitudinal Muscle via Enteric Nerves in Rat Distal Colon. Jpn. J. Physiol..

[263] Grider J.R., Kuemmerle J.F., Jin J.G. (1996). 5-HT Released by Mucosal Stimuli Initiates Peristalsis by Activating 5-HT4/5-HT1p Receptors on Sensory CGRP Neurons. Am. J. Physiol..

[264] Foxx-Orenstein A.E., Kuemmerle J.F., Grider J.R. (1996). Distinct 5-HT Receptors Mediate the Peristaltic Reflex Induced by Mucosal Stimuli in Human and Guinea Pig Intestine. Gastroenterology.

[265] Vicentini F.A., Keenan C.M., Wallace L.E., Woods C., Cavin J.-B., Flockton A.R., Macklin W.B., Belkind-Gerson J., Hirota S.A., Sharkey K.A. (2021). Intestinal Microbiota Shapes Gut Physiology and Regulates Enteric Neurons and Glia. Microbiome.

[266] Soret R., Chevalier J., De Coppet P., Poupeau G., Derkinderen P., Segain J.P., Neunlist M. (2010). Short-Chain Fatty Acids Regulate the Enteric Neurons and Control Gastrointestinal Motility in Rats. Gastroenterology.

[267] Suply E., de Vries P., Soret R., Cossais F., Neunlist M. (2012). Butyrate Enemas Enhance Both Cholinergic and Nitrergic Phenotype of Myenteric Neurons and Neuromuscular Transmission in Newborn Rat Colon. Am. J. Physiol. Gastrointest. Liver Physiol..

[268] Shaidullov I.F., Sorokina D.M., Sitdikov F.G., Hermann A., Abdulkhakov S.R., Sitdikova G.F. (2021). Short Chain Fatty Acids and Colon Motility in a Mouse Model of Irritable Bowel Syndrome. BMC Gastroenterol..

[269] Yuan F., Tan W., Ren H., Yan L., Wang Y., Luo H. (2020). The Effects of Short-Chain Fatty Acids on Rat Colonic Hypermotility Induced by Water Avoidance Stress. Drug Des. Devel Ther..

[270] Hurst N.R., Kendig D.M., Murthy K.S., Grider J.R. (2014). The Short Chain Fatty Acids, Butyrate and Propionate, Have Differential Effects on the Motility of the Guinea Pig Colon. Neurogastroenterol. Motil..

[271] Waseem M.R., Shin A., Siwiec R., James-Stevenson T., Bohm M., Rogers N., Wo J., Waseem L., Gupta A., Jarrett M. (2023). Associations of Fecal Short Chain Fatty Acids with Colonic Transit, Fecal Bile Acid, and Food Intake in Irritable Bowel Syndrome. Clin. Transl. Gastroenterol..

[272] Day E.A., Ford R.J., Steinberg G.R. (2017). AMPK as a Therapeutic Target for Treating Metabolic Diseases. Trends Endocrinol. Metab..

[273] Dabke K., Hendrick G., Devkota S. (2019). The Gut Microbiome and Metabolic Syndrome. J. Clin. Investig..

[274] Rinella M.E., Lazarus J.V., Ratziu V., Francque S.M., Sanyal A.J., Kanwal F., Romero D., Abdelmalek M.F., Anstee Q.M., Arab J.P. (2023). A Multisociety Delphi Consensus Statement on New Fatty Liver Disease Nomenclature. J. Hepatol..

[275] Fan Y., Pedersen O. (2021). Gut Microbiota in Human Metabolic Health and Disease. Nat. Rev. Microbiol..

[276] Waalen J. (2014). The Genetics of Human Obesity. Transl. Res..

[277] Murugesan S., Ulloa-Martínez M., Martínez-Rojano H., Galván-Rodríguez F.M., Miranda-Brito C., Romano M.C., Piña-Escobedo A., Pizano-Zárate M.L., Hoyo-Vadillo C., García-Mena J. (2015). Study of the Diversity and Short-Chain Fatty Acids Production by the Bacterial Community in Overweight and Obese Mexican Children. Eur. J. Clin. Microbiol. Infect. Dis..

[278] Ecklu-Mensah G., Choo-Kang C., Maseng M.G., Donato S., Bovet P., Viswanathan B., Bedu-Addo K., Plange-Rhule J., Oti Boateng P., Forrester T.E. (2023). Gut Microbiota and Fecal Short Chain Fatty Acids Differ with Adiposity and Country of Origin: The METS-Microbiome Study. Nat. Commun..

[279] Schwiertz A., Taras D., Schäfer K., Beijer S., Bos N.A., Donus C., Hardt P.D. (2010). Microbiota and SCFA in Lean and Overweight Healthy Subjects. Obesity.

[280] Fernandes J., Su W., Rahat-Rozenbloom S., Wolever T.M.S., Comelli E.M. (2014). Adiposity, Gut Microbiota and Faecal Short Chain Fatty Acids Are Linked in Adult Humans. Nutr. Diabetes.

[281] De la Cuesta-Zuluaga J., Mueller N.T., Álvarez-Quintero R., Velásquez-Mejía E.P., Sierra J.A., Corrales-Agudelo V., Carmona J.A., Abad J.M., Escobar J.S. (2018). Higher Fecal Short-Chain Fatty Acid Levels Are Associated with Gut Microbiome Dysbiosis, Obesity, Hypertension and Cardiometabolic Disease Risk Factors. Nutrients.

[282] Valdes A.M., Walter J., Segal E., Spector T.D. (2018). Role of the Gut Microbiota in Nutrition and Health. BMJ.

[283] Tirosh A., Calay E.S., Tuncman G., Claiborn K.C., Inouye K.E., Eguchi K., Alcala M., Rathaus M., Hollander K.S., Ron I. (2019). The Short-Chain Fatty Acid Propionate Increases Glucagon and FABP4 Production, Impairing Insulin Action in Mice and Humans. Sci. Transl. Med..

[284] Perry R.J., Peng L., Barry N.A., Cline G.W., Zhang D., Cardone R.L., Petersen K.F., Kibbey R.G., Goodman A.L., Shulman G.I. (2016). Acetate Mediates a Microbiome-Brain-β Cell Axis Promoting Metabolic Syndrome. Nature.

[285] Teixeira T.F.S., Grześkowiak Ł., Franceschini S.C.C., Bressan J., Ferreira C.L.L.F., Peluzio M.C.G. (2013). Higher Level of Faecal SCFA in Women Correlates with Metabolic Syndrome Risk Factors. Br. J. Nutr..

[286] Royall D., Wolever T.M., Jeejeebhoy K.N. (1990). Clinical Significance of Colonic Fermentation. Am. J. Gastroenterol..

[287] Reshef L., Niv J., Shapiro B. (1967). Effect of Propionate on Lipogenesis in Adipose Tissue. J. Lipid Res..

[288] Wolever T.M., Brighenti F., Royall D., Jenkins A.L., Jenkins D.J. (1989). Effect of Rectal Infusion of Short Chain Fatty Acids in Human Subjects. Am. J. Gastroenterol..

[289] Lin H.V., Frassetto A., Kowalik E.J., Nawrocki A.R., Lu M.M., Kosinski J.R., Hubert J.A., Szeto D., Yao X., Forrest G. (2012). Butyrate and Propionate Protect against Diet-Induced Obesity and Regulate Gut Hormones via Free Fatty Acid Receptor 3-Independent Mechanisms. PLoS ONE.

[290] Frost G., Sleeth M.L., Sahuri-Arisoylu M., Lizarbe B., Cerdan S., Brody L., Anastasovska J., Ghourab S., Hankir M., Zhang S. (2014). The Short-Chain Fatty Acid Acetate Reduces Appetite via a Central Homeostatic Mechanism. Nat. Commun..

[291] Gao Z., Yin J., Zhang J., Ward R.E., Martin R.J., Lefevre M., Cefalu W.T., Ye J. (2009). Butyrate Improves Insulin Sensitivity and Increases Energy Expenditure in Mice. Diabetes.

[292] Lu Y., Fan C., Li P., Lu Y., Chang X., Qi K. (2016). Short Chain Fatty Acids Prevent High-Fat-Diet-Induced Obesity in Mice by Regulating G Protein-Coupled Receptors and Gut Microbiota. Sci. Rep..

[293] Bishop K.S., Kao C.H.J., Xu Y., Glucina M.P., Paterson R.R.M., Ferguson L.R. (2015). From 2000 years of Ganoderma Lucidum to Recent Developments in Nutraceuticals. Phytochemistry.

[294] Sang T., Guo C., Guo D., Wu J., Wang Y., Wang Y., Chen J., Chen C., Wu K., Na K. (2021). Suppression of Obesity and Inflammation by Polysaccharide from Sporoderm-Broken Spore of Ganoderma Lucidum via Gut Microbiota Regulation. Carbohydr. Polym..

[295] Forslund K., Hildebrand F., Nielsen T., Falony G., Le Chatelier E., Sunagawa S., Prifti E., Vieira-Silva S., Gudmundsdottir V., Pedersen H.K. (2015). Disentangling Type 2 Diabetes and Metformin Treatment Signatures in the Human Gut Microbiota. Nature.

[296] Jocken J.W.E., González Hernández M.A., Hoebers N.T.H., van der Beek C.M., Essers Y.P.G., Blaak E.E., Canfora E.E. (2017). Short-Chain Fatty Acids Differentially Affect Intracellular Lipolysis in a Human White Adipocyte Model. Front. Endocrinol..

[297] Christiansen C.B., Gabe M.B.N., Svendsen B., Dragsted L.O., Rosenkilde M.M., Holst J.J. (2018). The Impact of Short-Chain Fatty Acids on GLP-1 and PYY Secretion from the Isolated Perfused Rat Colon. Am. J. Physiol. Gastrointest. Liver Physiol..

[298] Veprik A., Laufer D., Weiss S., Rubins N., Walker M.D. (2016). GPR41 Modulates Insulin Secretion and Gene Expression in Pancreatic β-Cells and Modifies Metabolic Homeostasis in Fed and Fasting States. FASEB J..

[299] Priyadarshini M., Villa S.R., Fuller M., Wicksteed B., Mackay C.R., Alquier T., Poitout V., Mancebo H., Mirmira R.G., Gilchrist A. (2015). An Acetate-Specific GPCR, FFAR2, Regulates Insulin Secretion. Mol. Endocrinol..

[300] Fushimi T., Tayama K., Fukaya M., Kitakoshi K., Nakai N., Tsukamoto Y., Sato Y. (2001). Acetic Acid Feeding Enhances Glycogen Repletion in Liver and Skeletal Muscle of Rats. J. Nutr..

[301] Li H., Gao Z., Zhang J., Ye X., Xu A., Ye J., Jia W. (2012). Sodium Butyrate Stimulates Expression of Fibroblast Growth Factor 21 in Liver by Inhibition of Histone Deacetylase 3. Diabetes.

[302] Li X., Chen H., Guan Y., Li X., Lei L., Liu J., Yin L., Liu G., Wang Z. (2013). Acetic Acid Activates the AMP-Activated Protein Kinase Signaling Pathway to Regulate Lipid Metabolism in Bovine Hepatocytes. PLoS ONE.

[303] He J., Zhang P., Shen L., Niu L., Tan Y., Chen L., Zhao Y., Bai L., Hao X., Li X. (2020). Short-Chain Fatty Acids and Their Association with Signalling Pathways in Inflammation, Glucose and Lipid Metabolism. Int. J. Mol. Sci..

[304] Yamashita H., Maruta H., Jozuka M., Kimura R., Iwabuchi H., Yamato M., Saito T., Fujisawa K., Takahashi Y., Kimoto M. (2009). Effects of Acetate on Lipid Metabolism in Muscles and Adipose Tissues of Type 2 Diabetic Otsuka Long-Evans Tokushima Fatty (OLETF) Rats. Biosci. Biotechnol. Biochem..

[305] Yamashita H. (2016). Biological Function of Acetic Acid-Improvement in Obesity and Glucose Tolerance by Acetic Acid in Type 2 Diabetic Rats. Crit. Rev. Food Sci. Nutr..

[306] De Vadder F., Kovatcheva-Datchary P., Goncalves D., Vinera J., Zitoun C., Duchampt A., Bäckhed F., Mithieux G. (2014). Microbiota-Generated Metabolites Promote Metabolic Benefits via Gut-Brain Neural Circuits. Cell.

[307] Vily-Petit J., Soty M., Silva M., Micoud M., Bron C., Guérin-Deremaux L., Mithieux G. (2023). Improvement of Energy Metabolism Associated with NUTRIOSE^®^ Soluble Fiber, a Dietary Ingredient Exhibiting Prebiotic Properties, Requires Intestinal Gluconeogenesis. Food Res. Int..

[308] Liu L., Fu C., Li F. (2019). Acetate Affects the Process of Lipid Metabolism in Rabbit Liver, Skeletal Muscle and Adipose Tissue. Animals.

[309] Zhao L., Lou H., Peng Y., Chen S., Zhang Y., Li X. (2019). Comprehensive Relationships between Gut Microbiome and Faecal Metabolome in Individuals with Type 2 Diabetes and Its Complications. Endocrine.

[310] Pedersen H.K., Gudmundsdottir V., Nielsen H.B., Hyotylainen T., Nielsen T., Jensen B.A.H., Forslund K., Hildebrand F., Prifti E., Falony G. (2016). Human Gut Microbes Impact Host Serum Metabolome and Insulin Sensitivity. Nature.

[311] Perera D., Kleinstein S.E., Hanson B., Hasturk H., Eveloff R., Freire M., Ramsey M. (2021). Impaired Host Response and the Presence of Acinetobacter Baumannii in the Serum Microbiome of Type-II Diabetic Patients. iScience.

[312] Mariño E., Richards J.L., McLeod K.H., Stanley D., Yap Y.A., Knight J., McKenzie C., Kranich J., Oliveira A.C., Rossello F.J. (2017). Gut Microbial Metabolites Limit the Frequency of Autoimmune T Cells and Protect against Type 1 Diabetes. Nat. Immunol..

[313] Sanna S., van Zuydam N.R., Mahajan A., Kurilshikov A., Vich Vila A., Võsa U., Mujagic Z., Masclee A.A.M., Jonkers D.M.A.E., Oosting M. (2019). Causal Relationships among the Gut Microbiome, Short-Chain Fatty Acids and Metabolic Diseases. Nat. Genet..

[314] Ayesha I.E., Monson N.R., Klair N., Patel U., Saxena A., Patel D., Venugopal S. (2023). Probiotics and Their Role in the Management of Type 2 Diabetes Mellitus (Short-Term Versus Long-Term Effect): A Systematic Review and Meta-Analysis. Cureus.

[315] Byrne C.S., Chambers E.S., Preston T., Tedford C., Brignardello J., Garcia-Perez I., Holmes E., Wallis G.A., Morrison D.J., Frost G.S. (2019). Effects of Inulin Propionate Ester Incorporated into Palatable Food Products on Appetite and Resting Energy Expenditure: A Randomised Crossover Study. Nutrients.

[316] Bouter K., Bakker G.J., Levin E., Hartstra A.V., Kootte R.S., Udayappan S.D., Katiraei S., Bahler L., Gilijamse P.W., Tremaroli V. (2018). Differential Metabolic Effects of Oral Butyrate Treatment in Lean versus Metabolic Syndrome Subjects. Clin. Transl. Gastroenterol..

[317] Vrieze A., Van Nood E., Holleman F., Salojärvi J., Kootte R.S., Bartelsman J.F.W.M., Dallinga-Thie G.M., Ackermans M.T., Serlie M.J., Oozeer R. (2012). Transfer of Intestinal Microbiota from Lean Donors Increases Insulin Sensitivity in Individuals with Metabolic Syndrome. Gastroenterology.

[318] Komaroff A.L. (2017). The Microbiome and Risk for Obesity and Diabetes. JAMA.

[319] Xu H., Li X., Adams H., Kubena K., Guo S. (2018). Etiology of Metabolic Syndrome and Dietary Intervention. Int. J. Mol. Sci..

[320] Mouzaki M., Comelli E.M., Arendt B.M., Bonengel J., Fung S.K., Fischer S.E., McGilvray I.D., Allard J.P. (2013). Intestinal Microbiota in Patients with Nonalcoholic Fatty Liver Disease. Hepatology.

[321] Ding Y., Yanagi K., Cheng C., Alaniz R.C., Lee K., Jayaraman A. (2019). Interactions between Gut Microbiota and Non-Alcoholic Liver Disease: The Role of Microbiota-Derived Metabolites. Pharmacol. Res..

[322] Schoeler M., Caesar R. (2019). Dietary Lipids, Gut Microbiota and Lipid Metabolism. Rev. Endocr. Metab. Disord..

[323] Xie C., Halegoua-DeMarzio D. (2019). Role of Probiotics in Non-Alcoholic Fatty Liver Disease: Does Gut Microbiota Matter?. Nutrients.

[324] Zhang X., Coker O.O., Chu E.S., Fu K., Lau H.C.H., Wang Y.-X., Chan A.W.H., Wei H., Yang X., Sung J.J.Y. (2021). Dietary Cholesterol Drives Fatty Liver-Associated Liver Cancer by Modulating Gut Microbiota and Metabolites. Gut.

[325] Rau M., Rehman A., Dittrich M., Groen A.K., Hermanns H.M., Seyfried F., Beyersdorf N., Dandekar T., Rosenstiel P., Geier A. (2018). Fecal SCFAs and SCFA-Producing Bacteria in Gut Microbiome of Human NAFLD as a Putative Link to Systemic T-Cell Activation and Advanced Disease. United Eur. Gastroenterol. J..

[326] Aragonès G., Colom-Pellicer M., Aguilar C., Guiu-Jurado E., Martínez S., Sabench F., Porras J.A., Riesco D., Del Castillo D., Richart C. (2020). Circulating Microbiota-Derived Metabolites: A “liquid Biopsy?. Int. J. Obes..

[327] Adler G.K., Hornik E.S., Murray G., Bhandari S., Yadav Y., Heydarpour M., Basu R., Garg R., Tirosh A. (2021). Acute Effects of the Food Preservative Propionic Acid on Glucose Metabolism in Humans. BMJ Open Diabetes Res. Care.

[328] Xiong J., Chen X., Zhao Z., Liao Y., Zhou T., Xiang Q. (2022). A Potential Link between Plasma Short-Chain Fatty Acids, TNF-α Level and Disease Progression in Non-Alcoholic Fatty Liver Disease: A Retrospective Study. Exp. Ther. Med..

[329] Behary J., Amorim N., Jiang X.-T., Raposo A., Gong L., McGovern E., Ibrahim R., Chu F., Stephens C., Jebeili H. (2021). Gut Microbiota Impact on the Peripheral Immune Response in Non-Alcoholic Fatty Liver Disease Related Hepatocellular Carcinoma. Nat. Commun..

[330] Tsai H.-J., Hung W.-C., Hung W.-W., Lee Y.-J., Chen Y.-C., Lee C.-Y., Tsai Y.-C., Dai C.-Y. (2023). Circulating Short-Chain Fatty Acids and Non-Alcoholic Fatty Liver Disease Severity in Patients with Type 2 Diabetes Mellitus. Nutrients.

[331] Morrison D.J., Preston T. (2016). Formation of Short Chain Fatty Acids by the Gut Microbiota and Their Impact on Human Metabolism. Gut Microbes.

[332] Albillos A., de Gottardi A., Rescigno M. (2020). The Gut-Liver Axis in Liver Disease: Pathophysiological Basis for Therapy. J. Hepatol..

[333] Nogal A., Louca P., Zhang X., Wells P.M., Steves C.J., Spector T.D., Falchi M., Valdes A.M., Menni C. (2021). Circulating Levels of the Short-Chain Fatty Acid Acetate Mediate the Effect of the Gut Microbiome on Visceral Fat. Front. Microbiol..

[334] Loomba R., Seguritan V., Li W., Long T., Klitgord N., Bhatt A., Dulai P.S., Caussy C., Bettencourt R., Highlander S.K. (2017). Gut Microbiome-Based Metagenomic Signature for Non-Invasive Detection of Advanced Fibrosis in Human Nonalcoholic Fatty Liver Disease. Cell Metab..

[335] Thing M., Werge M.P., Kimer N., Hetland L.E., Rashu E.B., Nabilou P., Junker A.E., Galsgaard E.D., Bendtsen F., Laupsa-Borge J. (2024). Targeted Metabolomics Reveals Plasma Short-Chain Fatty Acids Are Associated with Metabolic Dysfunction-Associated Steatotic Liver Disease. BMC Gastroenterol..

[336] Dangana E.O., Omolekulo T.E., Areola E.D., Olaniyi K.S., Soladoye A.O., Olatunji L.A. (2020). Sodium Acetate Protects against Nicotine-Induced Excess Hepatic Lipid in Male Rats by Suppressing Xanthine Oxidase Activity. Chem. Biol. Interact..

[337] Jin C.J., Sellmann C., Engstler A.J., Ziegenhardt D., Bergheim I. (2015). Supplementation of Sodium Butyrate Protects Mice from the Development of Non-Alcoholic Steatohepatitis (NASH). Br. J. Nutr..

[338] Zhou D., Fan J.-G. (2019). Microbial Metabolites in Non-Alcoholic Fatty Liver Disease. World J. Gastroenterol..

[339] Deng M., Qu F., Chen L., Liu C., Zhang M., Ren F., Guo H., Zhang H., Ge S., Wu C. (2020). SCFAs Alleviated Steatosis and Inflammation in Mice with NASH Induced by MCD. J. Endocrinol..

[340] Zhao Z.-H., Wang Z.-X., Zhou D., Han Y., Ma F., Hu Z., Xin F.-Z., Liu X.-L., Ren T.-Y., Zhang F. (2021). Sodium Butyrate Supplementation Inhibits Hepatic Steatosis by Stimulating Liver Kinase B1 and Insulin-Induced Gene. Cell Mol. Gastroenterol. Hepatol..

[341] Zhou D., Pan Q., Xin F.-Z., Zhang R.-N., He C.-X., Chen G.-Y., Liu C., Chen Y.-W., Fan J.-G. (2017). Sodium Butyrate Attenuates High-Fat Diet-Induced Steatohepatitis in Mice by Improving Gut Microbiota and Gastrointestinal Barrier. World J. Gastroenterol..

[342] Liu W., Luo X., Tang J., Mo Q., Zhong H., Zhang H., Feng F. (2021). A Bridge for Short-Chain Fatty Acids to Affect Inflammatory Bowel Disease, Type 1 Diabetes, and Non-Alcoholic Fatty Liver Disease Positively: By Changing Gut Barrier. Eur. J. Nutr..

[343] Leonardi I., Paramsothy S., Doron I., Semon A., Kaakoush N.O., Clemente J.C., Faith J.J., Borody T.J., Mitchell H.M., Colombel J.-F. (2020). Fungal Trans-Kingdom Dynamics Linked to Responsiveness to Fecal Microbiota Transplantation (FMT) Therapy in Ulcerative Colitis. Cell Host Microbe.

[344] de Groot P., Scheithauer T., Bakker G.J., Prodan A., Levin E., Khan M.T., Herrema H., Ackermans M., Serlie M.J.M., de Brauw M. (2020). Donor Metabolic Characteristics Drive Effects of Faecal Microbiota Transplantation on Recipient Insulin Sensitivity, Energy Expenditure and Intestinal Transit Time. Gut.

[345] Peery A.F., Kelly C.R., Kao D., Vaughn B.P., Lebwohl B., Singh S., Imdad A., Altayar O. (2024). AGA Clinical Practice Guideline on Fecal Microbiota–Based Therapies for Select Gastrointestinal Diseases. Gastroenterology.

[346] Mullish B.H., Quraishi M.N., Segal J.P., McCune V.L., Baxter M., Marsden G.L., Moore D.J., Colville A., Bhala N., Iqbal T.H. (2018). The Use of Faecal Microbiota Transplant as Treatment for Recurrent or Refractory Clostridium Difficile Infection and Other Potential Indications: Joint British Society of Gastroenterology (BSG) and Healthcare Infection Society (HIS) Guidelines. Gut.

[347] Gweon T.-G., Lee Y.J., Kim K.O., Yim S.K., Soh J.S., Kim S.Y., Park J.J., Shin S.Y., Lee T.H., Choi C.H. (2022). Clinical Practice Guidelines for Fecal Microbiota Transplantation in Korea. J. Neurogastroenterol. Motil..

[348] Cammarota G., Ianiro G., Tilg H., Rajilić-Stojanović M., Kump P., Satokari R., Sokol H., Arkkila P., Pintus C., Hart A. (2017). European Consensus Conference on Faecal Microbiota Transplantation in Clinical Practice. Gut.

[349] Barberio B., Facchin S., Mele E., D’Incà R., Sturniolo G.C., Farinati F., Zingone F., Quagliariello A., Ghisa M., Massimi D. (2020). Faecal Microbiota Transplantation in Clostridioides Difficile Infection: Real-Life Experience from an Academic Italian Hospital. Ther. Adv. Gastroenterol..

[350] Sun M.-F., Zhu Y.-L., Zhou Z.-L., Jia X.-B., Xu Y.-D., Yang Q., Cui C., Shen Y.-Q. (2018). Neuroprotective Effects of Fecal Microbiota Transplantation on MPTP-Induced Parkinson’s Disease Mice: Gut Microbiota, Glial Reaction and TLR4/TNF-α Signaling Pathway. Brain Behav. Immun..

[351] Smits L.P., Bouter K.E.C., de Vos W.M., Borody T.J., Nieuwdorp M. (2013). Therapeutic Potential of Fecal Microbiota Transplantation. Gastroenterology.

[352] Zhang J., Ma J.-Y., Li Q.-H., Su H., Sun X. (2018). Lactobacillus Rhamnosus GG Induced Protective Effect on Allergic Airway Inflammation Is Associated with Gut Microbiota. Cell Immunol..

[353] El-Salhy M., Hausken T., Hatlebakk J.G. (2019). Increasing the Dose and/or Repeating Faecal Microbiota Transplantation (FMT) Increases the Response in Patients with Irritable Bowel Syndrome (IBS). Nutrients.

[354] Wilson B.C., Derraik J.G.B., Albert B.B., Leong K.S.W., Tweedie-Cullen R.Y., Creagh C., Depczynski M., Edwards T., Vatanen T., Thabrew H. (2023). An Open-Label Pilot Trial of Faecal Microbiome Transfer to Restore the Gut Microbiome in Anorexia Nervosa: Protocol. BMJ Open.

[355] Fan Y., Støving R.K., Ibraim S.B., Hyötyläinen T., Thirion F., Arora T., Lyu L., Stankevic E., Hansen T.H., Déchelotte P. (2023). The Gut Microbiota Contributes to the Pathogenesis of Anorexia Nervosa in Humans and Mice. Nat. Microbiol..

[356] Wu J., Lv L., Wang C. (2022). Efficacy of Fecal Microbiota Transplantation in Irritable Bowel Syndrome: A Meta-Analysis of Randomized Controlled Trials. Front. Cell Infect. Microbiol..

[357] Wilson B.C., Vatanen T., Cutfield W.S., O’Sullivan J.M. (2019). The Super-Donor Phenomenon in Fecal Microbiota Transplantation. Front. Cell Infect. Microbiol..

[358] Dorsaz S., Charretier Y., Girard M., Gaïa N., Leo S., Schrenzel J., Harbarth S., Huttner B., Lazarevic V. (2020). Changes in Microbiota Profiles after Prolonged Frozen Storage of Stool Suspensions. Front. Cell Infect. Microbiol..

[359] Varga A., Kocsis B., Sipos D., Kása P., Vigvári S., Pál S., Dembrovszky F., Farkas K., Péterfi Z. (2021). How to Apply FMT More Effectively, Conveniently and Flexible—A Comparison of FMT Methods. Front. Cell. Infect. Microbiol..

[360] Paramsothy S., Nielsen S., Kamm M.A., Deshpande N.P., Faith J.J., Clemente J.C., Paramsothy R., Walsh A.J., van den Bogaerde J., Samuel D. (2019). Specific Bacteria and Metabolites Associated with Response to Fecal Microbiota Transplantation in Patients with Ulcerative Colitis. Gastroenterology.

[361] Joseph J., Depp C., Shih P.-A.B., Cadenhead K.S., Schmid-Schönbein G. (2017). Modified Mediterranean Diet for Enrichment of Short Chain Fatty Acids: Potential Adjunctive Therapeutic to Target Immune and Metabolic Dysfunction in Schizophrenia?. Front. Neurosci..

[362] Zhu F., Guo R., Wang W., Ju Y., Wang Q., Ma Q., Sun Q., Fan Y., Xie Y., Yang Z. (2020). Transplantation of Microbiota from Drug-Free Patients with Schizophrenia Causes Schizophrenia-like Abnormal Behaviors and Dysregulated Kynurenine Metabolism in Mice. Mol. Psychiatry.

[363] Osaki H., Jodai Y., Koyama K., Omori T., Horiguchi N., Kamano T., Funasaka K., Nagasaka M., Nakagawa Y., Shibata T. (2021). Clinical Response and Changes in the Fecal Microbiota and Metabolite Levels after Fecal Microbiota Transplantation in Patients with Inflammatory Bowel Disease and Recurrent Clostridioides Difficile Infection. Fujita Med. J..

[364] Seekatz A.M., Theriot C.M., Rao K., Chang Y.-M., Freeman A.E., Kao J.Y., Young V.B. (2018). Restoration of Short Chain Fatty Acid and Bile Acid Metabolism Following Fecal Microbiota Transplantation in Patients with Recurrent Clostridium Difficile Infection. Anaerobe.

[365] El-Salhy M., Valeur J., Hausken T., Gunnar Hatlebakk J. (2021). Changes in Fecal Short-Chain Fatty Acids Following Fecal Microbiota Transplantation in Patients with Irritable Bowel Syndrome. Neurogastroenterol. Motil..

[366] Lynch S.V., Pedersen O. (2016). The Human Intestinal Microbiome in Health and Disease. N. Engl. J. Med..

[367] Shock T., Badang L., Ferguson B., Martinez-Guryn K. (2021). The Interplay between Diet, Gut Microbes, and Host Epigenetics in Health and Disease. J. Nutr. Biochem..

[368] Gentile C.L., Weir T.L. (2018). The Gut Microbiota at the Intersection of Diet and Human Health. Science.

[369] Castro-Barquero S., Ruiz-León A.M., Sierra-Pérez M., Estruch R., Casas R. (2020). Dietary Strategies for Metabolic Syndrome: A Comprehensive Review. Nutrients.

[370] Shi H., Ge X., Ma X., Zheng M., Cui X., Pan W., Zheng P., Yang X., Zhang P., Hu M. (2021). A Fiber-Deprived Diet Causes Cognitive Impairment and Hippocampal Microglia-Mediated Synaptic Loss through the Gut Microbiota and Metabolites. Microbiome.

[371] Ristori M.V., Quagliariello A., Reddel S., Ianiro G., Vicari S., Gasbarrini A., Putignani L. (2019). Autism, Gastrointestinal Symptoms and Modulation of Gut Microbiota by Nutritional Interventions. Nutrients.

[372] Adolph T.E., Zhang J. (2022). Diet Fuelling Inflammatory Bowel Diseases: Preclinical and Clinical Concepts. Gut.

[373] Opie R.S., O’Neil A., Jacka F.N., Pizzinga J., Itsiopoulos C. (2018). A Modified Mediterranean Dietary Intervention for Adults with Major Depression: Dietary Protocol and Feasibility Data from the SMILES Trial. Nutr. Neurosci..

[374] Pylkas A.M., Juneja L.R., Slavin J.L. (2005). Comparison of Different Fibers for In Vitro Production of Short Chain Fatty Acids by Intestinal Microflora. J. Med. Food.

[375] Vinelli V., Biscotti P., Martini D., Del Bo’ C., Marino M., Meroño T., Nikoloudaki O., Calabrese F.M., Turroni S., Taverniti V. (2022). Effects of Dietary Fibers on Short-Chain Fatty Acids and Gut Microbiota Composition in Healthy Adults: A Systematic Review. Nutrients.

[376] Bonazzi E., Bretin A., Vigué L., Hao F., Patterson A.D., Gewirtz A.T., Chassaing B. (2024). Individualized Microbiotas Dictate the Impact of Dietary Fiber on Colitis Sensitivity. Microbiome.

[377] Gudan A., Skonieczna-Żydecka K., Palma J., Drozd A., Stachowska E. (2022). Effects of Dietary Components on Intestinal Short-Chain Fatty Acids (SCFAs) Synthesis in Healthy Adult Persons Following a Ketogenic Diet. Rocz. Panstw. Zakl. Hig..

[378] Seethaler B., Nguyen N.K., Basrai M., Kiechle M., Walter J., Delzenne N.M., Bischoff S.C. (2022). Short-Chain Fatty Acids Are Key Mediators of the Favorable Effects of the Mediterranean Diet on Intestinal Barrier Integrity: Data from the Randomized Controlled LIBRE Trial. Am. J. Clin. Nutr..

[379] Tan J.K., Macia L., Mackay C.R. (2023). Dietary Fiber and SCFAs in the Regulation of Mucosal Immunity. J. Allergy Clin. Immunol..

[380] François I.E.J.A., Lescroart O., Veraverbeke W.S., Marzorati M., Possemiers S., Evenepoel P., Hamer H., Houben E., Windey K., Welling G.W. (2012). Effects of a Wheat Bran Extract Containing Arabinoxylan Oligosaccharides on Gastrointestinal Health Parameters in Healthy Adult Human Volunteers: A Double-Blind, Randomised, Placebo-Controlled, Cross-over Trial. Br. J. Nutr..

[381] Kovatcheva-Datchary P., Nilsson A., Akrami R., Lee Y.S., De Vadder F., Arora T., Hallen A., Martens E., Björck I., Bäckhed F. (2015). Dietary Fiber-Induced Improvement in Glucose Metabolism Is Associated with Increased Abundance of Prevotella. Cell Metab..

[382] Nilsson A.C., Johansson-Boll E.V., Björck I.M.E. (2015). Increased Gut Hormones and Insulin Sensitivity Index Following a 3-d Intervention with a Barley Kernel-Based Product: A Randomised Cross-over Study in Healthy Middle-Aged Subjects. Br. J. Nutr..

[383] Wang Y., Ames N.P., Tun H.M., Tosh S.M., Jones P.J., Khafipour E. (2016). High Molecular Weight Barley β-Glucan Alters Gut Microbiota toward Reduced Cardiovascular Disease Risk. Front. Microbiol..

[384] Farup P.G., Valeur J. (2020). Changes in Faecal Short-Chain Fatty Acids after Weight-Loss Interventions in Subjects with Morbid Obesity. Nutrients.

[385] Salminen S., Collado M.C., Endo A., Hill C., Lebeer S., Quigley E.M.M., Sanders M.E., Shamir R., Swann J.R., Szajewska H. (2021). The International Scientific Association of Probiotics and Prebiotics (ISAPP) Consensus Statement on the Definition and Scope of Postbiotics. Nat. Rev. Gastroenterol. Hepatol..

[386] Cunningham M., Azcarate-Peril M.A., Barnard A., Benoit V., Grimaldi R., Guyonnet D., Holscher H.D., Hunter K., Manurung S., Obis D. (2021). Shaping the Future of Probiotics and Prebiotics. Trends Microbiol..

[387] Monteagudo-Mera A., Rastall R.A., Gibson G.R., Charalampopoulos D., Chatzifragkou A. (2019). Adhesion Mechanisms Mediated by Probiotics and Prebiotics and Their Potential Impact on Human Health. Appl. Microbiol. Biotechnol..

[388] Wieërs G., Belkhir L., Enaud R., Leclercq S., de Foy J.-M.P., Dequenne I., de Timary P., Cani P.D. (2020). How Probiotics Affect the Microbiota. Front. Cell. Infect. Microbiol..

[389] Gibson G.R., Hutkins R., Sanders M.E., Prescott S.L., Reimer R.A., Salminen S.J., Scott K., Stanton C., Swanson K.S., Cani P.D. (2017). Expert Consensus Document: The International Scientific Association for Probiotics and Prebiotics (ISAPP) Consensus Statement on the Definition and Scope of Prebiotics. Nat. Rev. Gastroenterol. Hepatol..

[390] Zheng D.-W., Li R.-Q., An J.-X., Xie T.-Q., Han Z.-Y., Xu R., Fang Y., Zhang X.-Z. (2020). Prebiotics-Encapsulated Probiotic Spores Regulate Gut Microbiota and Suppress Colon Cancer. Adv. Mater..

[391] Dalile B., Van Oudenhove L., Vervliet B., Verbeke K. (2019). The Role of Short-Chain Fatty Acids in Microbiota-Gut-Brain Communication. Nat. Rev. Gastroenterol. Hepatol..

[392] Sawin E.A., De Wolfe T.J., Aktas B., Stroup B.M., Murali S.G., Steele J.L., Ney D.M. (2015). Glycomacropeptide Is a Prebiotic That Reduces Desulfovibrio Bacteria, Increases Cecal Short-Chain Fatty Acids, and Is Anti-Inflammatory in Mice. Am. J. Physiol. Gastrointest. Liver Physiol..

[393] Holmes Z.C., Villa M.M., Durand H.K., Jiang S., Dallow E.P., Petrone B.L., Silverman J.D., Lin P.-H., David L.A. (2022). Microbiota Responses to Different Prebiotics Are Conserved within Individuals and Associated with Habitual Fiber Intake. Microbiome.

[394] Ojo O., Feng Q.-Q., Ojo O.O., Wang X.-H. (2020). The Role of Dietary Fibre in Modulating Gut Microbiota Dysbiosis in Patients with Type 2 Diabetes: A Systematic Review and Meta-Analysis of Randomised Controlled Trials. Nutrients.

[395] Birkeland E., Gharagozlian S., Birkeland K.I., Valeur J., Måge I., Rud I., Aas A.-M. (2020). Prebiotic Effect of Inulin-Type Fructans on Faecal Microbiota and Short-Chain Fatty Acids in Type 2 Diabetes: A Randomised Controlled Trial. Eur. J. Nutr..

[396] Birkeland E., Gharagozlian S., Gulseth H.L., Birkeland K.I., Hartmann B., Holst J.J., Holst R., Aas A.-M. (2021). Effects of Prebiotics on Postprandial GLP-1, GLP-2 and Glucose Regulation in Patients with Type 2 Diabetes: A Randomised, Double-Blind, Placebo-Controlled Crossover Trial. Diabet. Med..

[397] Hughes R.L., Alvarado D.A., Swanson K.S., Holscher H.D. (2022). The Prebiotic Potential of Inulin-Type Fructans: A Systematic Review. Adv. Nutr..

[398] Li L., Li P., Xu L. (2021). Assessing the Effects of Inulin-Type Fructan Intake on Body Weight, Blood Glucose, and Lipid Profile: A Systematic Review and Meta-Analysis of Randomized Controlled Trials. Food Sci. Nutr..

[399] Łoniewski I., Szulińska M., Kaczmarczyk M., Podsiadło K., Styburski D., Skonieczna-Żydecka K., Bogdański P. (2023). Multispecies Probiotic Affects Fecal Short-Chain Fatty Acids in Postmenopausal Women with Obesity: A Post Hoc Analysis of a Randomized, Double-Blind, Placebo-Controlled Study. Nutrition.

[400] Rad Z.A., Mousavi S.N., Chiti H. (2023). A Low-Carb Diet Increases Fecal Short-Chain Fatty Acids in Feces of Obese Women Following a Weight-Loss Program: Randomized Feeding Trial. Sci. Rep..

